# Supplementary material for: Integrated CO2 capture-fixation chemistry via interfacial ionic liquid catalyst in laminar gas/liquid flow
Source: Nat Commun. 2017 Mar 6;8:14676. doi: 10.1038/ncomms14676 (PMC5343516; doi:10.1038/ncomms14676)
Supplement: Supplementary Information — Supplementary figures, supplementary tables, supplementary methods and supplementary references. [file ncomms14676-s1.pdf]

Supplementary Figures

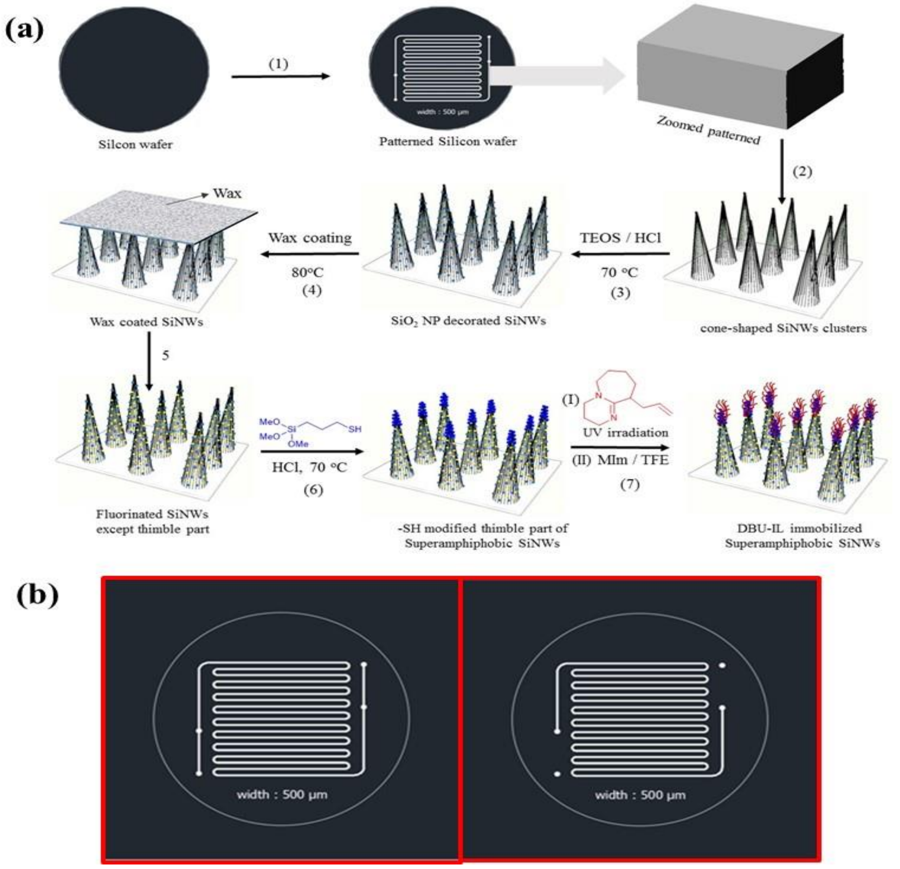

Supplementary Figure 1. (a) Fabrication of DBU-ILs immobilized superamphiphobic SiNWs clusters. *Step 1*: Patterning of protective AZ photoresist layer on Si wafer by photolithography process. *Step 2*: Preparation of SiNWs pattern by anisotropic etching. *Step 3*: SiO_2 nanoparticles coating over the whole SiNWs. *Step 4*: Selective wax coating only on the thimble of SiNWs. *Step 5*: Fluorination through CVD on SiNWs surface. *Step 6*: Selective mercaptosilane coating only on thimble part. *Step 7:* DBU-IL immobilization by thiol-ene click reaction of –SH modified thimble part and A-DBU followed by THF solution of Mim or TFE; **(b)** Photomask patterns used for serpentine SiNWs microreactor composed of SiNWs bottom channel (left) and PDMS upper channel (right).

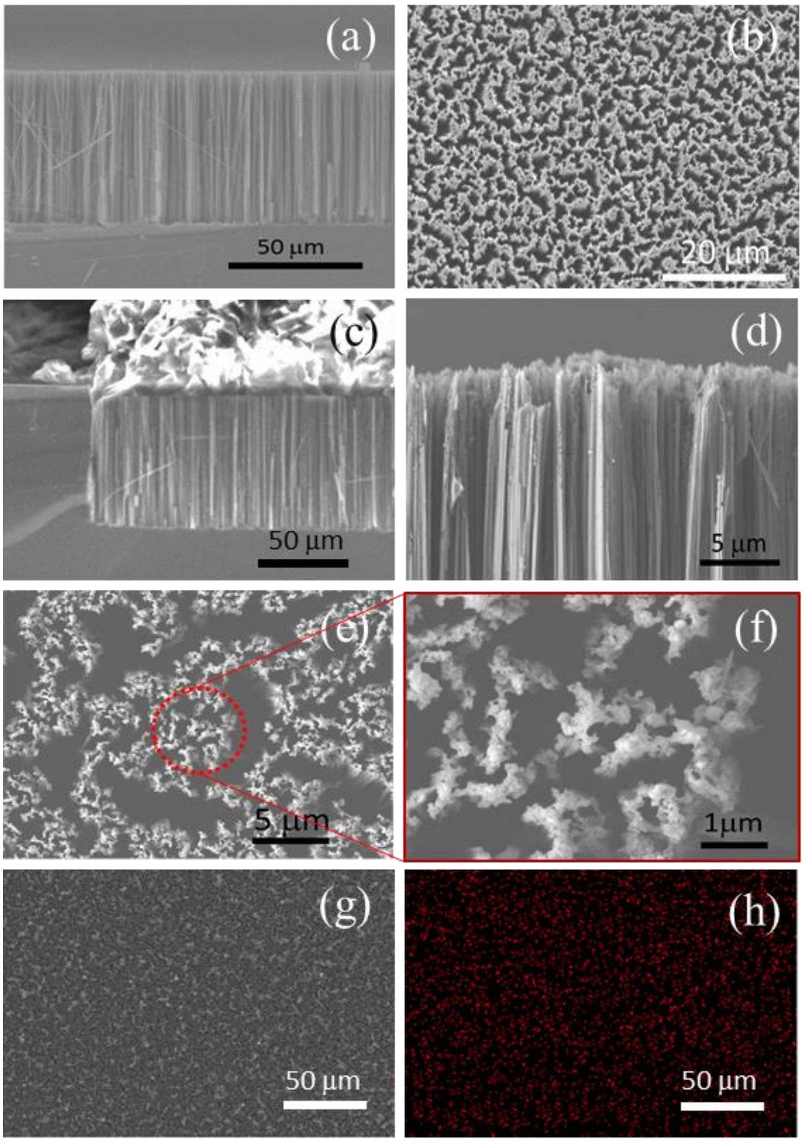

Supplementary Figure 2. SEM images of bare SiNWs cluster, (a) cross-sectional view; (b) top view; (c) SEM image of wax coated cross-sectional view of SiO_2 NP decorated SiNWs. SEM images of DBU-ILs immobilized SiNWs; (d) cross-sectional view; (e, f) top view; (g) SEM image of top view of mercaptosilane immobilized SiNWs and (h) corresponding sulphur EDX-mapping.

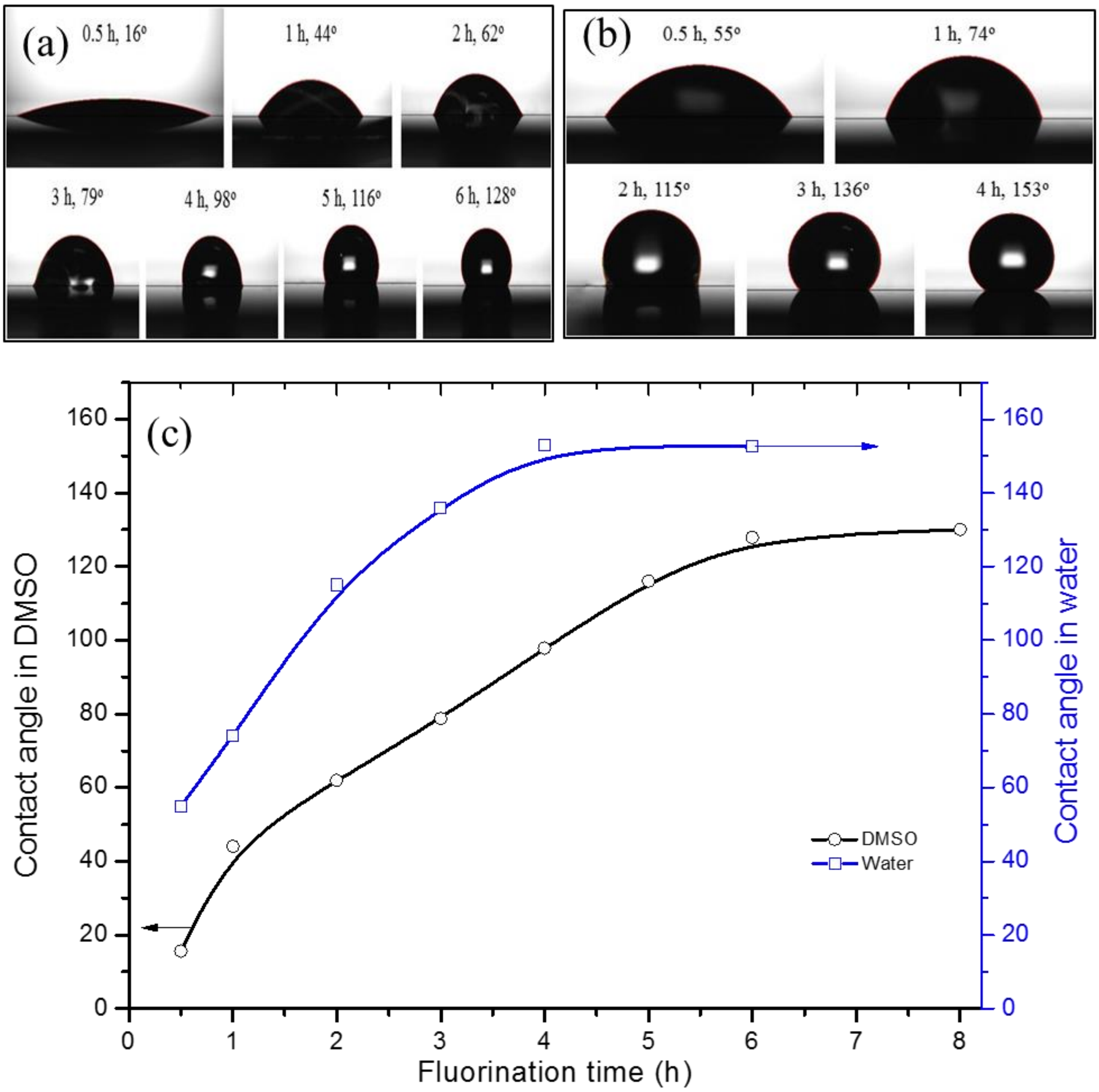

Supplementary Figure 3. Contact angle variation under CVD fluorination process (trichloro(1*H*,1*H*,2*H*,2*H*-perfluorooctyl)silane treatment at 55 ^{o}C) with varied time, (a) Static contact angle photograph under DMSO. (b) Static contact angle photograph under water; (c) Comparative variation of contact angle with varied CVD fluorination time.

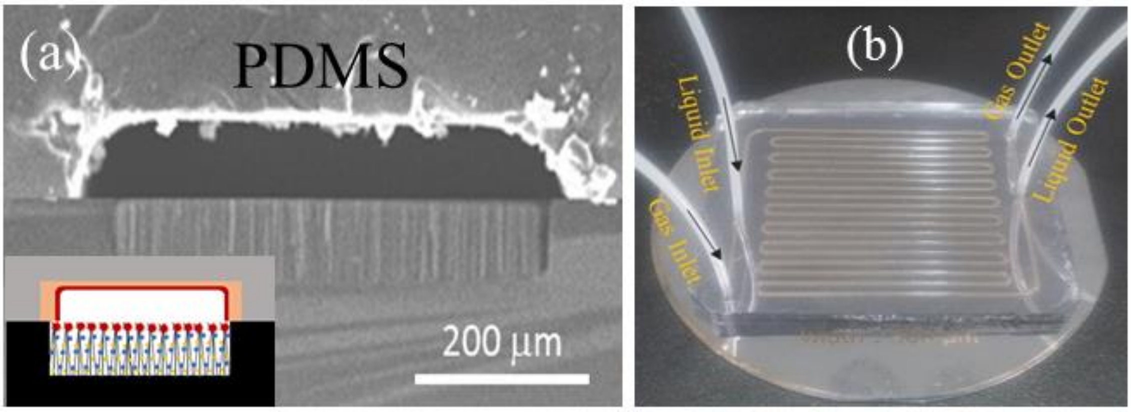

Supplementary Figure 4. (**a**) Cross-sectional SEM image of DBU-ILs immobilized SiNWs microreactor and schematic illustration (inset). Scale bar, 200 μm. (**b**) Optical image of fabricated microreactor showing two inlets and two outlets. Scale bar, 2 cm.

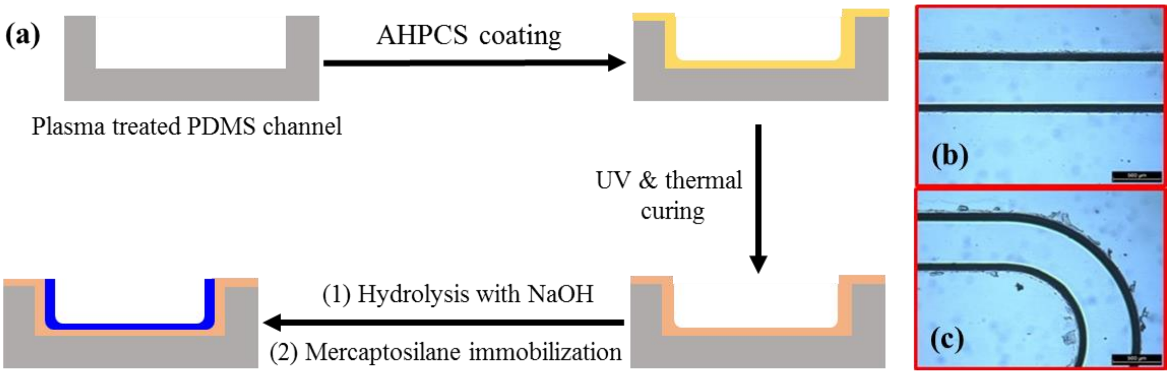

Supplementary Figure 5. (a) Schematic illustration of fabrication of the AHPCS modified PDMS channel and surface modification by mercaptosilane after hydrolysis with 0.5N NaOH solution for 2 h at 30 °C; (b) Optical microscope images of the AHPCS coated PDMS straight channel; (c) curved part of the serpentine channel.

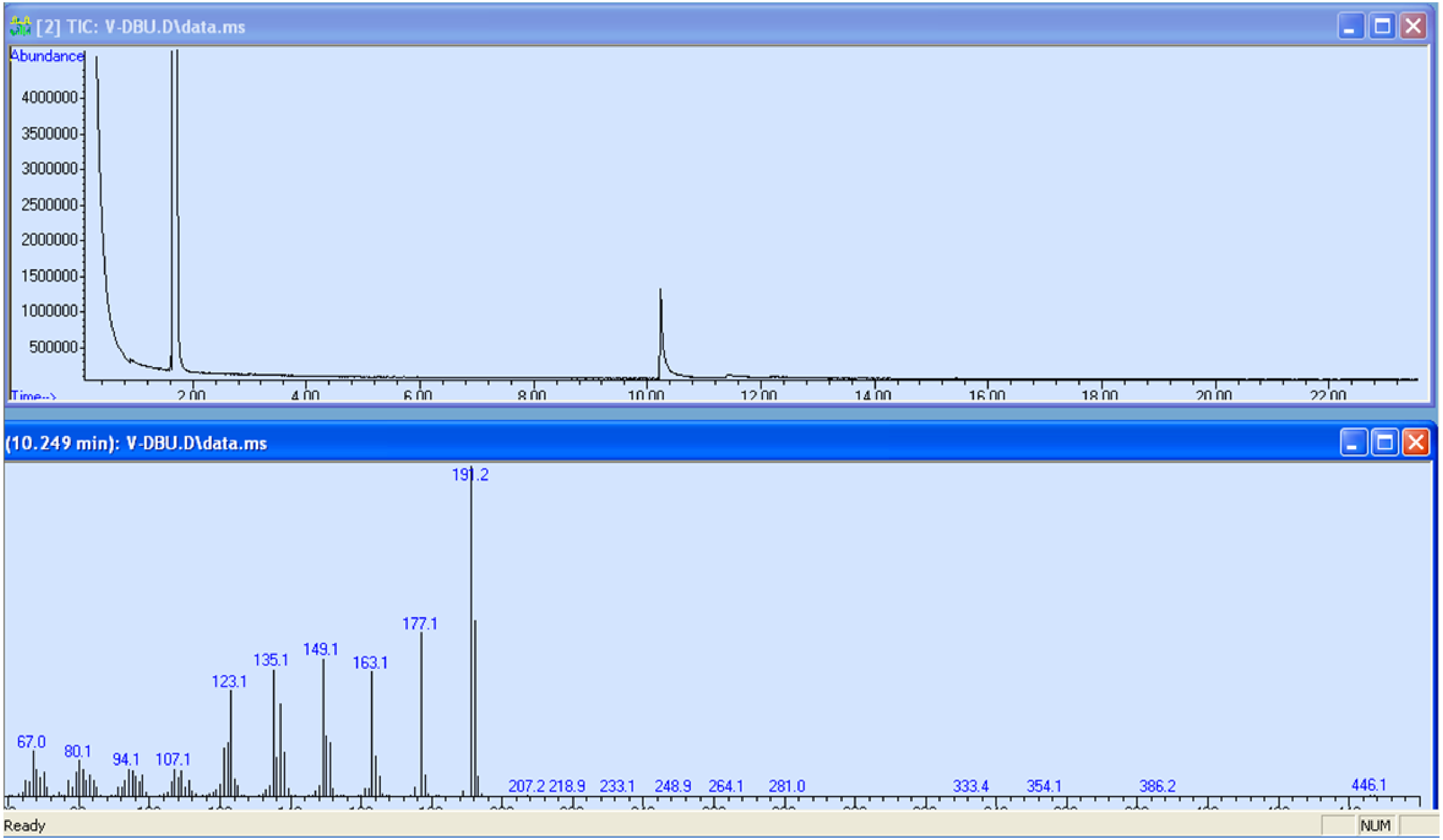

Supplementary Figure 6. GC-MS of 6-allyl-1,8-diazabicyclo[5.4.0]undec-7-ene (A-DBU).

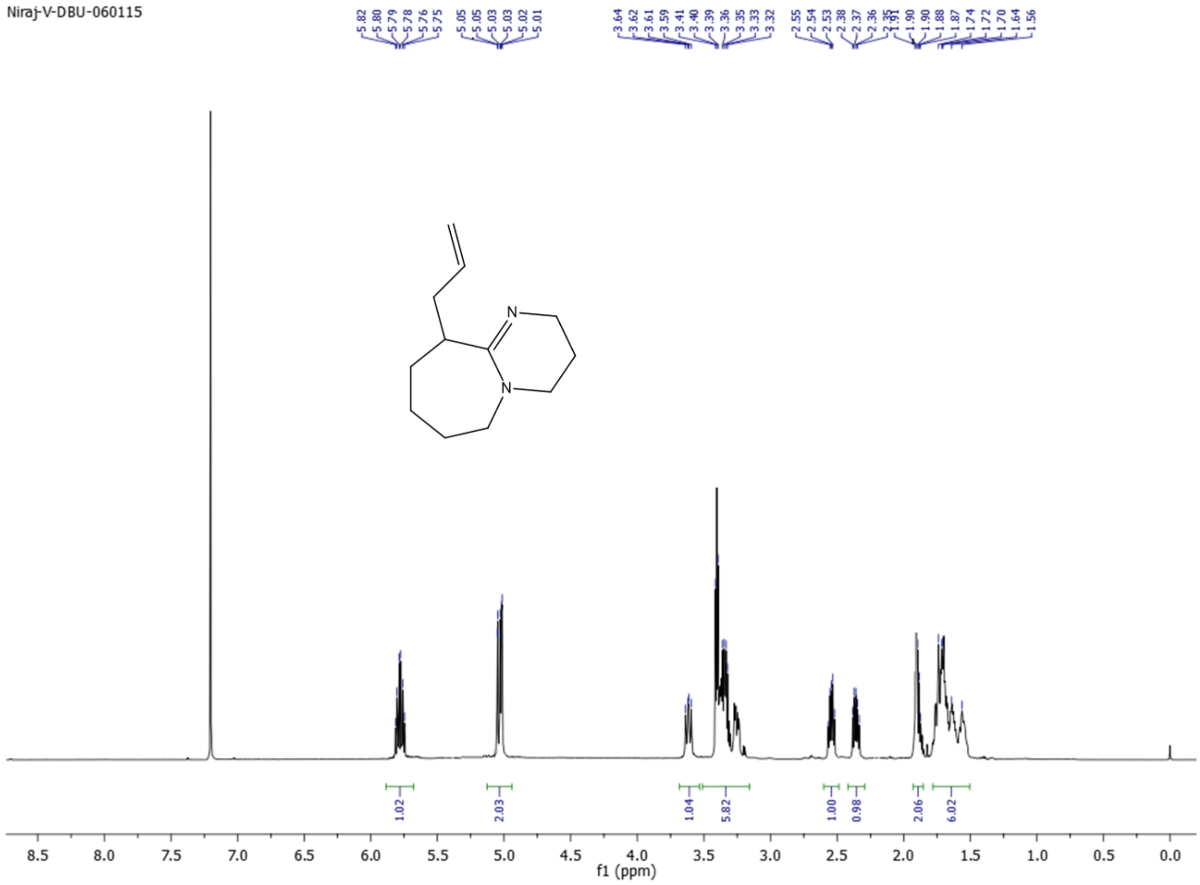

Supplementary Figure 7. ^{1}H NMR spectra of A-DBU in $CDCl_3$.

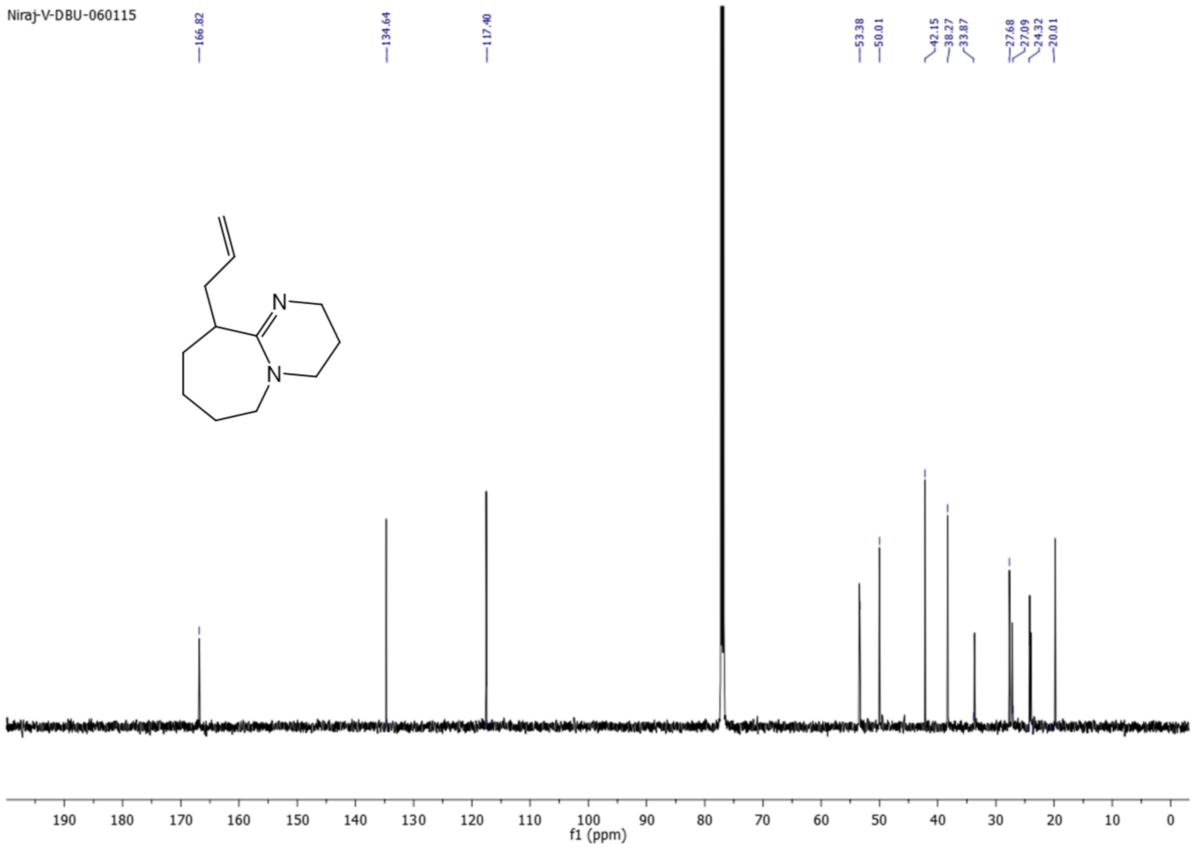

Supplementary Figure 8. ^{13}C NMR spectra of A-DBU in $CDCl_3$.

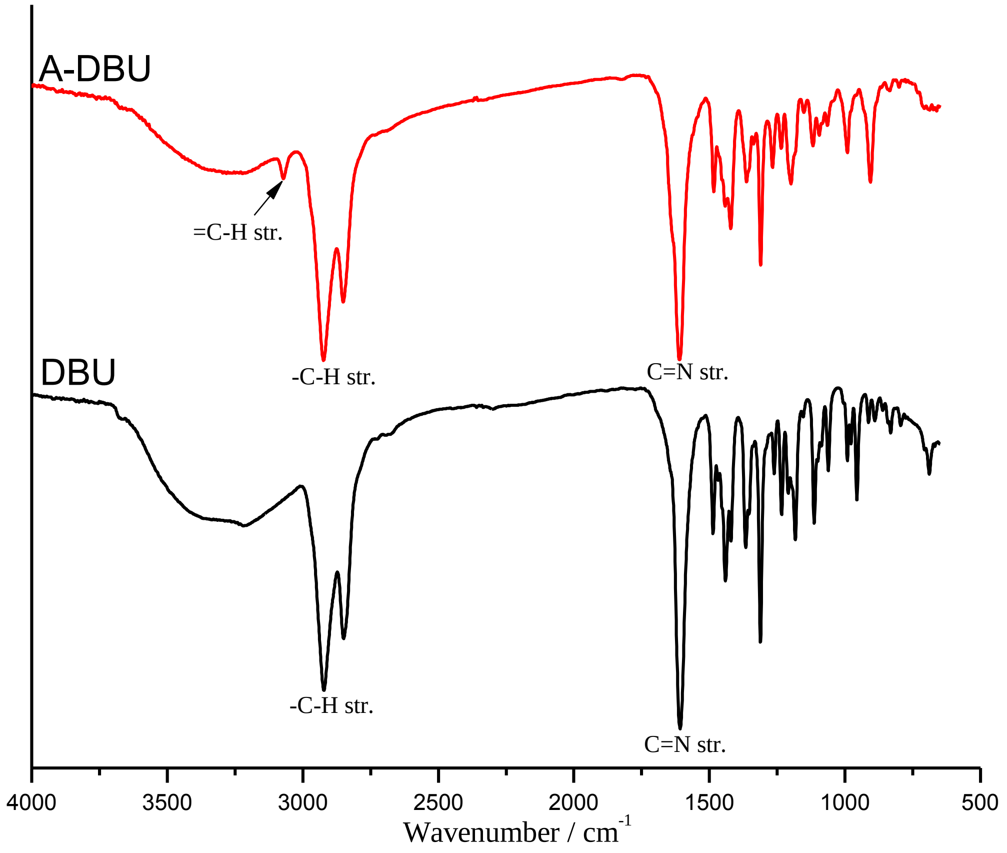

Supplementary Figure 9. Comparative FTIR spectra of A-DBU and DBU.

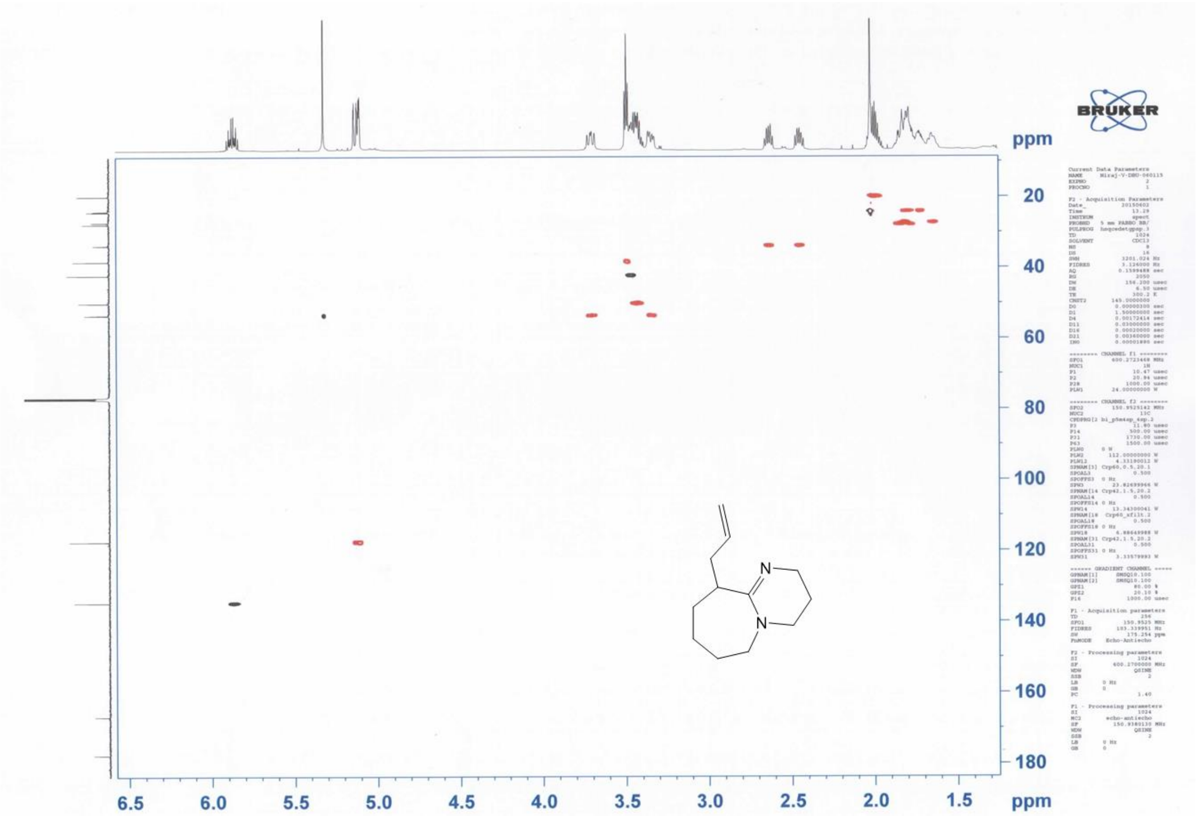

Supplementary Figure 10. C-H HSQC NMR (Heteronuclear Single Quantum Coherence Nuclear Magnetic Resonance spectroscopy) spectra of monomer A-DBU in $CDCl_3$.

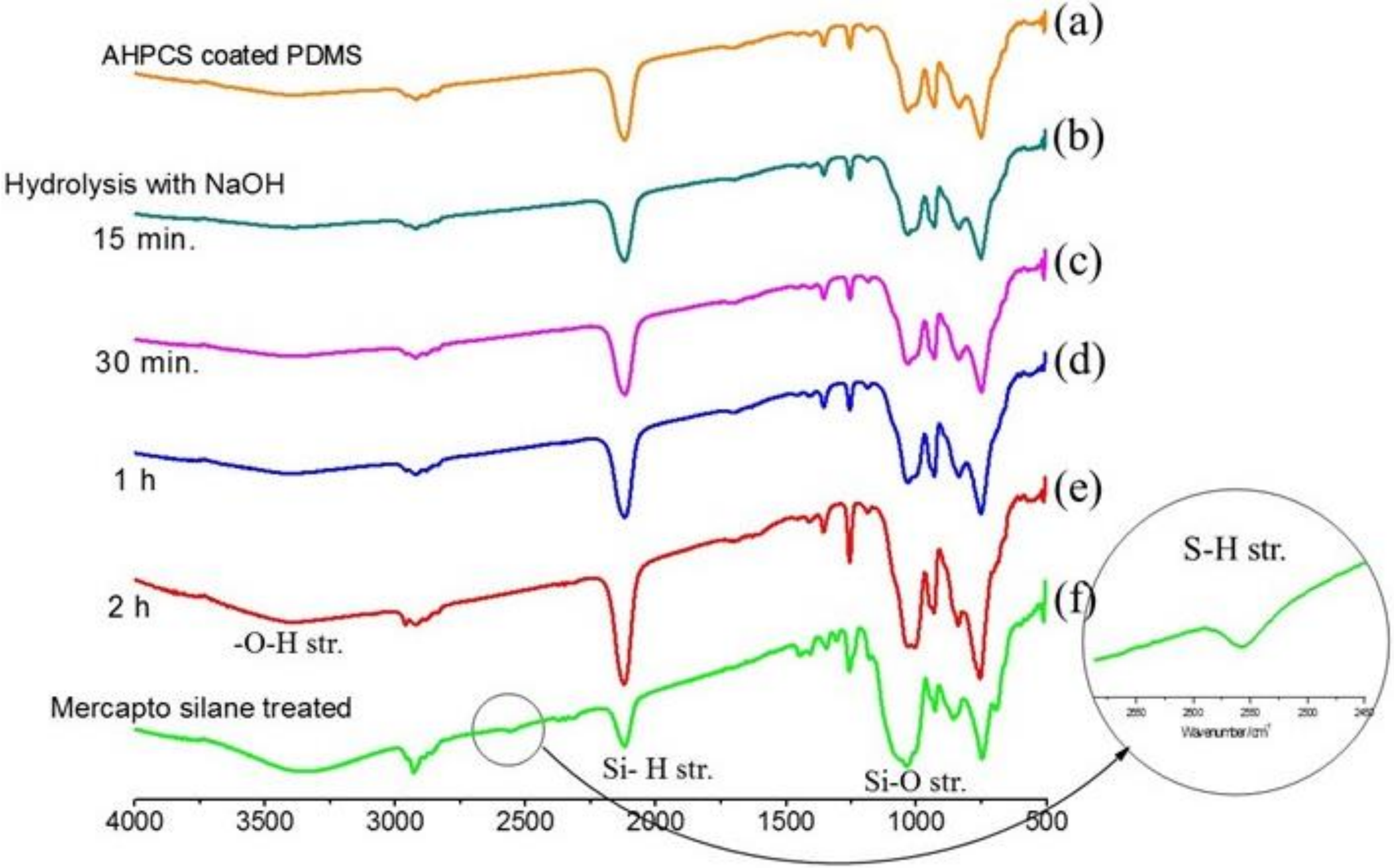

Supplementary Figure 11. FTIR spectra of (a) AHPCS coated PDMS surface, (b-e) AHPCS surface time dependent hydrolysis by 0.5N NaOH and (f) AHPCS surface after mercaptosilane immobilization (-SH modified).

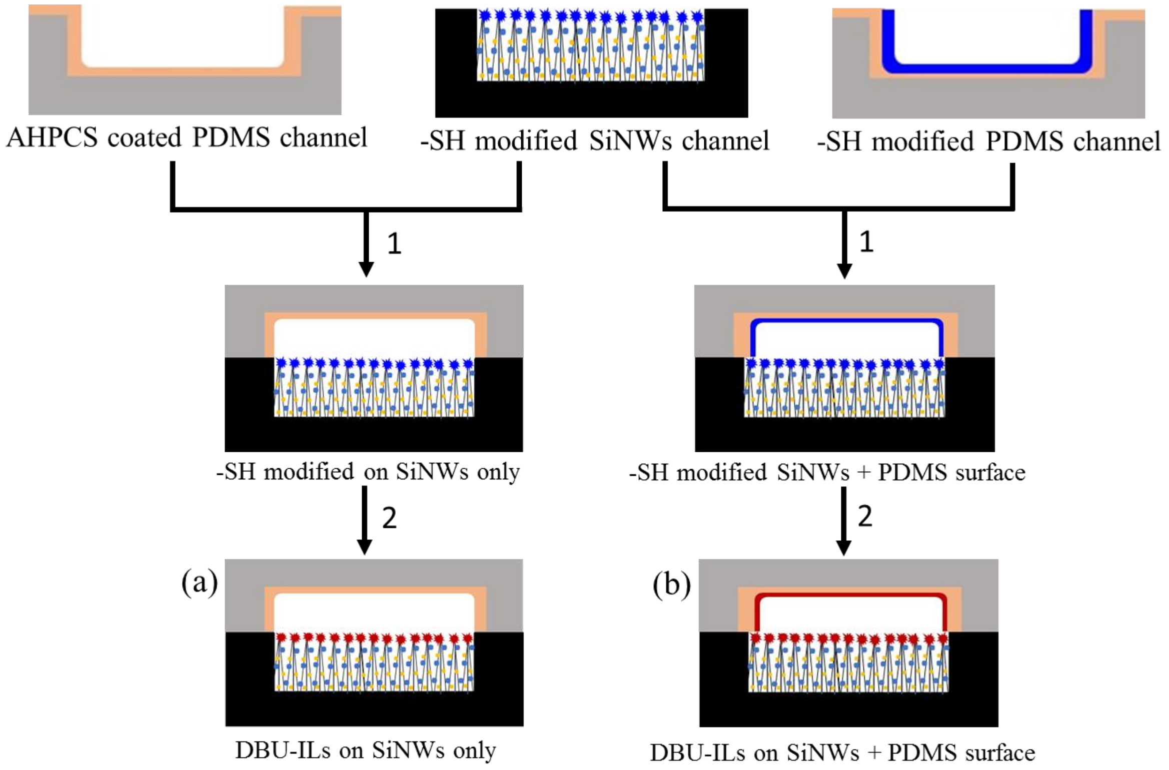

Supplementary Figure 12. Schematic illustration of fabrication of DBU-ILs immobilized SiNWs microreactor, *Step 1:* Bonding of mercaptosilane immobilized SiNWs channel and PDMS channel after plasma treatment; *Step 2:* Immobilization of DBU-ILs by thiol-ene click reaction; (a) only on thimble part of SiNWs, (b) both on thimble part and the AHPCS modified PDMS wall surface.

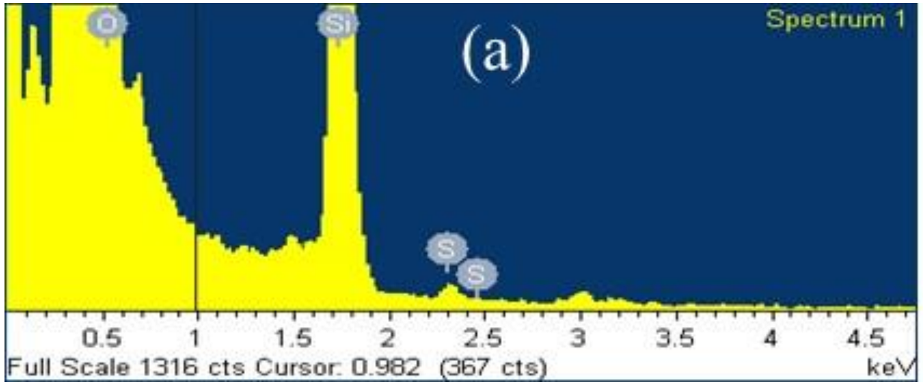

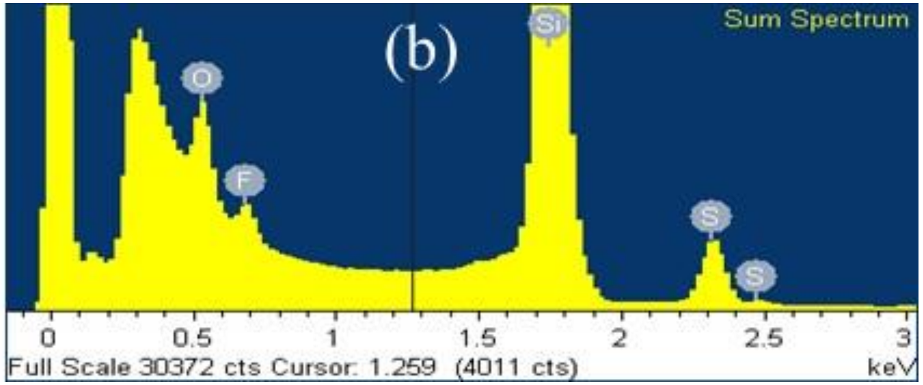

Supplementary Figure 13. EDX spectra of (a) [HDBU$^+$][MIm$^-$]-ILs immobilized SiNWs and (b) selective mercaptosilane immobilized SiNWs for [HDBU$^+$][TFE$^-$]-ILs.

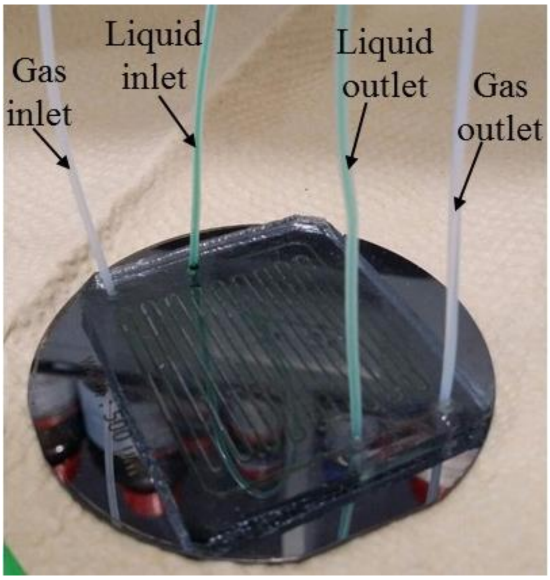

Supplementary Figure 14. Real time snapshot of the supplementary video (Supplementary Movie 1) for gas-liquid stable laminar flow.

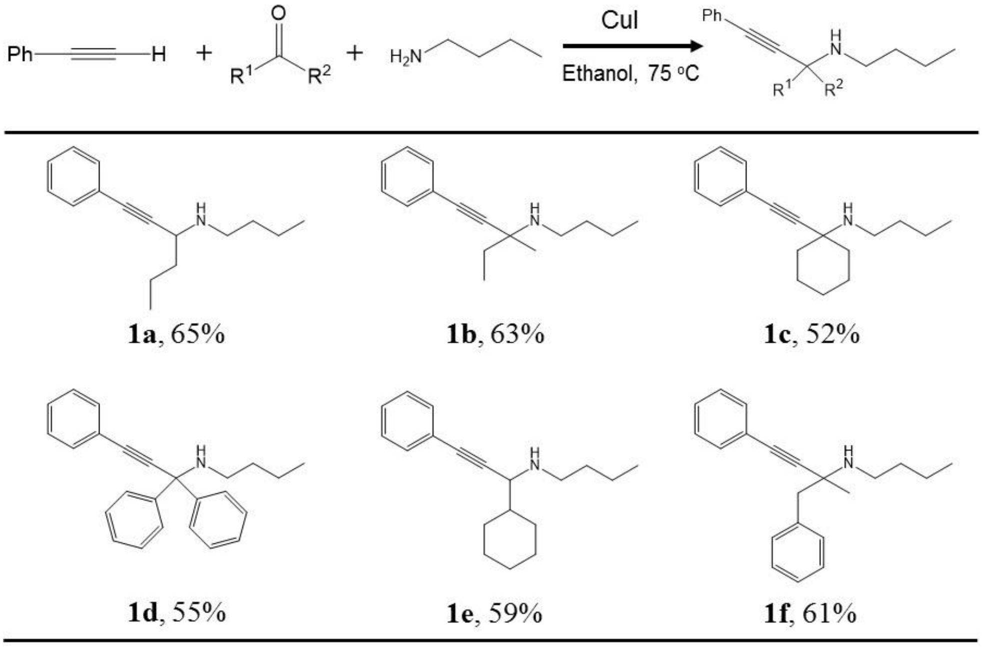

Phenylacetylene (10 mmol), Aldehyde/ketone (20 mmol), Amine (20 mmol), CuI (3 mmol) at 80 °C for overnight

Supplementary Figure 15. Schematic presentation of synthesis of various propargylic amine derivatives for 2-oxazolidinones production.

^{1}H & ^{13}C NMR spectra of synthesized propargylic amines (1a-1f):

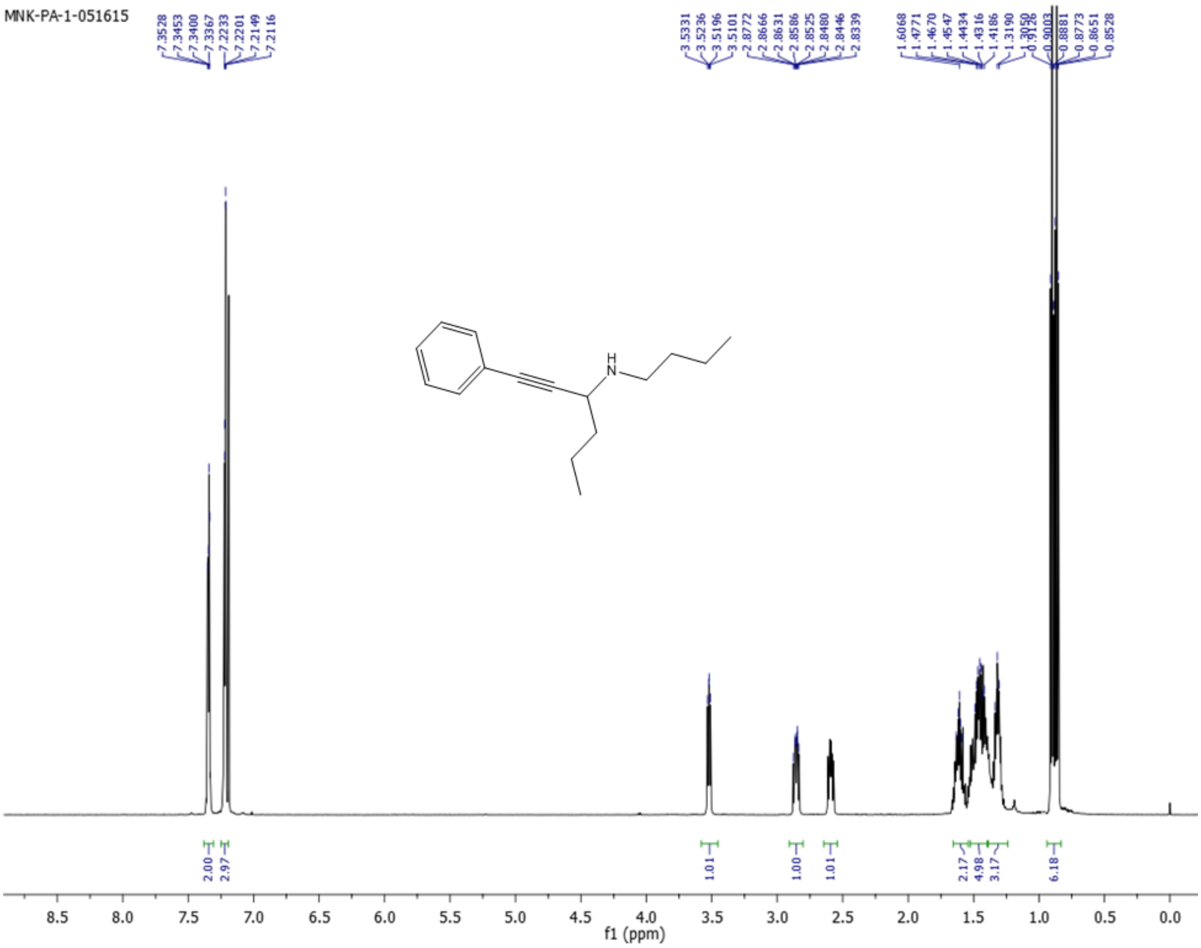

Supplementary Figure 16. ^{1}H NMR Spectra of butyl-(1-phenylethynyl-butyl)-amine in $CDCl_3$.

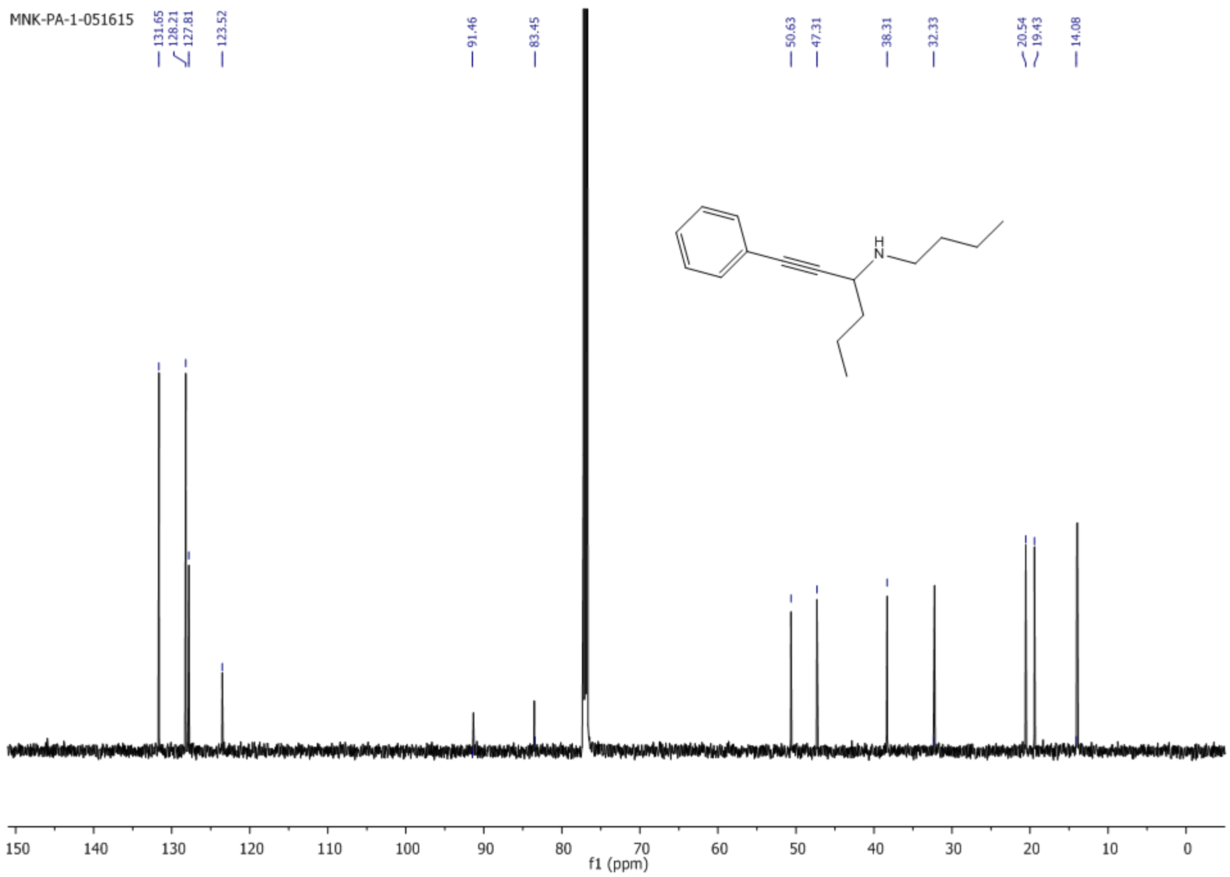

Supplementary Figure 17. ^{13}C NMR Spectra of butyl-(1-phenylethynyl-butyl)-amine in $CDCl_3$.

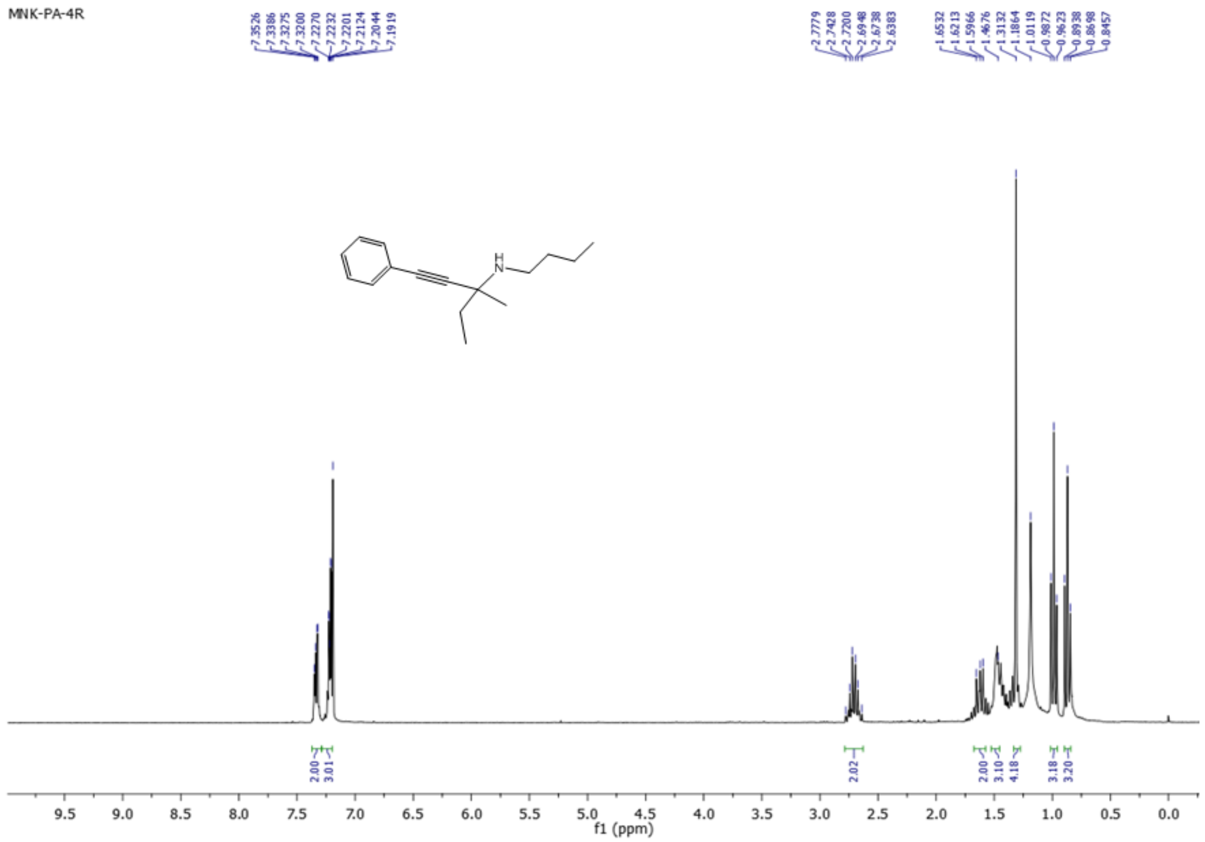

Supplementary Figure 18. ^{1}H NMR Spectra of Butyl-(1-ethyl-1-methyl-3-phenylprop-2-ynyl)-amine in $CDCl_3$.

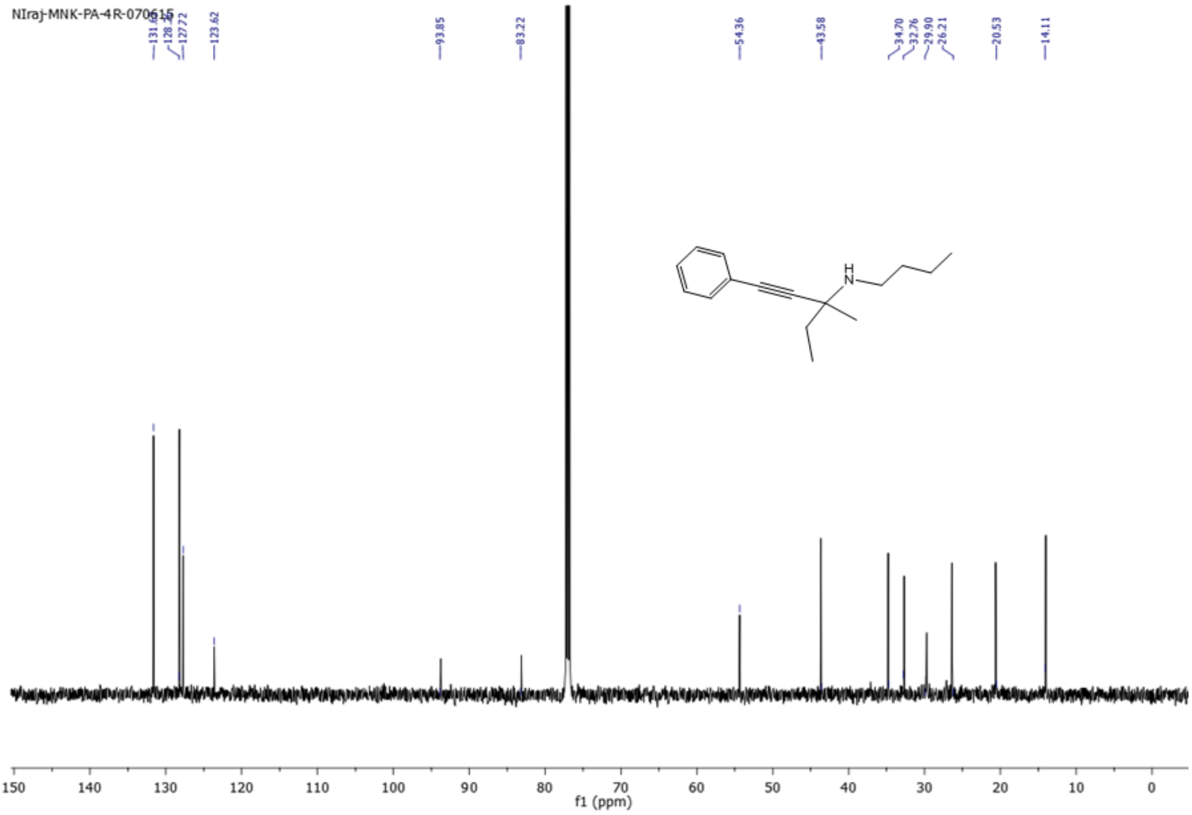

Supplementary Figure 19. ^{13}C NMR Spectra of Butyl-(1-ethyl-1-methyl-3-phenylprop-2-ynyl)-amine in $CDCl_3$.

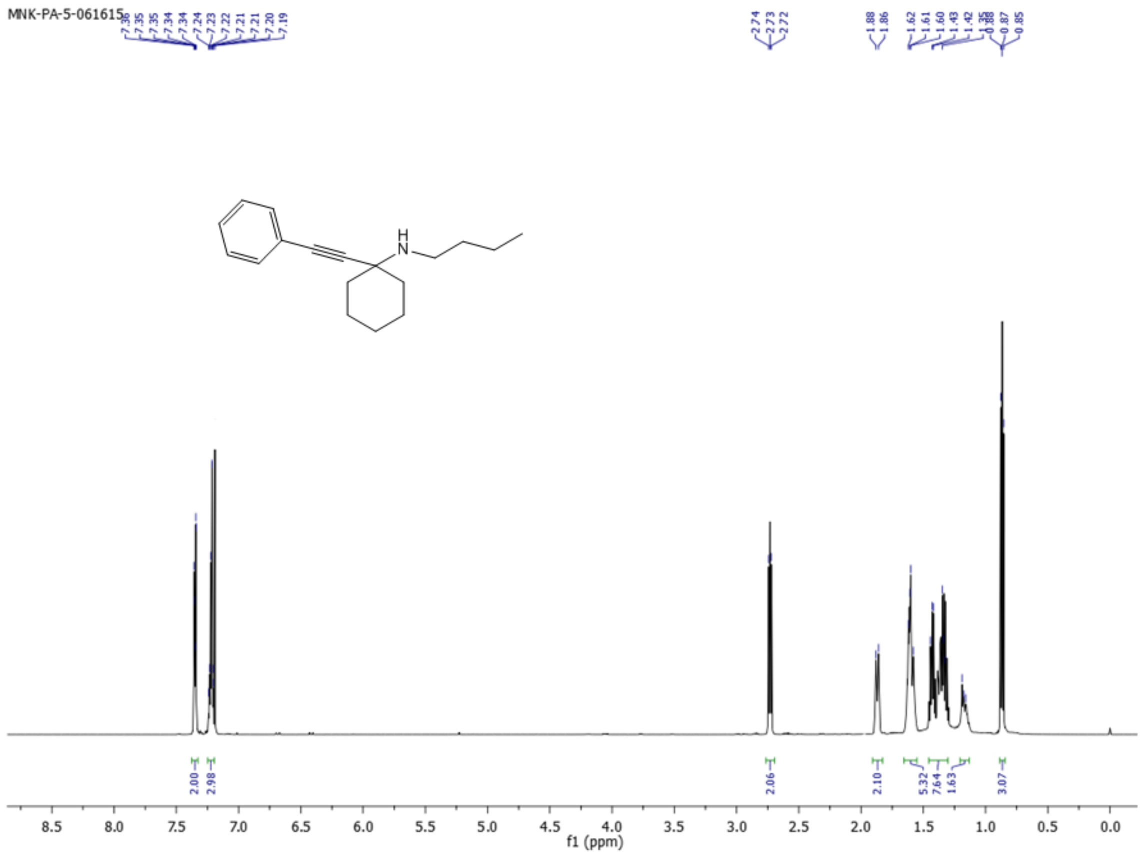

Supplementary Figure 20. ^{1}H NMR Spectra of butyl-(1-phenylethynyl-cyclohexyl)-amine in $CDCl_3$.

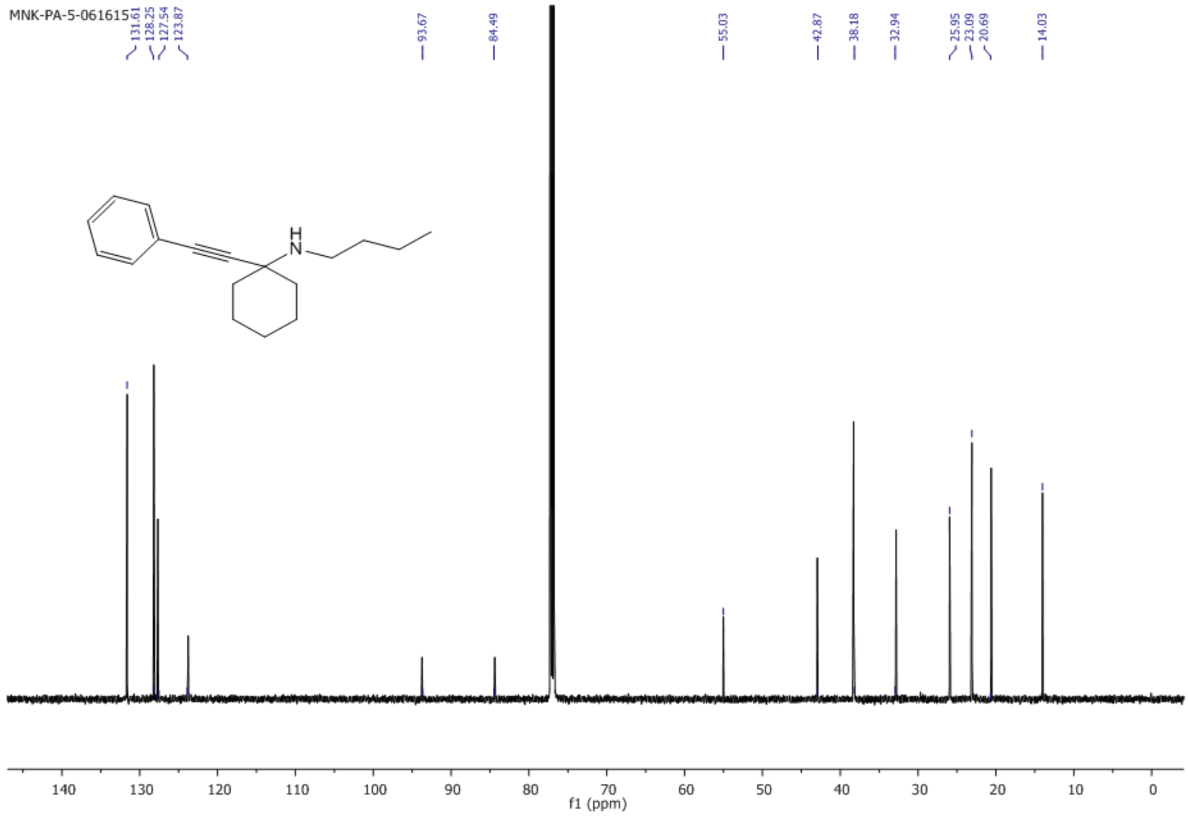

Supplementary Figure 21. ^{13}C NMR Spectra of butyl-(1-phenylethynyl-cyclohexyl)-amine in $CDCl_3$.

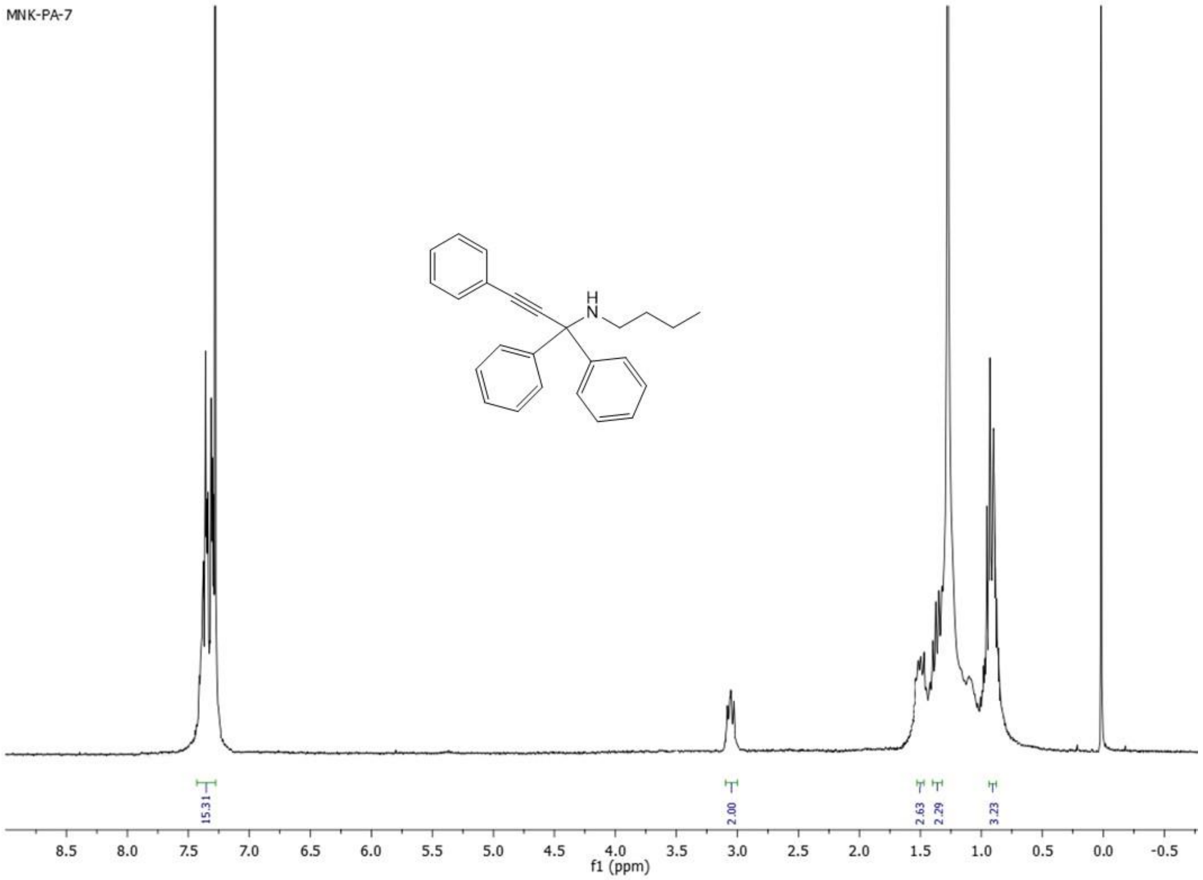

Supplementary Figure 22. ^{1}H NMR Spectra of *N*-(1,1,3-triphenylprop-2-yn-1-yl)butan-1-amine in $CDCl_3$.

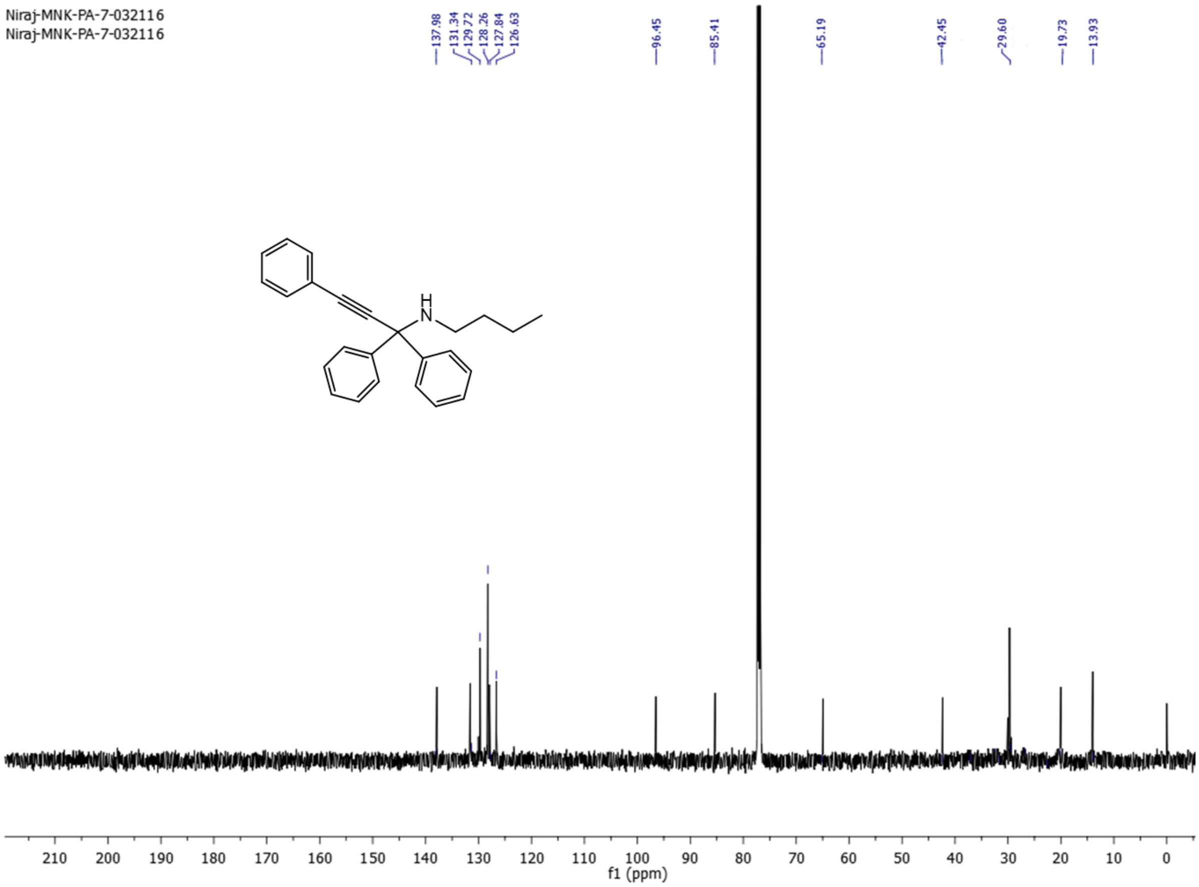

Supplementary Figure 23. ^{13}C NMR Spectra of *N*-(1,1,3-triphenylprop-2-yn-1-yl)butan-1-amine in $CDCl_3$.

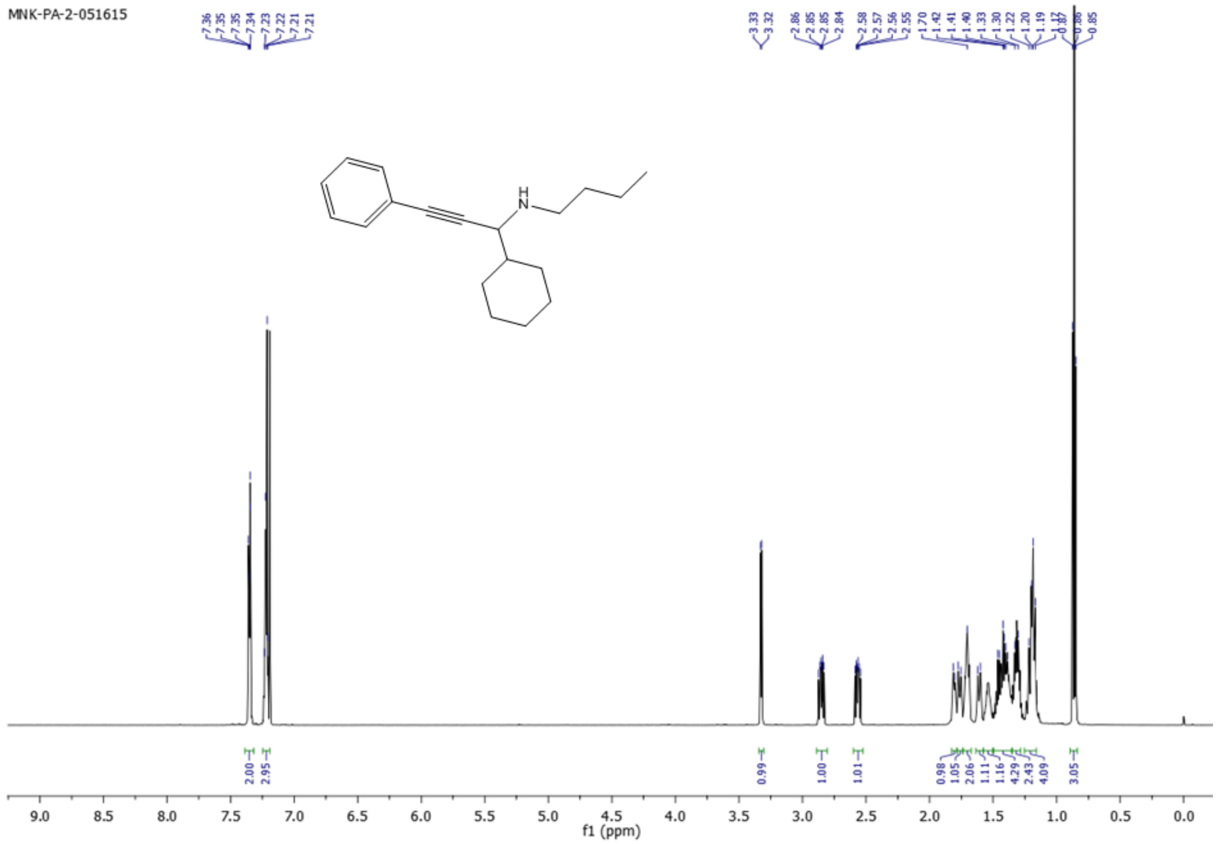

Supplementary Figure 24. ^{1}H NMR Spectra of butyl-(1-cyclohexyl-3-phenyl-prop-2-ynyl)-amine in $CDCl_3$.

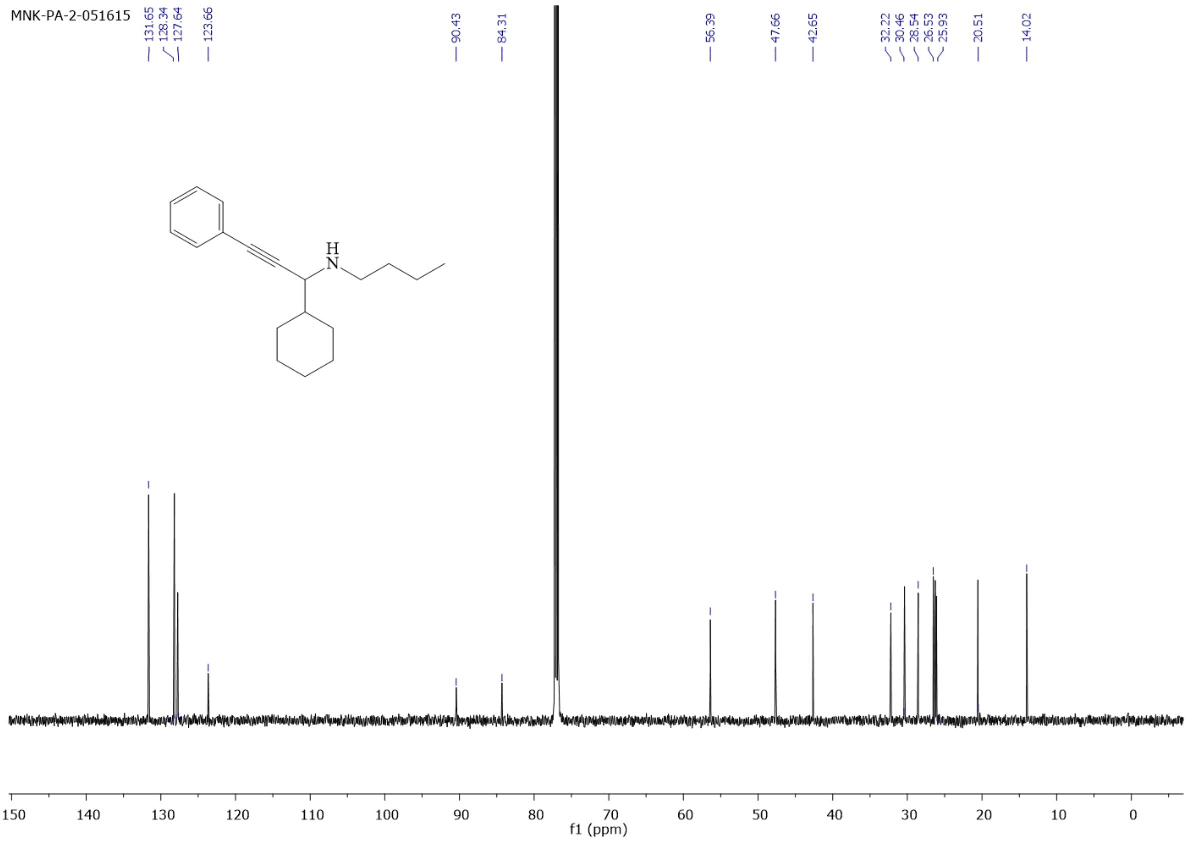

Supplementary Figure 25. ^{13}C NMR Spectra of butyl-(1-cyclohexyl-3-phenyl-prop-2-ynyl)-amine in $CDCl_3$.

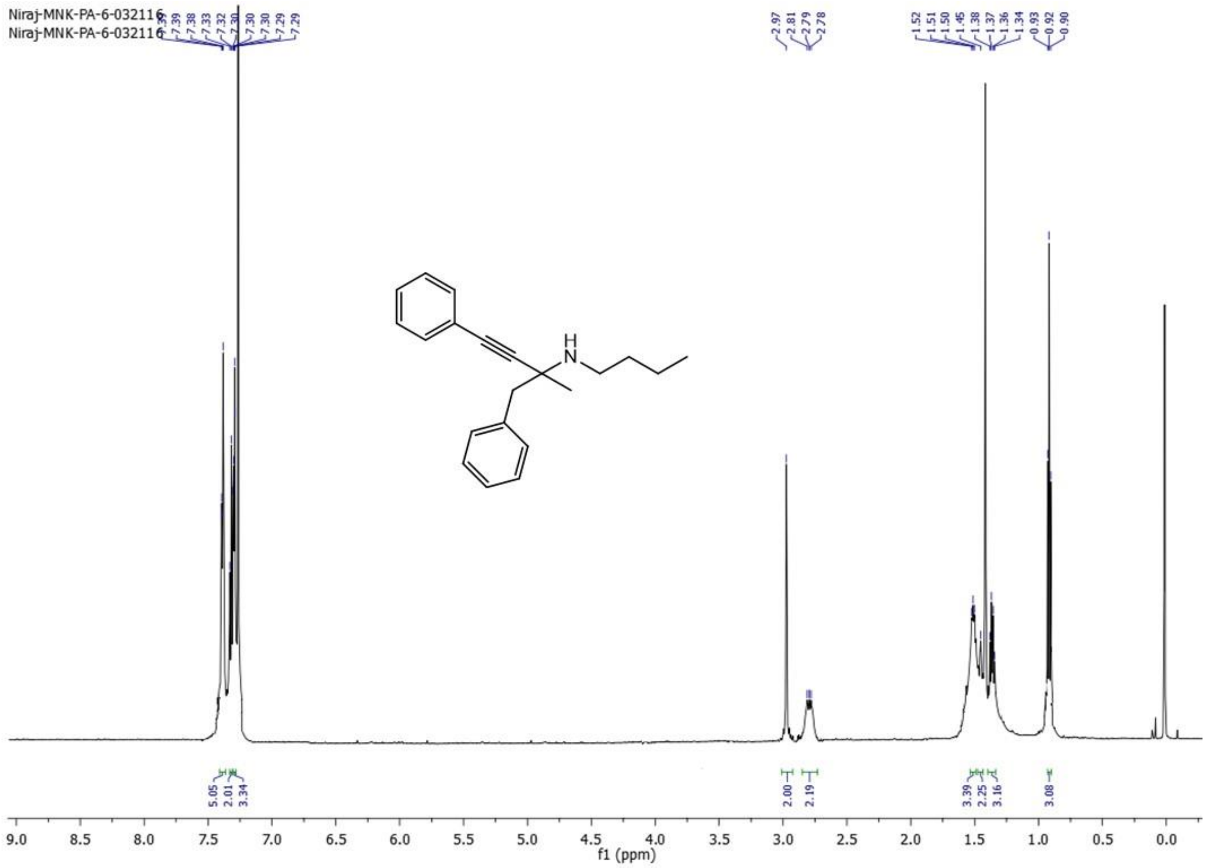

Supplementary Figure 26. ^{13}C NMR Spectra of butyl-(1-cyclohexyl-3-phenyl-prop-2-ynyl)-amine in $CDCl_3$.

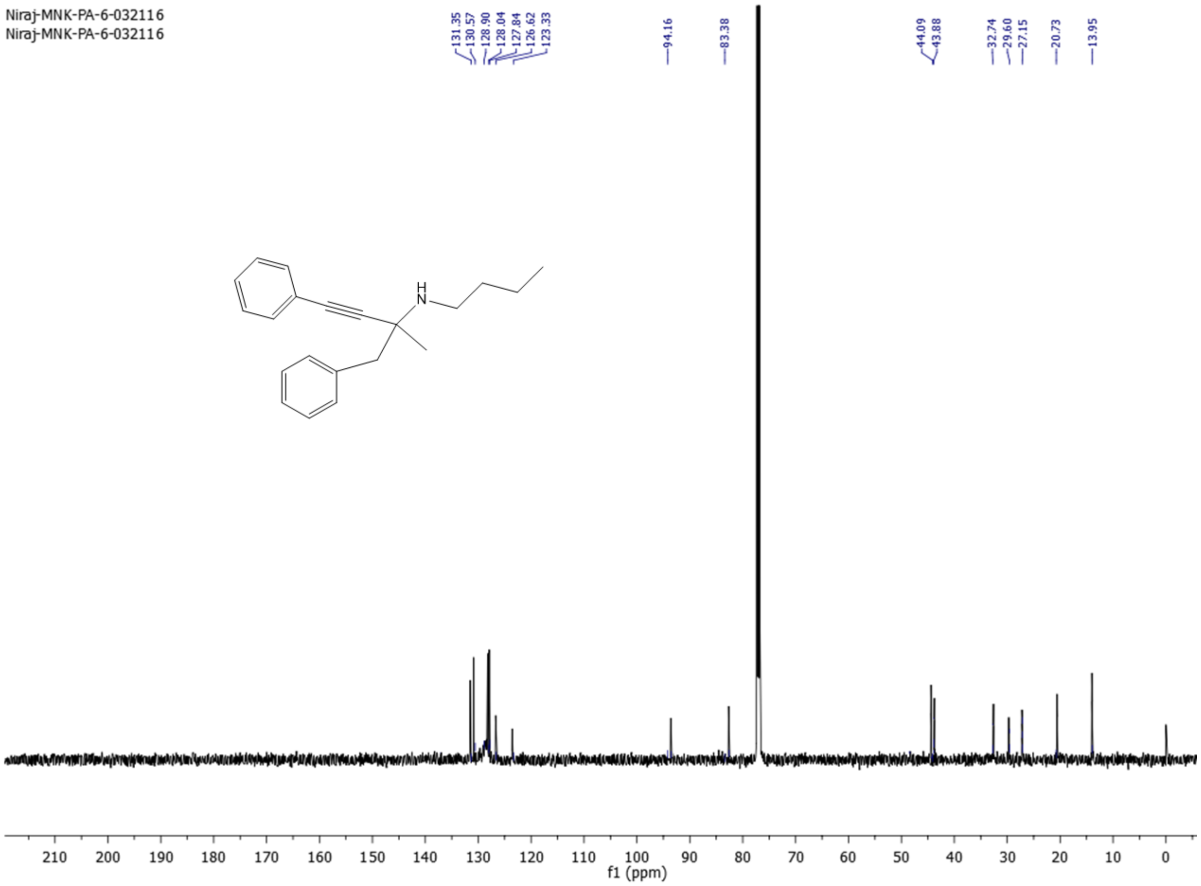

Supplementary Figure 27. ^{13}C NMR Spectra of *N*-butyl-2-methyl-1,4-diphenylbut-3-yn-2-amine in $CDCl_3$.

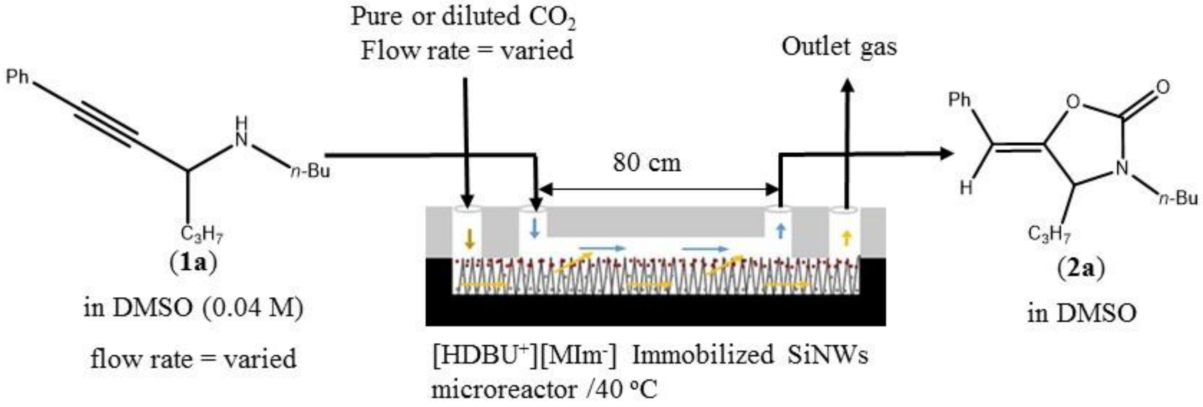

Supplementary Figure 28. Schematic diagram for optimization of interfacial catalytic synthesis of 5-benzylidene-3-butyl-4-propyl-oxazolidin-2-one (2a).

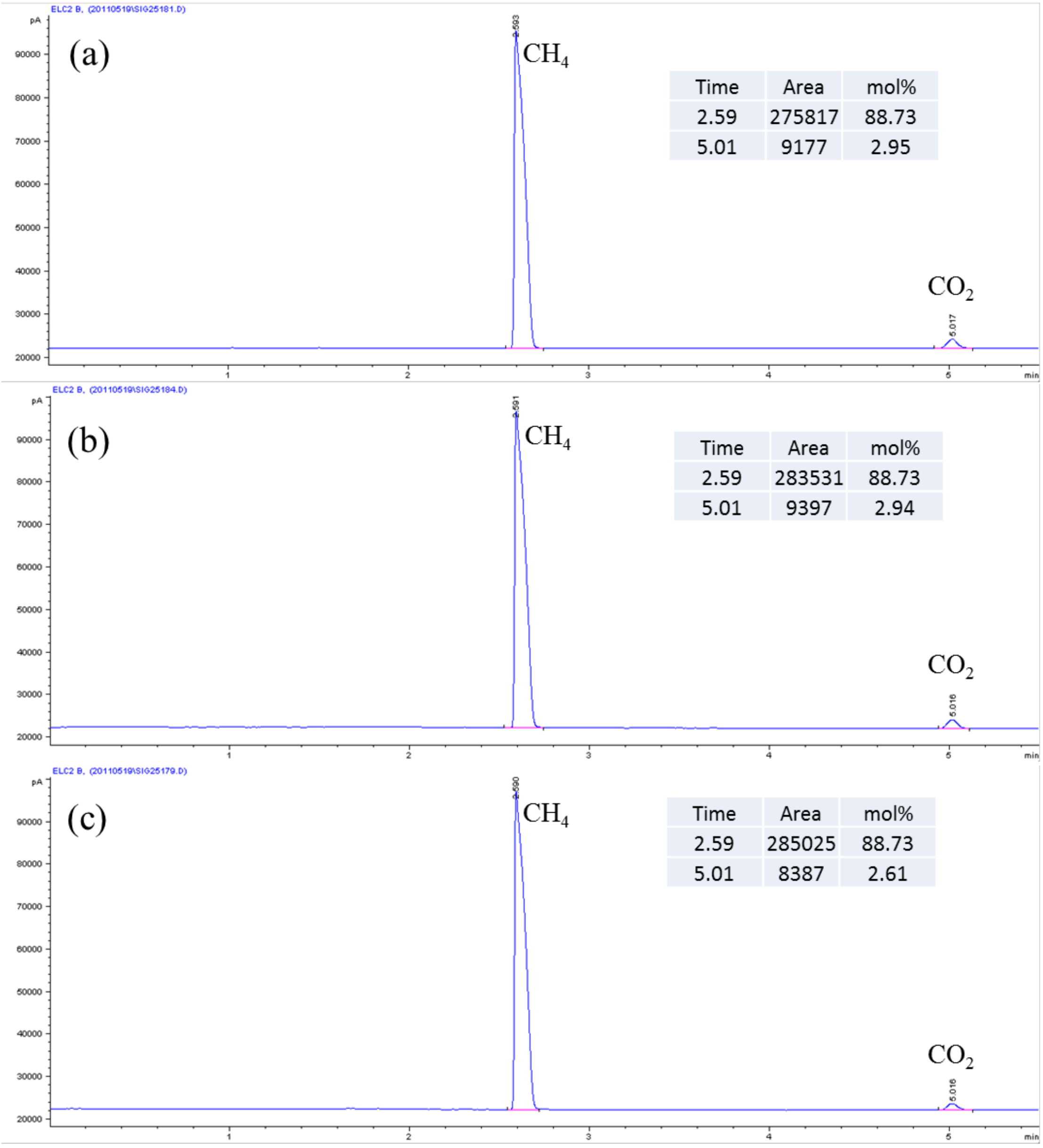
(a)
CH4
Time | Area | mol%
2.59 | 275817 | 88.73
5.01 | 9177 | 2.95
CO2
(b)
CH4
Time | Area | mol%
2.59 | 283531 | 88.73
5.01 | 9397 | 2.94
CO2
(c)
CH4
Time | Area | mol%
2.59 | 285025 | 88.73
5.01 | 8387 | 2.61
CO2

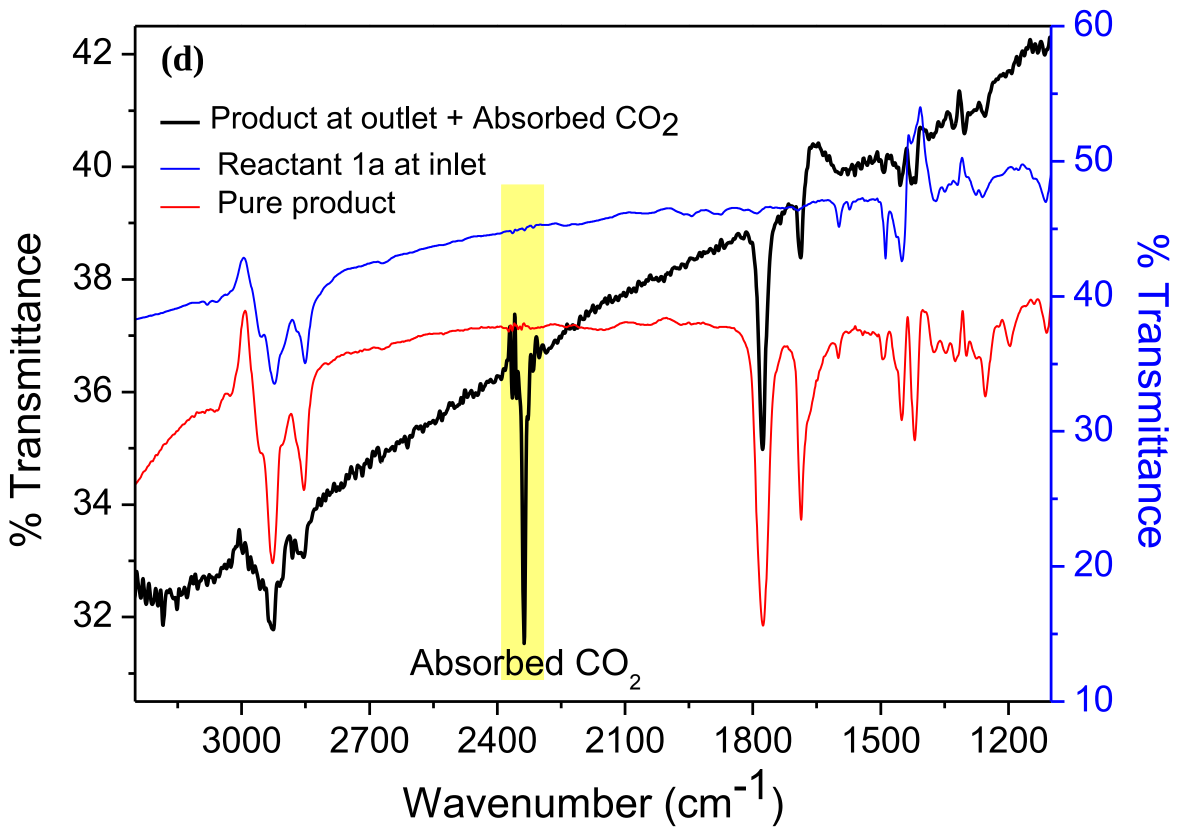

Supplementary Figure 29. Gas-Chromatogram of (a) natural gas inlet 2.95 mol% CO_2; (b) natural gas outlet of microreactor after interfacial contact with DMSO in absence of *N*-butyl-1-phenylhex-1-yn-3-amine (1a) 2.94 mol%; (c) natural gas sample collected from the outlet of microreactor after interfacial reaction with *N*-butyl-1-phenylhex-1-yn-3-amine (1a) in DMSO (2.61 mol%). Reaction condition: [1a] = 0.001 M, flow rate of 1a = 3 μL min^{-1}, flow rate of natural gas = 30 μL min^{-1}. Mole ratio CO_2/1a = (3.95 × 10^{-8}) /(0.3 × 10^{-8}) = 13.16; (d) FTIR spectra of absorbed CO_2 in product medium, Reaction condition: [1a] = 0.001 M, flow rate of 1a = 3 μL min^{-1}, flow rate of natural gas = 30 μL min^{-1}, at 40 ^{o}C and retention time 251 sec.

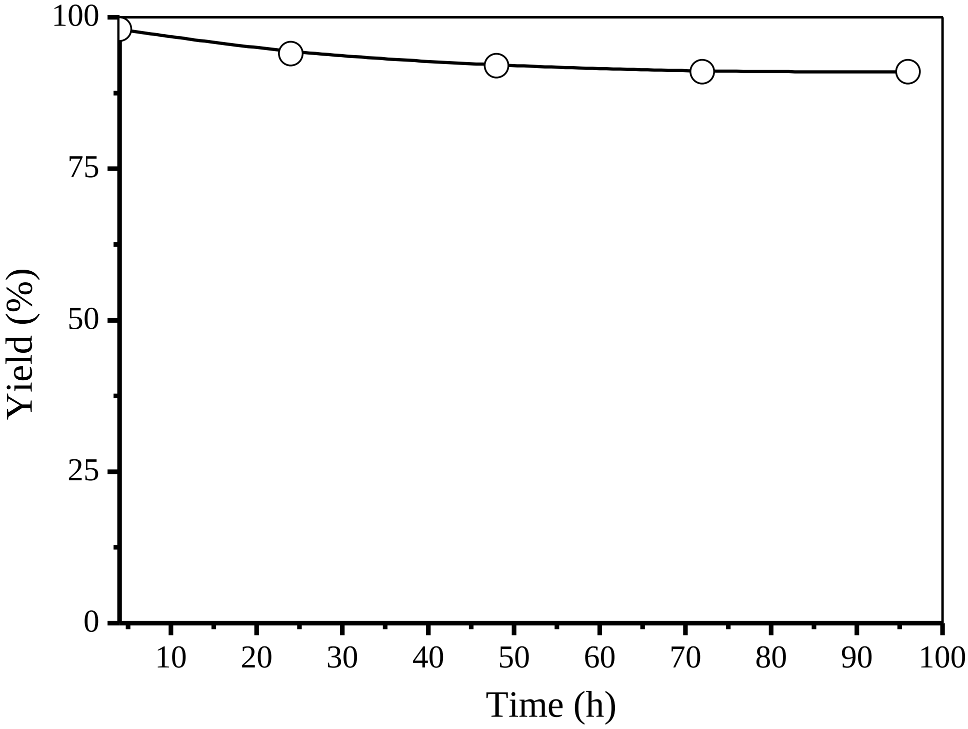

Supplementary Figure 30. Stability test of [HDBU$^+$][MIm$^-$] immobilized SiNWs microreactor for 4 days of continuous reaction under mole ratio CO_2/*N*-butyl-1-phenylhex-1-yn-3-amine=(1.34×10^{-6})/(0.16×10^{-6}) = 8.4. Yields are based on GC-MS.

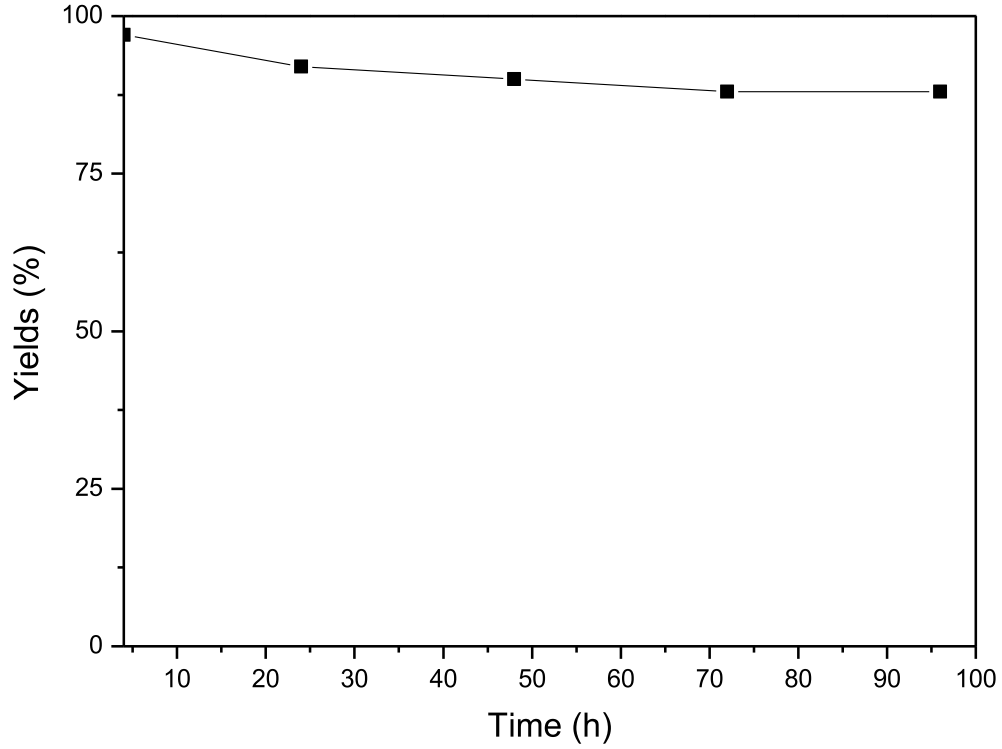

Supplementary Figure 31. Stability test of [HDBU$^+$][TFE$^-$] immobilized SiNWs microreactor for 4 days of continuous reaction under mole ratio CO_2/2-aminobenzonitrile=(1.56×10^{-6}) /(0.3×10^{-6}) = 5.2. Yields are based on GC-MS.

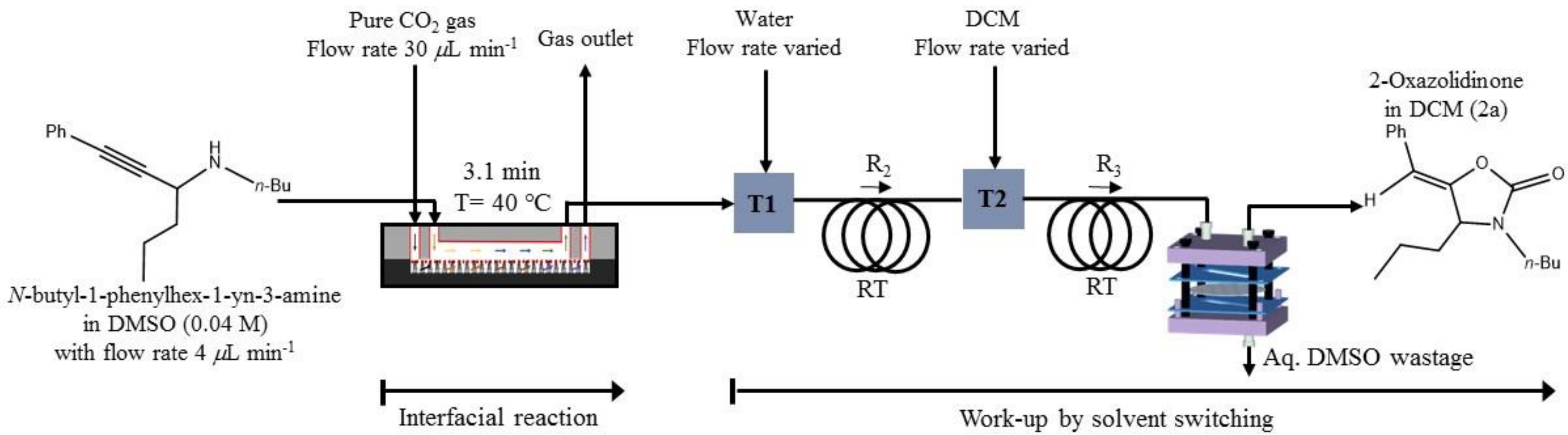

Supplementary Figure 32. Fully automated integrated system *via* interfacial catalysis reaction and work-up step with solvent switching (DMSO to DCM) for synthesis of 5-benzylidene-3-butyl-4-propyl-oxazolidin-2-one (2a).

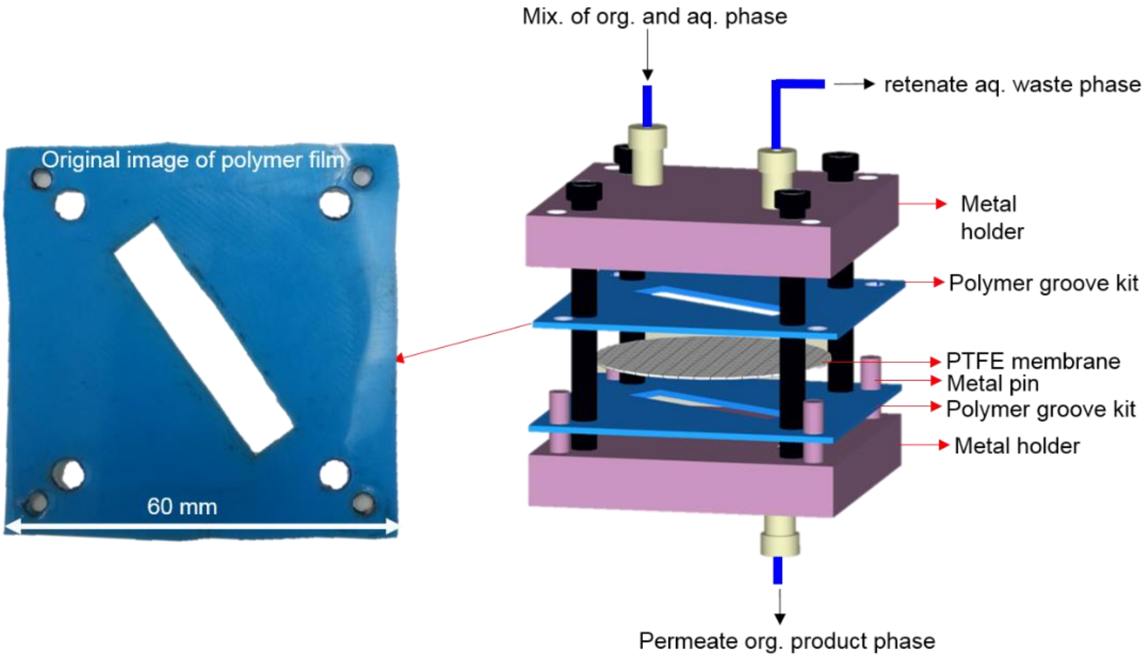

Supplementary Figure 33. Illustration of a fluoropolymer PTFE membrane microseparation sandwiched between two polyethylene films with punched channel.

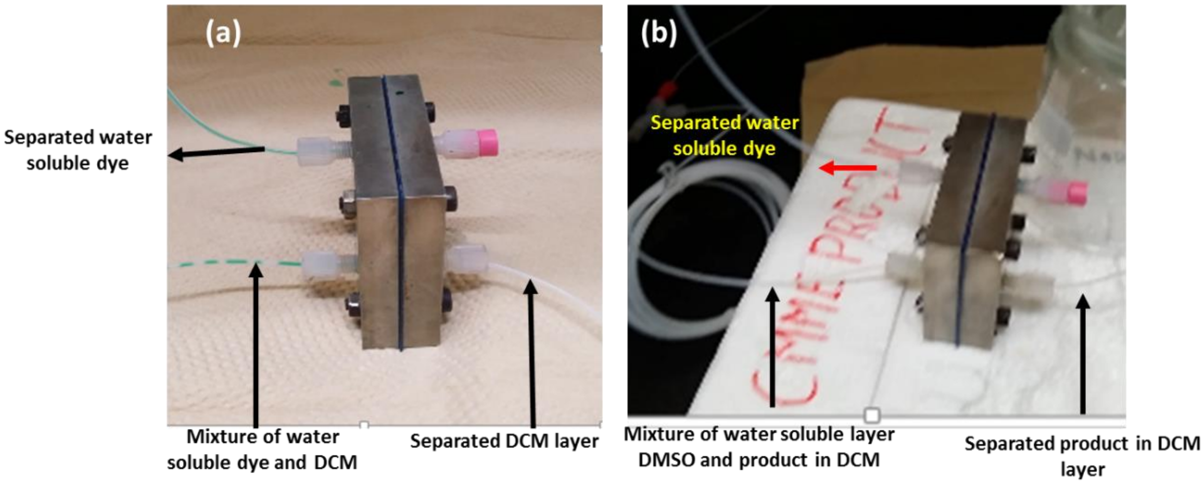

Supplementary Figure 34. Real time snapshot of the supplementary videos (Supplementary Movie 2, Movie 3) for microseparation.

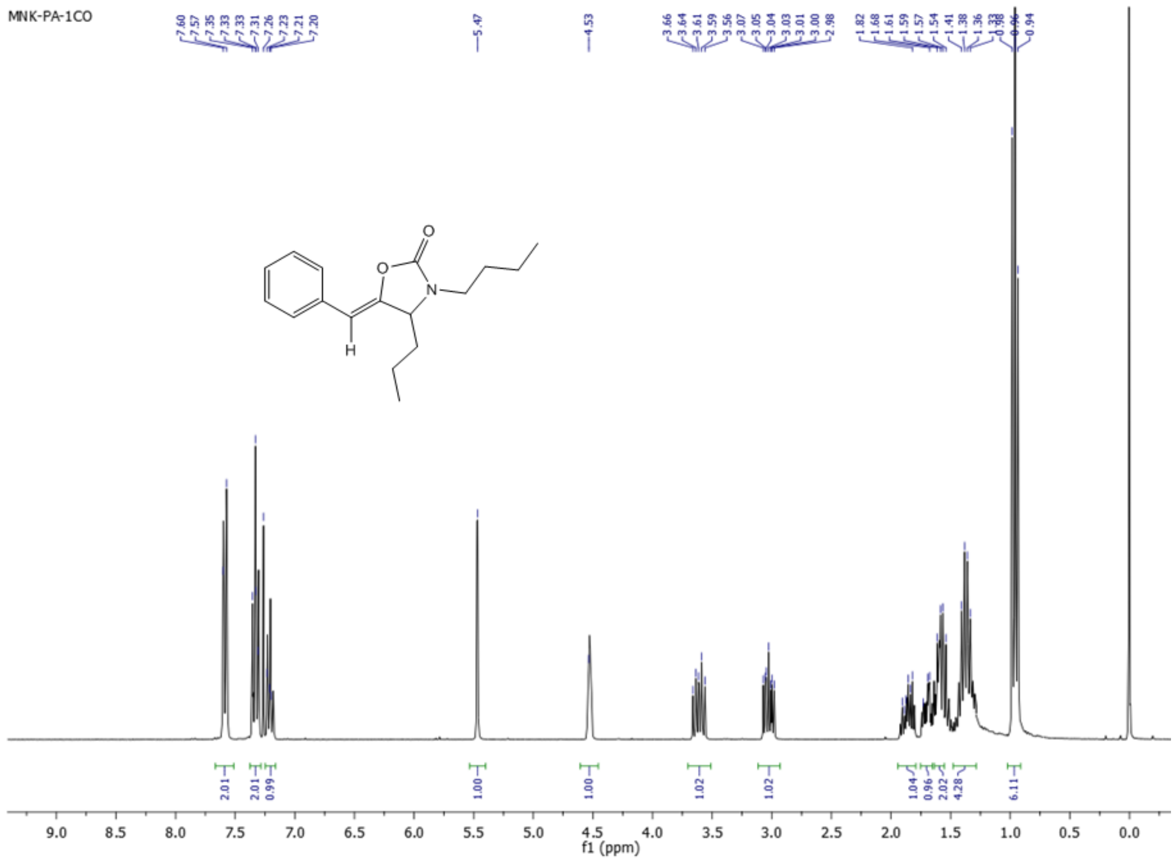

Supplementary Figure 35. ^{1}H NMR Spectra of 5-benzylidene-3-butyl-4-propyl-oxazolidin-2-one in $CDCl_3$.

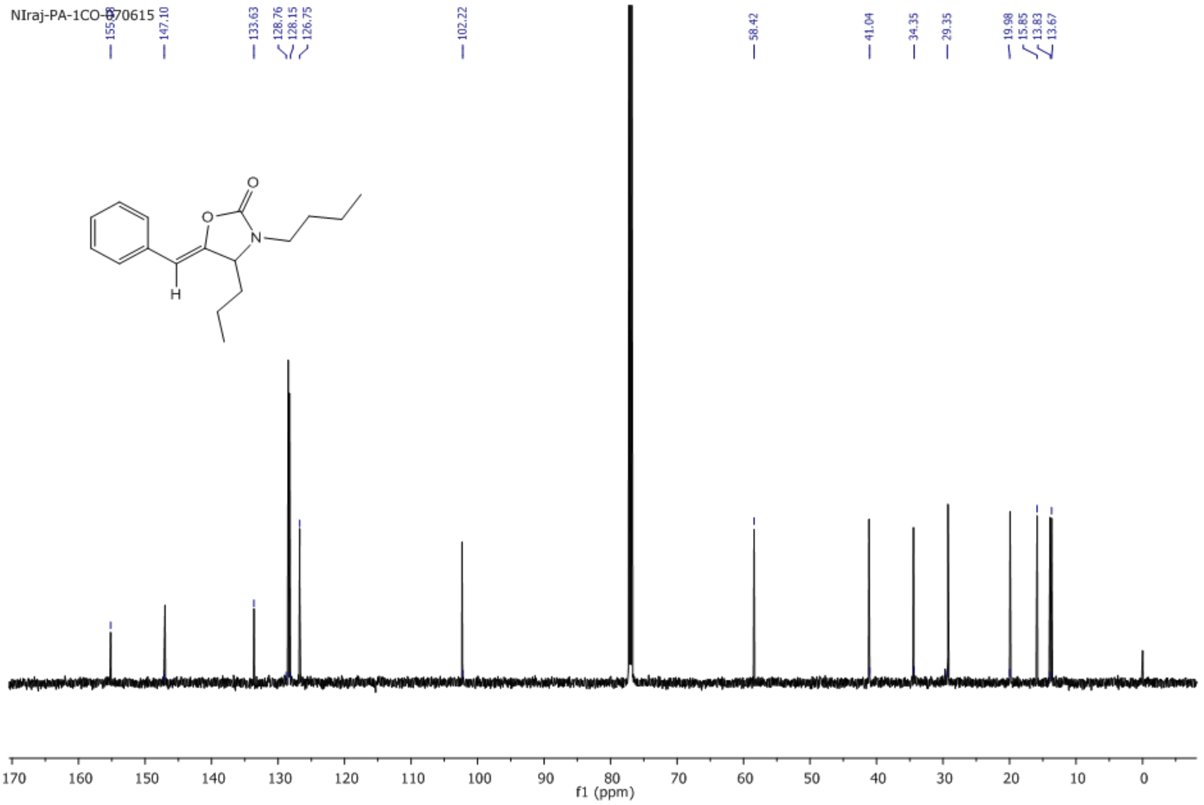

Supplementary Figure 36. ^{13}C NMR Spectra of 5-benzylidene-3-butyl-4-propyl-oxazolidin-2-one in $CDCl_3$.

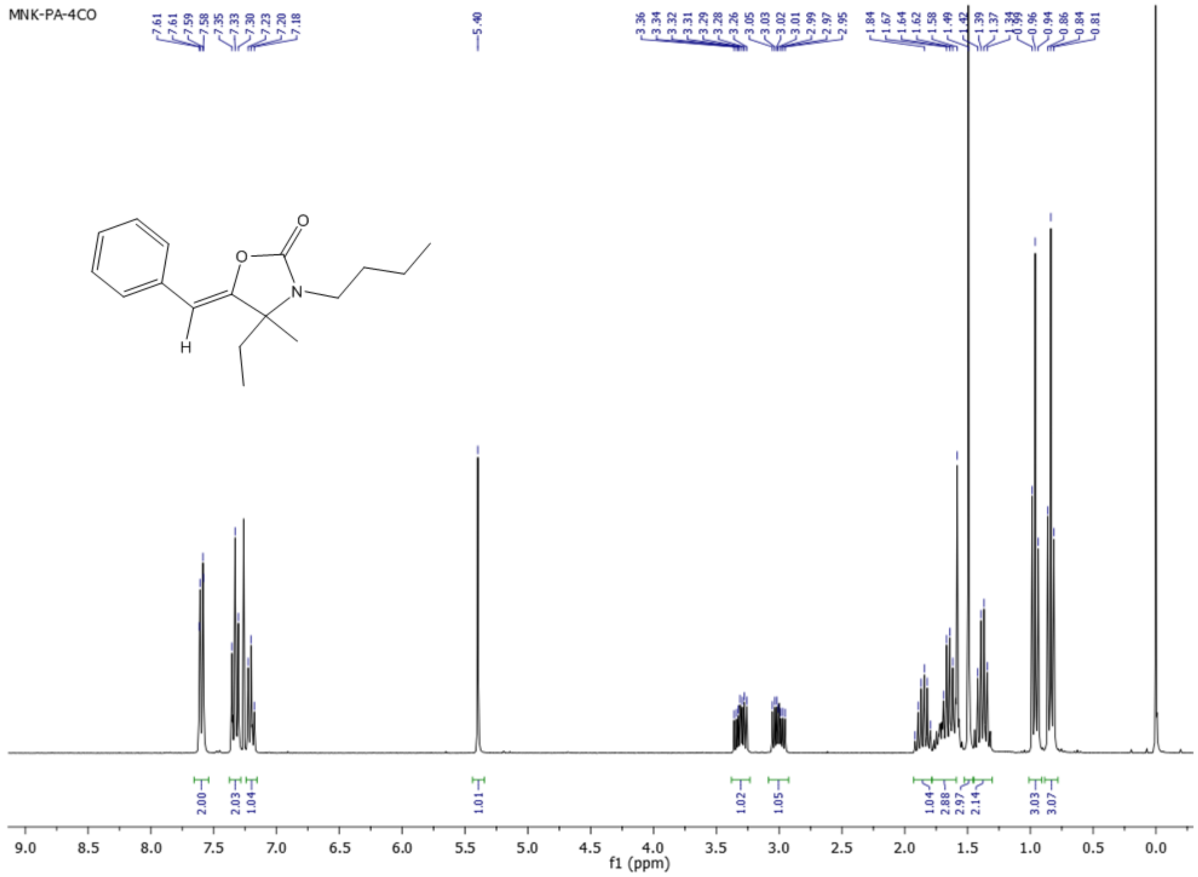

Supplementary Figure 37. ^{1}H NMR Spectra of 5-benzylidene-3-butyl-4-methyl-4-ethyl-oxazolidin-2-one in $CDCl_3$.

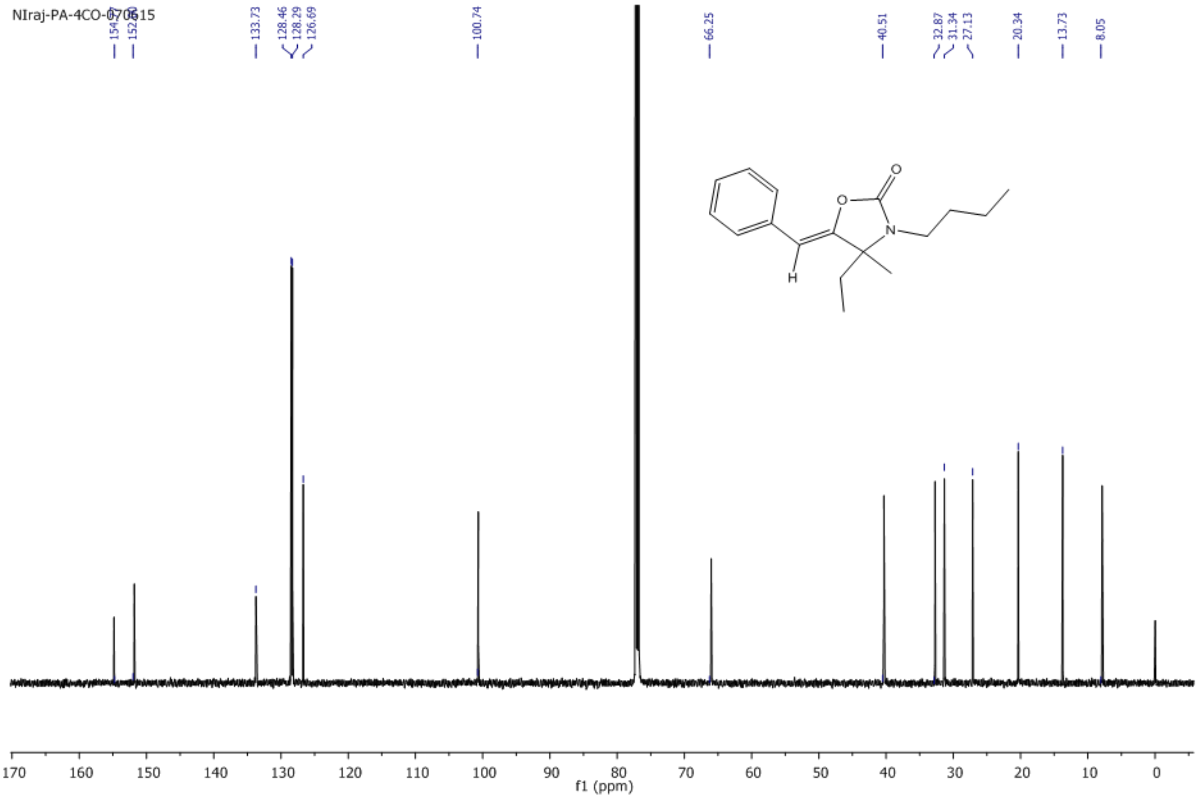

Supplementary Figure 38. ^{13}C NMR Spectra of 5-benzylidene-3-butyl-4-methyl-4-ethyl-oxazolidin-2-one in $CDCl_3$.

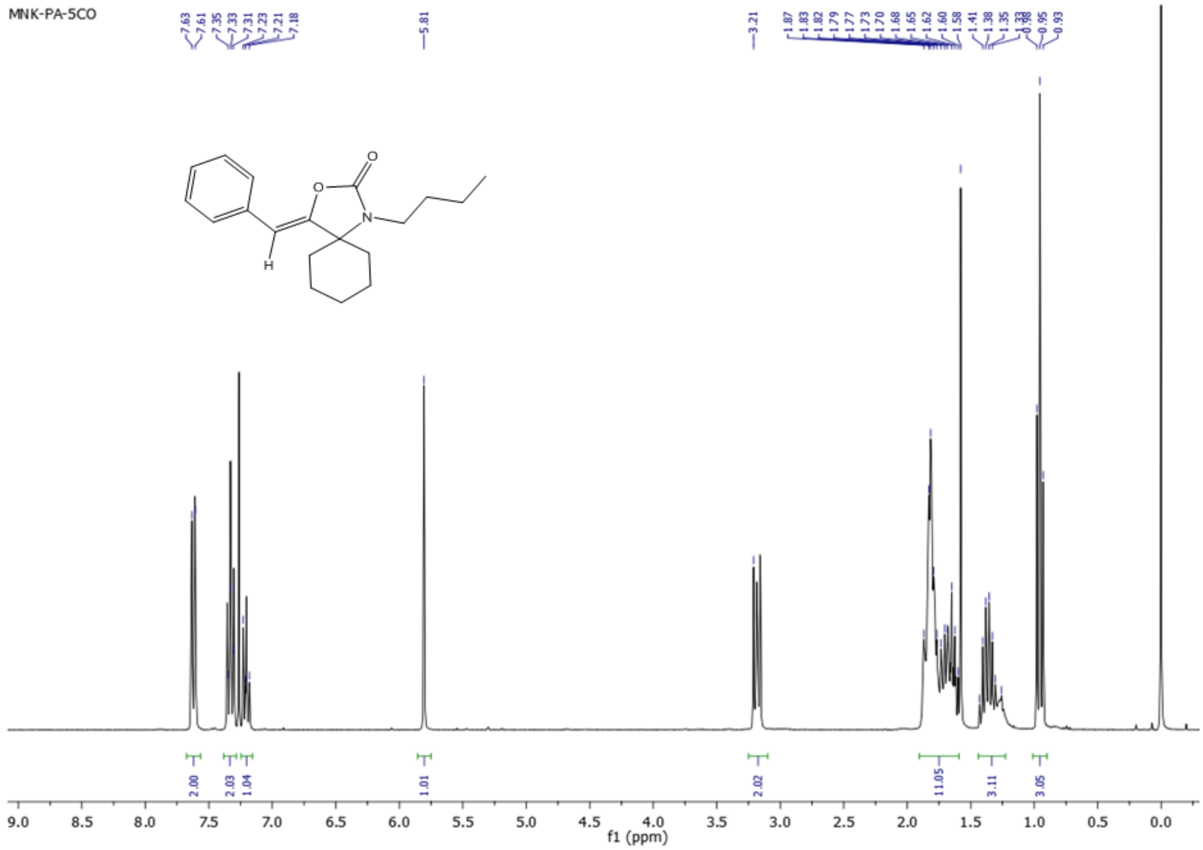

Supplementary Figure 39. ^{1}H NMR Spectra of 4-benzylidene-1-butyl-3-oxa-1-aza-spiro[4.5]decan-2-one in $CDCl_3$.

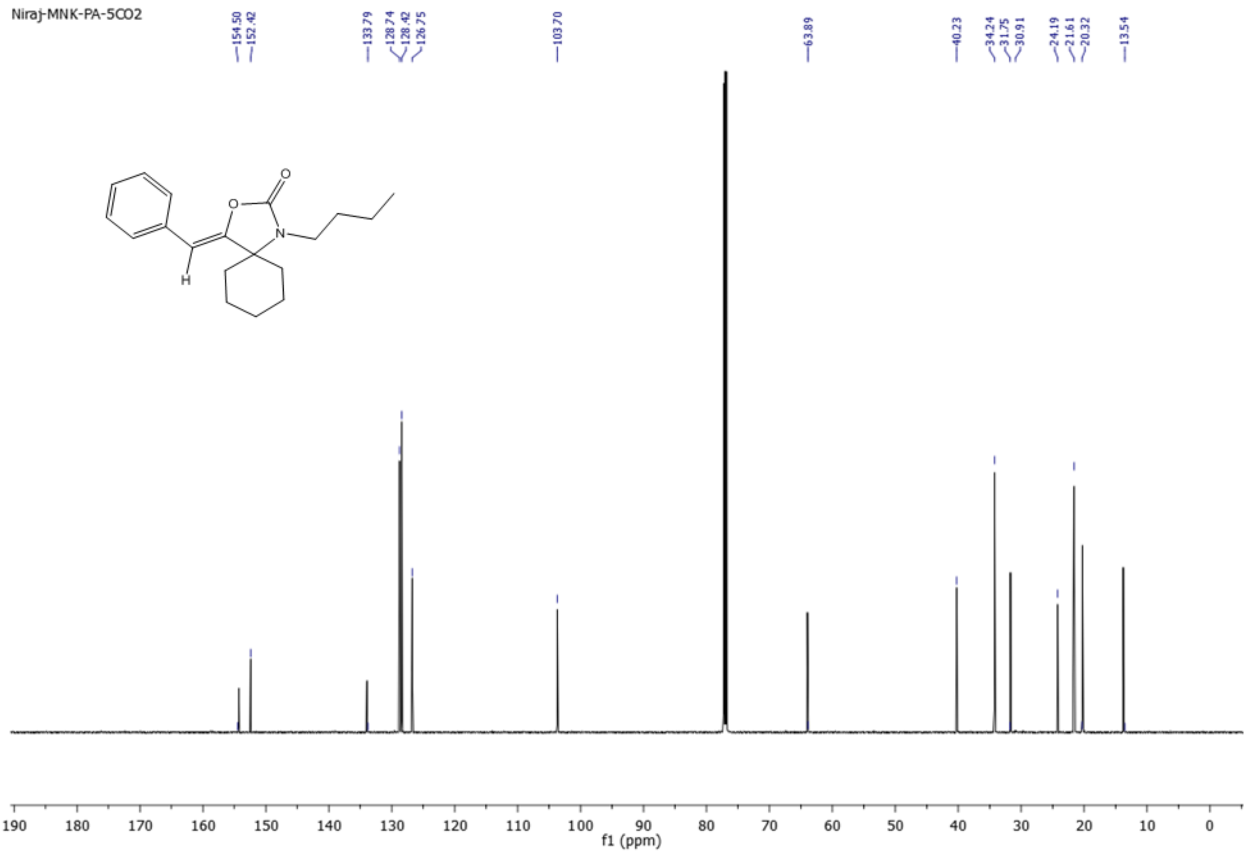

Supplementary Figure 40. ^{13}C NMR Spectra of 4-benzylidene-1-butyl-3-oxa-1-aza-spiro[4.5]decan-2-one in $CDCl_3$.

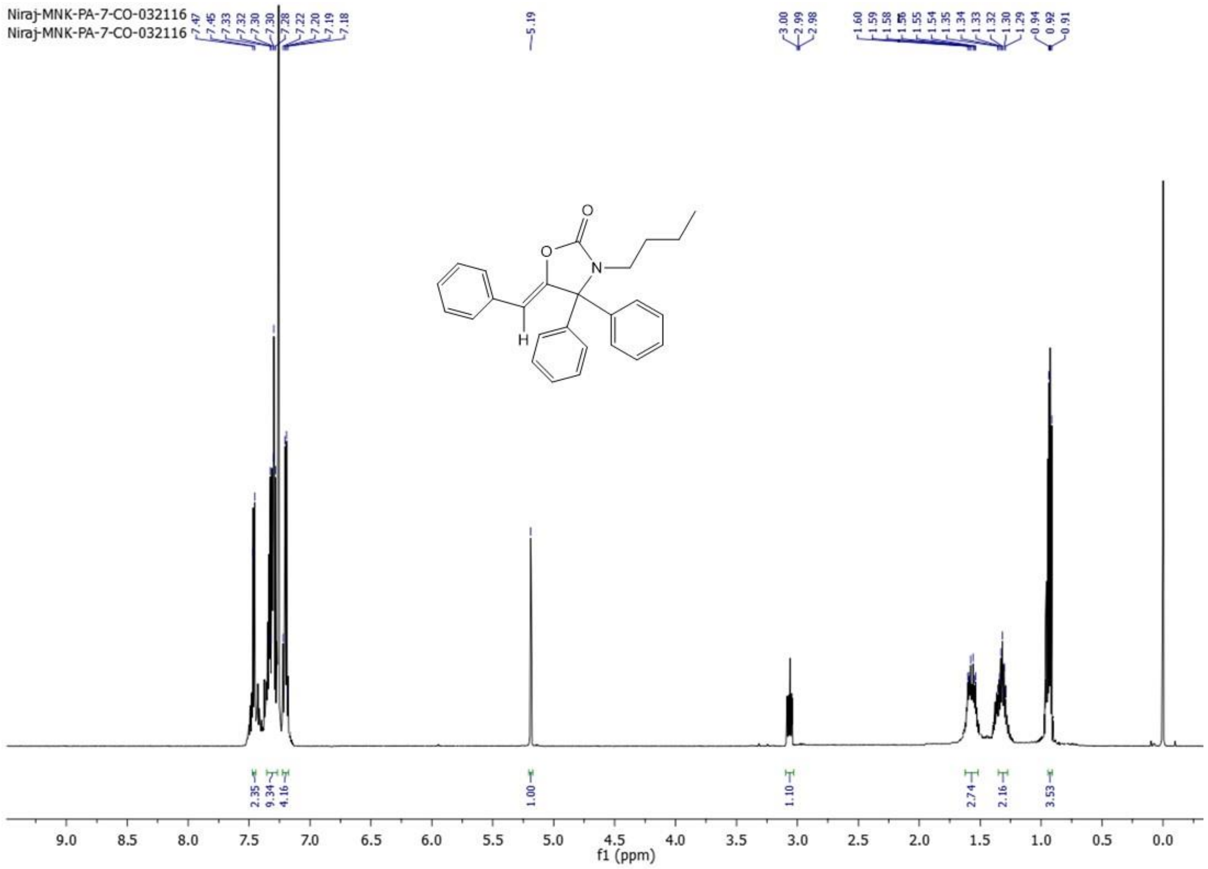

Supplementary Figure 41. ^{13}C NMR Spectra of 5-benzylidene-3-butyl-4,4-diphenyloxazolidin-2-one in $CDCl_3$.

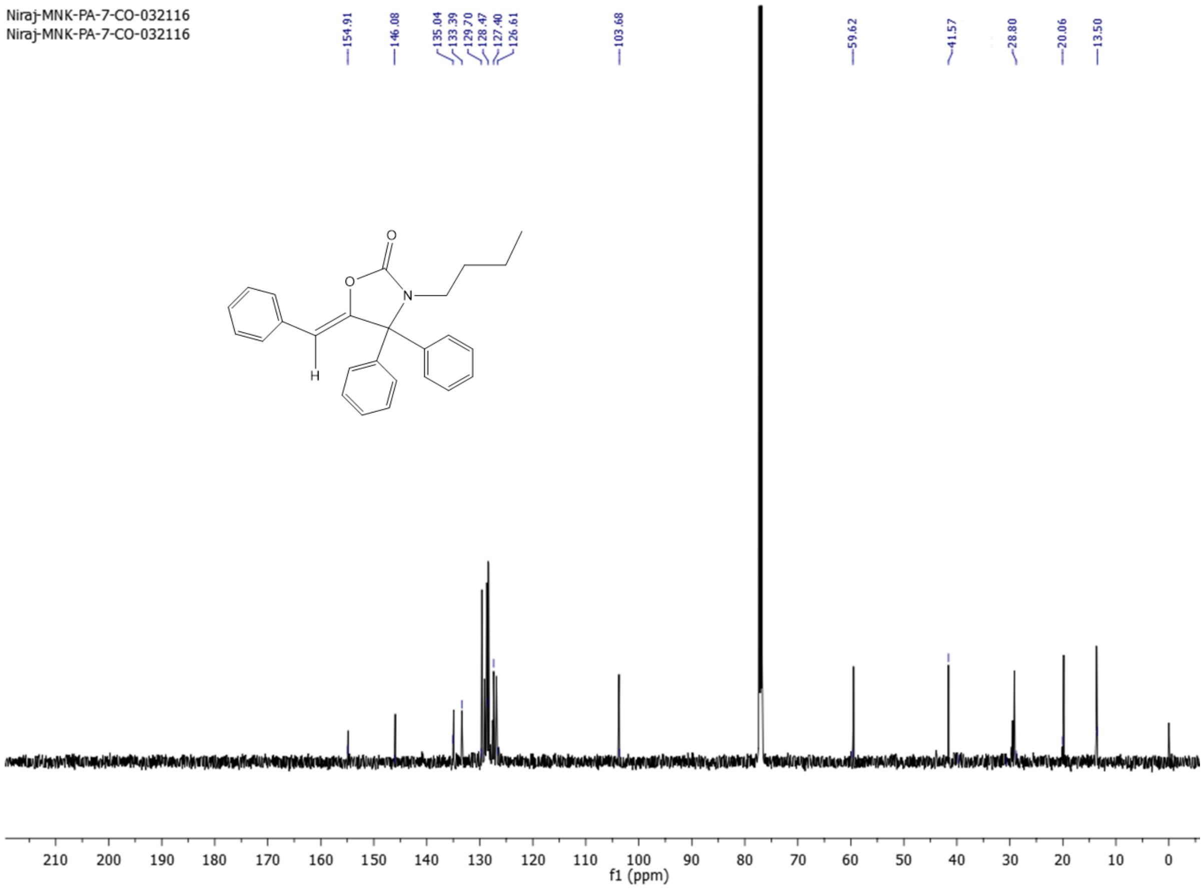

Supplementary Figure 42. ^{13}C NMR Spectra of 5-benzylidene-3-butyl-4,4-diphenyloxazolidin-2-one in $CDCl_3$.

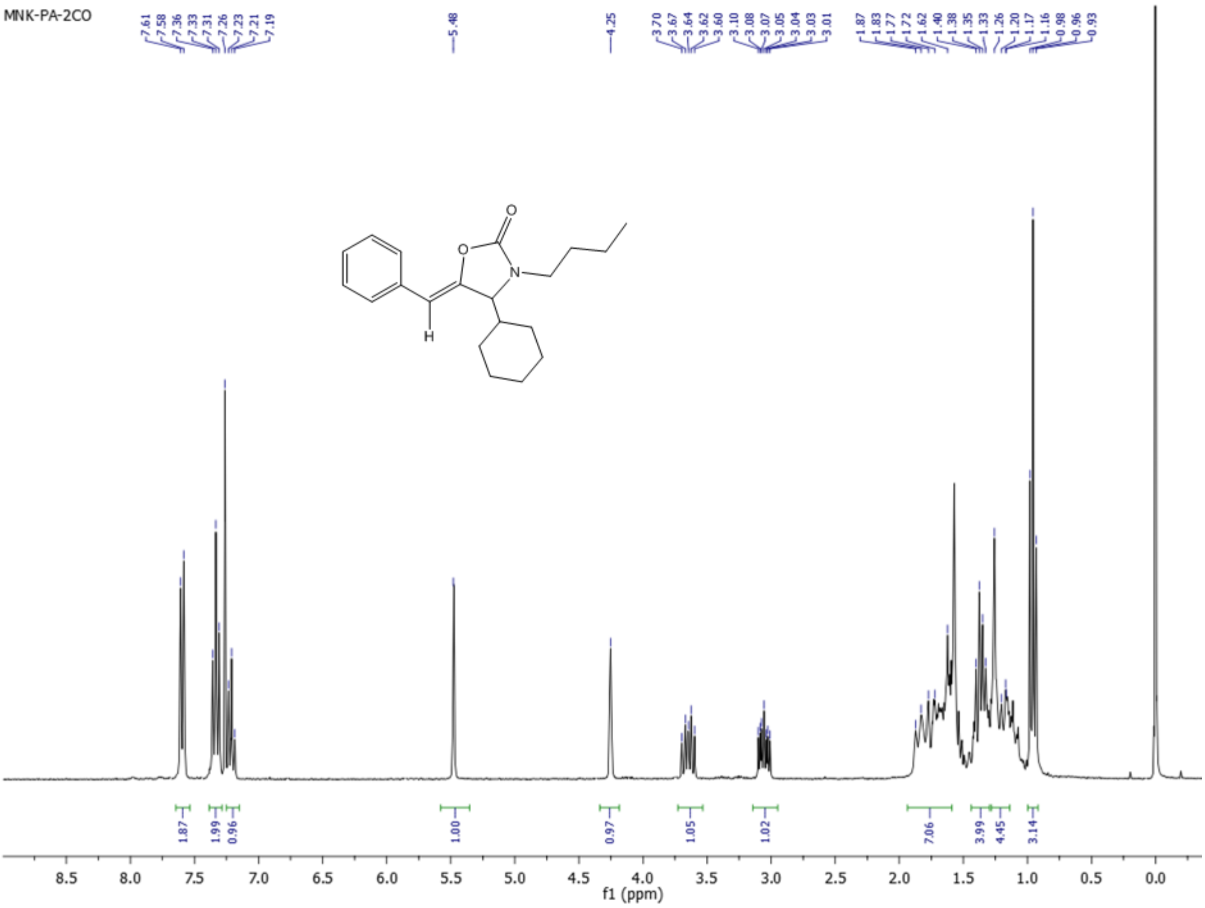

Supplementary Figure 43. ^{1}H NMR Spectra of 5-benzylidene-3-butyl-4-cyclohexyl-oxazolidin-2-one in $CDCl_3$.

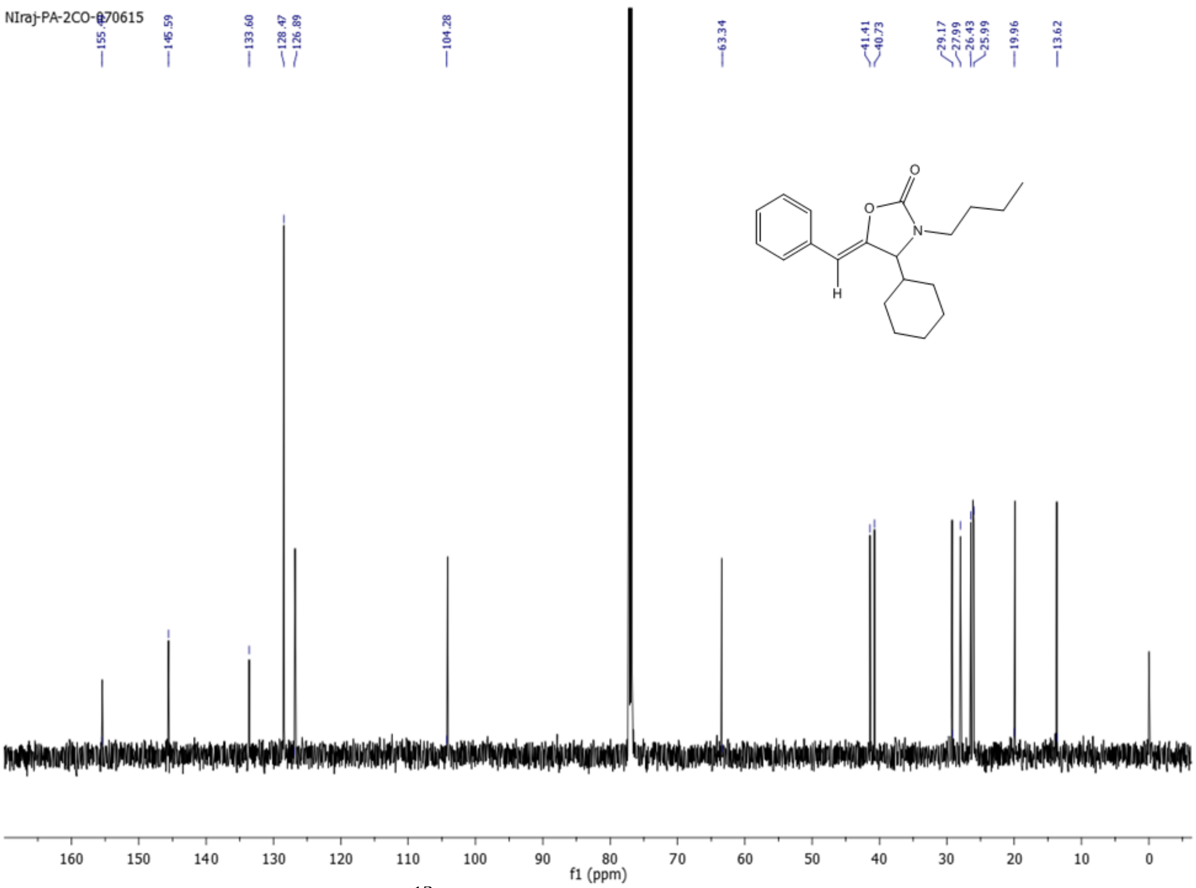

Supplementary Figure 44. ^{13}C NMR Spectra of 5-benzylidene-3-butyl-4-cyclohexyl-oxazolidin-2-one in $CDCl_3$.

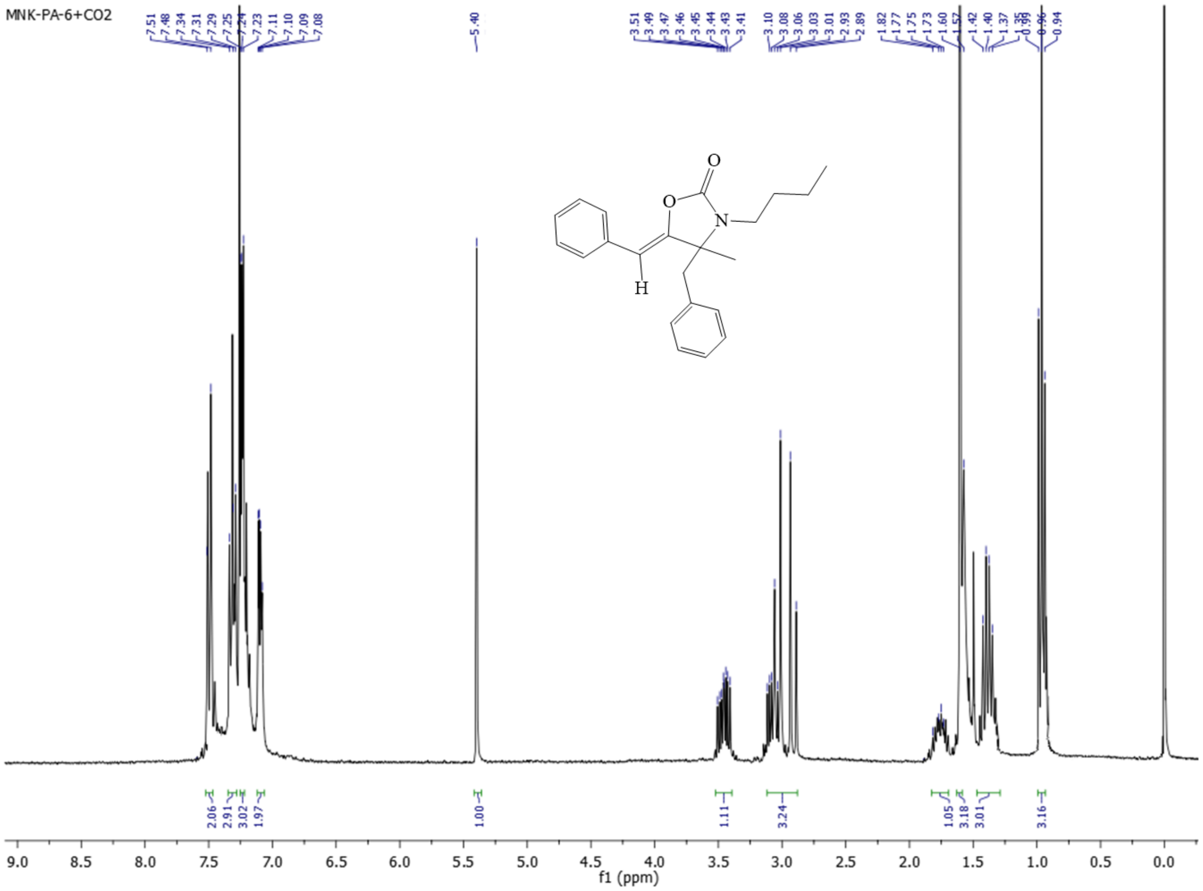

Supplementary Figure 45. ^{1}H NMR Spectra of 4-benzyl-5-benzylidene-3-butyl-4-methyloxazolidin-2-one in $CDCl_3$.

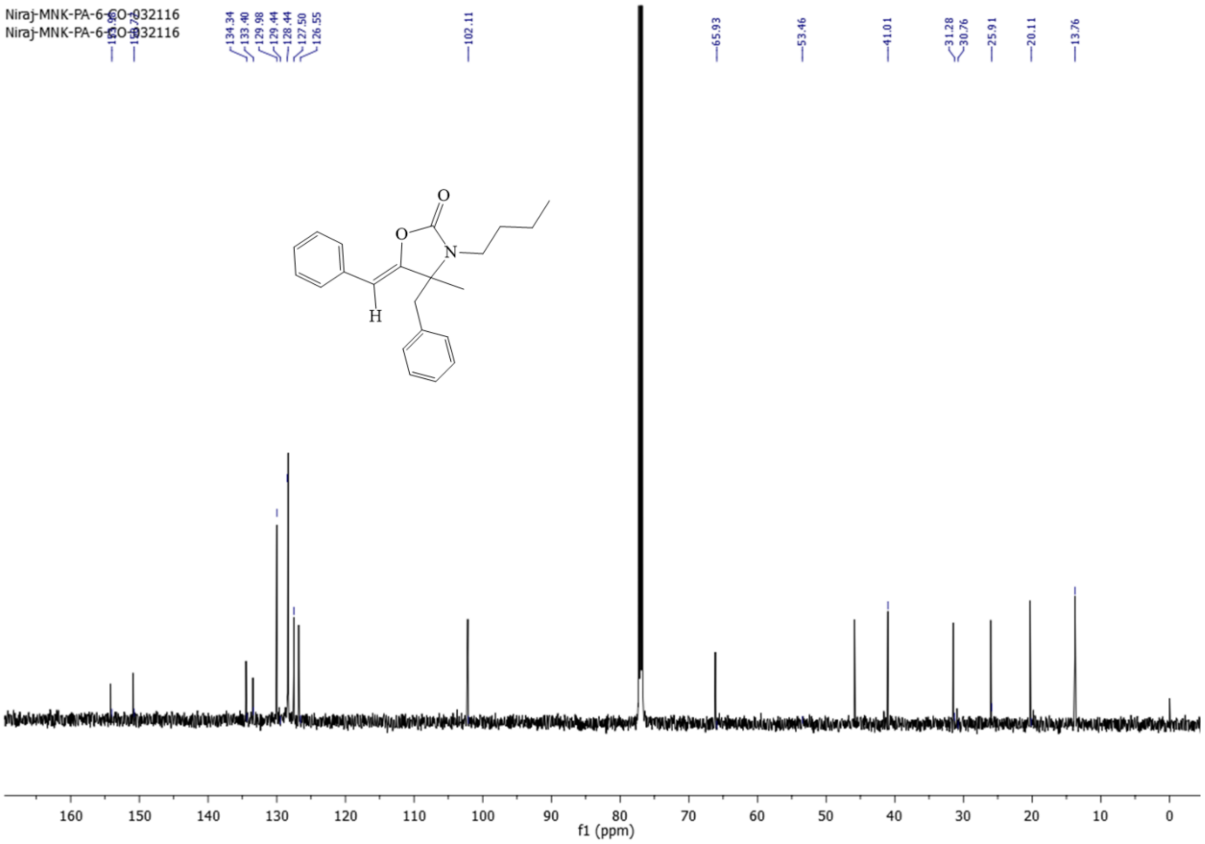

Supplementary Figure 46. ^{13}C NMR Spectra of 4-benzyl-5-benzylidene-3-butyl-4-methyloxazolidin-2-one in $CDCl_3$.

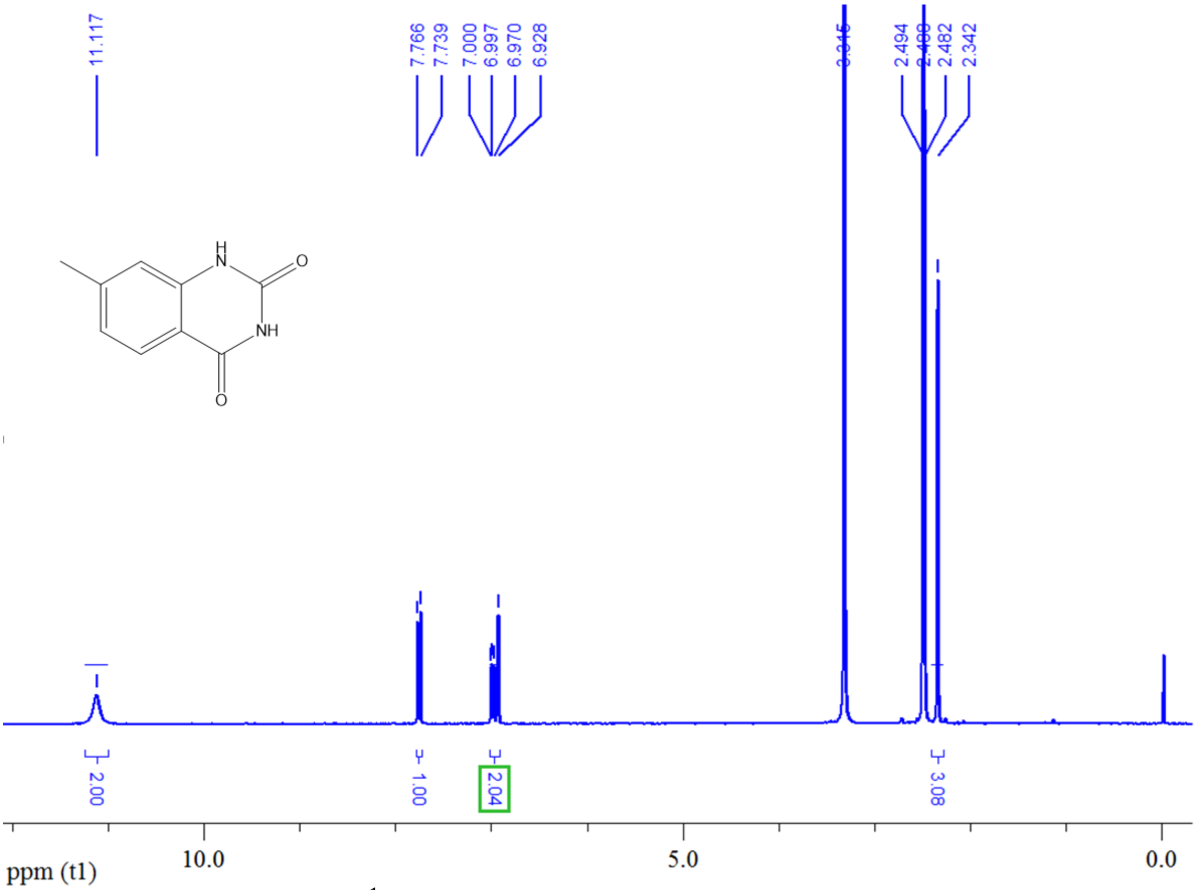

Supplementary Figure 47. ^{1}H NMR Spectra of 7-methylquinazoline-2,4(1*H*,3*H*)-dione in DMSO-d_6.

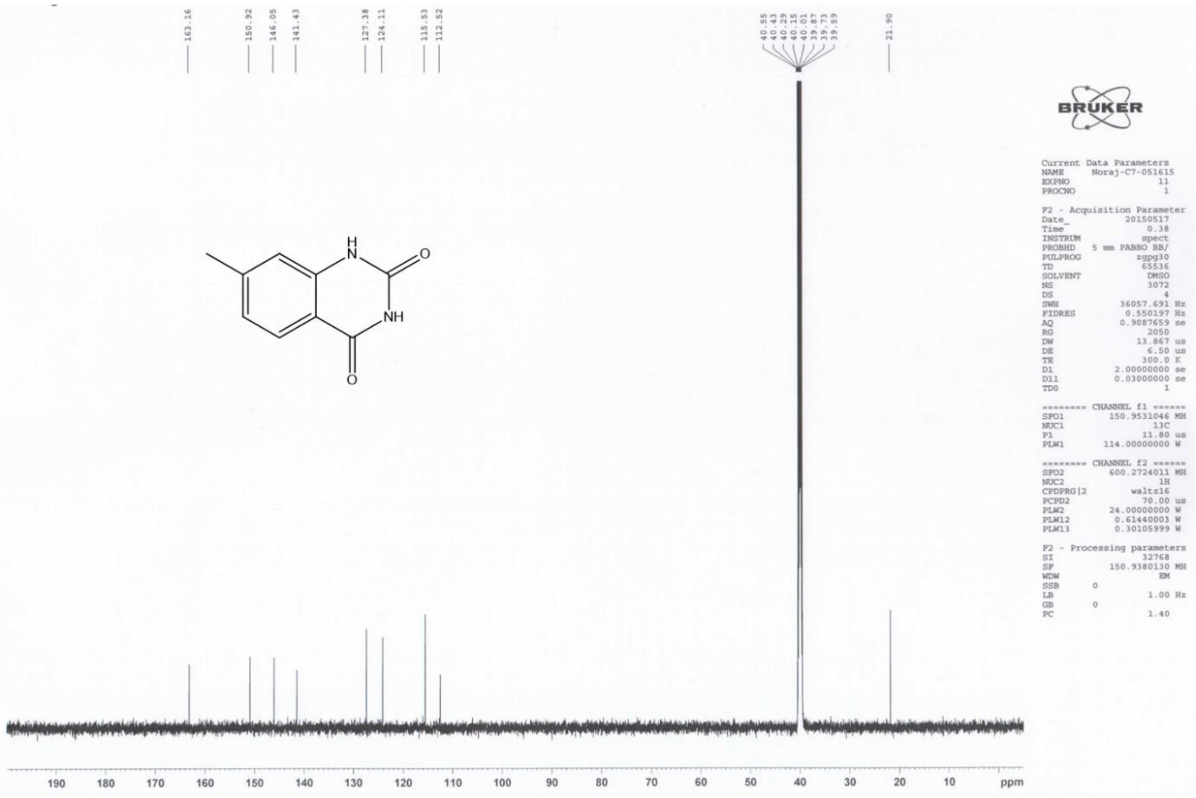

Supplementary Figure 48. ^{13}C NMR Spectra of 7-methylquinazoline-2,4(1*H*,3*H*)-dione in DMSO-d_6.

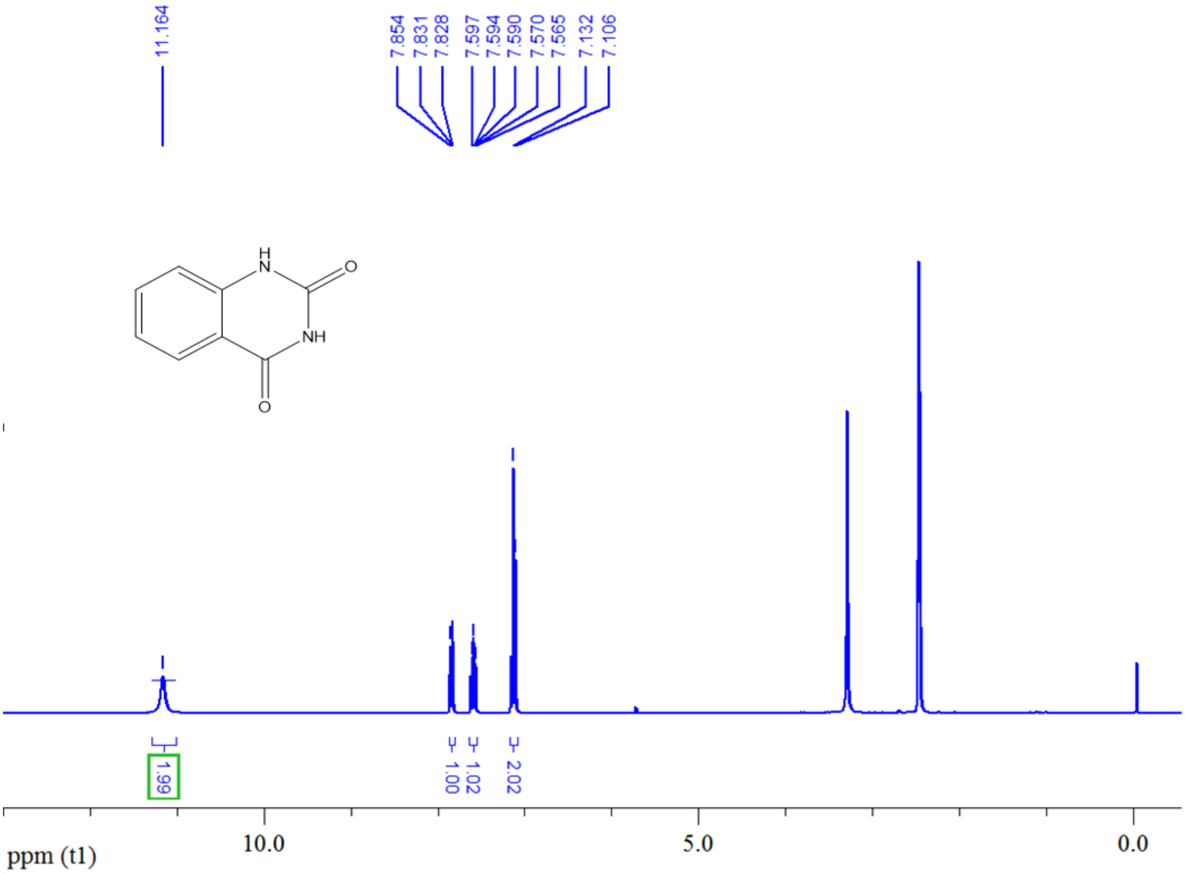

Supplementary Figure 49. ^{1}H NMR Spectra of quinazoline-2,4(1*H*,3*H*)-dione in DMSO-d_6.

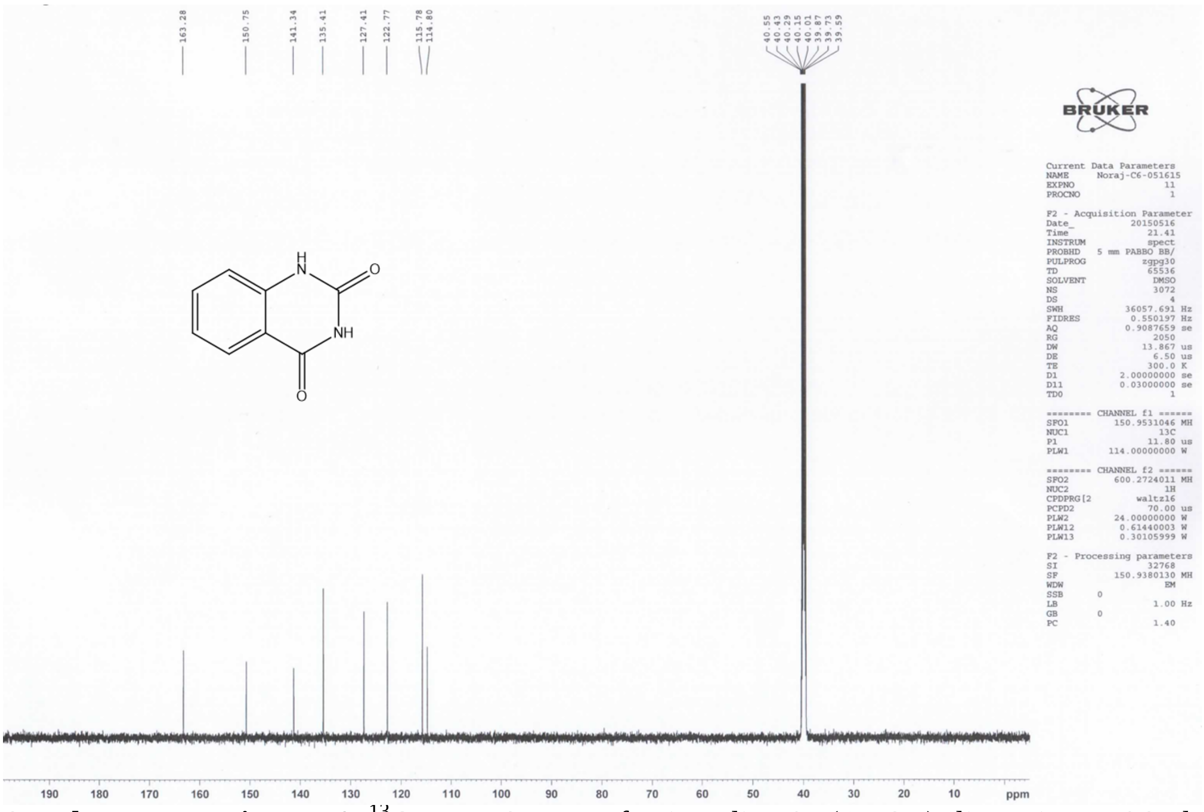

Supplementary Figure 50. ^{13}C NMR Spectra of quinazoline-2,4(1*H*,3*H*)-dione in DMSO-d_6.

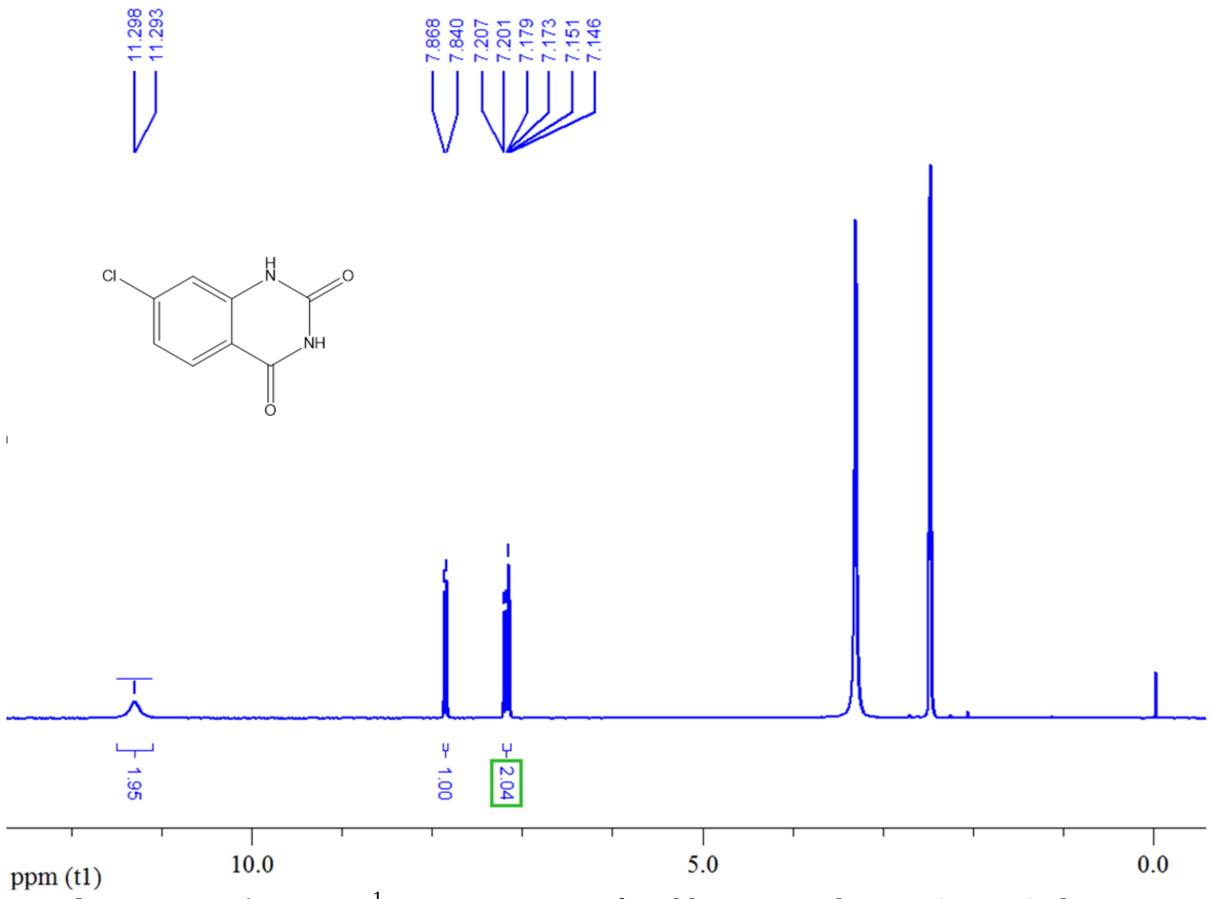

Supplementary Figure 51. ^{1}H NMR Spectra of 7-chloroquinazoline-2,4(1*H*,3*H*)-dione in DMSO-d_6.

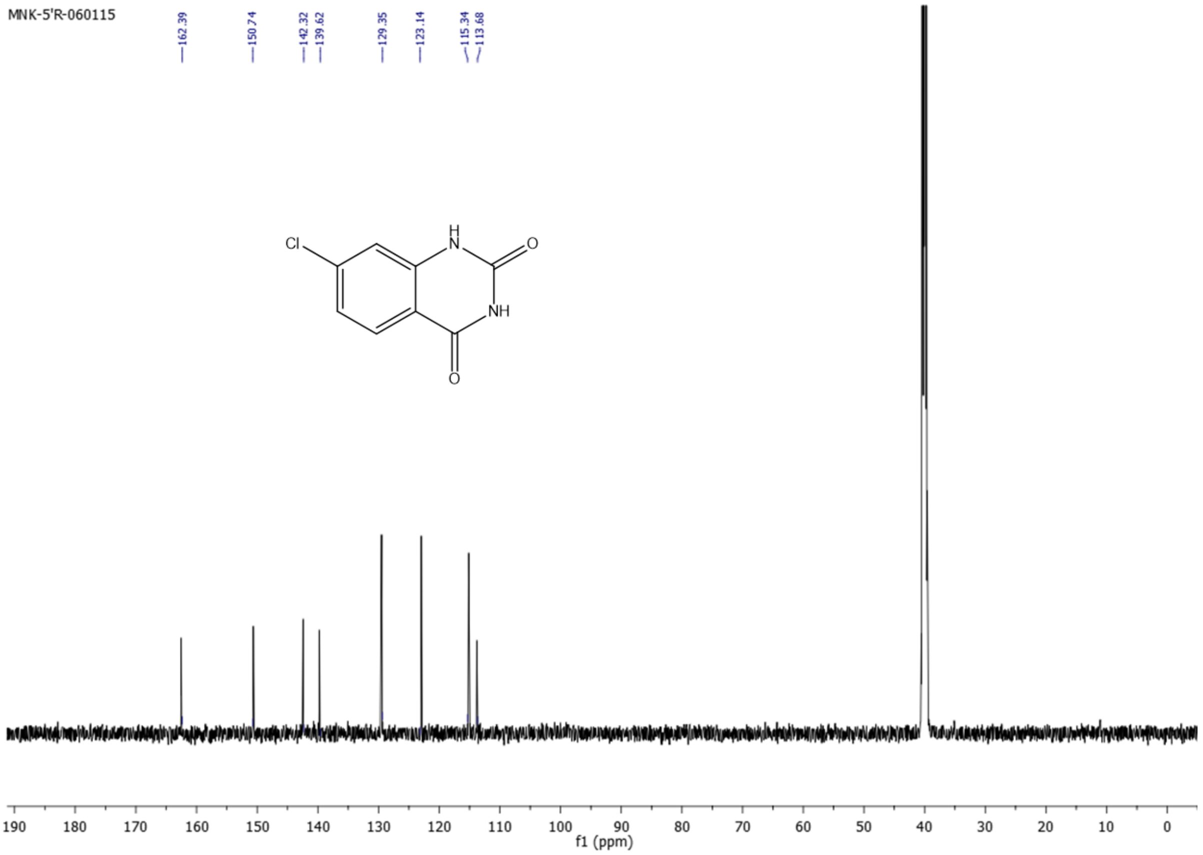

Supplementary Figure 52. ^{13}C NMR Spectra of 7-chloroquinazoline-2,4(1*H*,3*H*)-dione in DMSO-d_6.

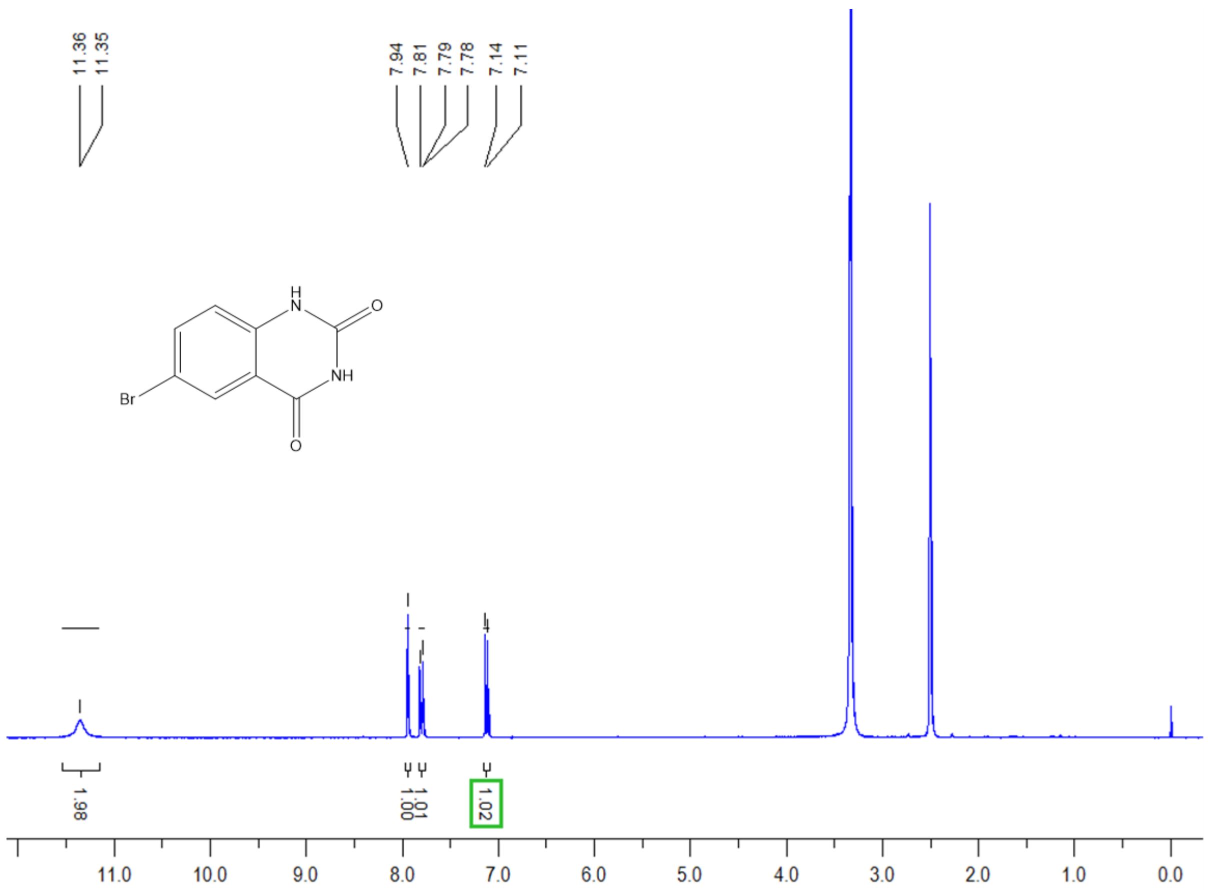

Supplementary Figure 53. ^{1}H NMR Spectra of 6-bromoquinazoline-2,4(1*H*,3*H*)-dione in DMSO-d_6.

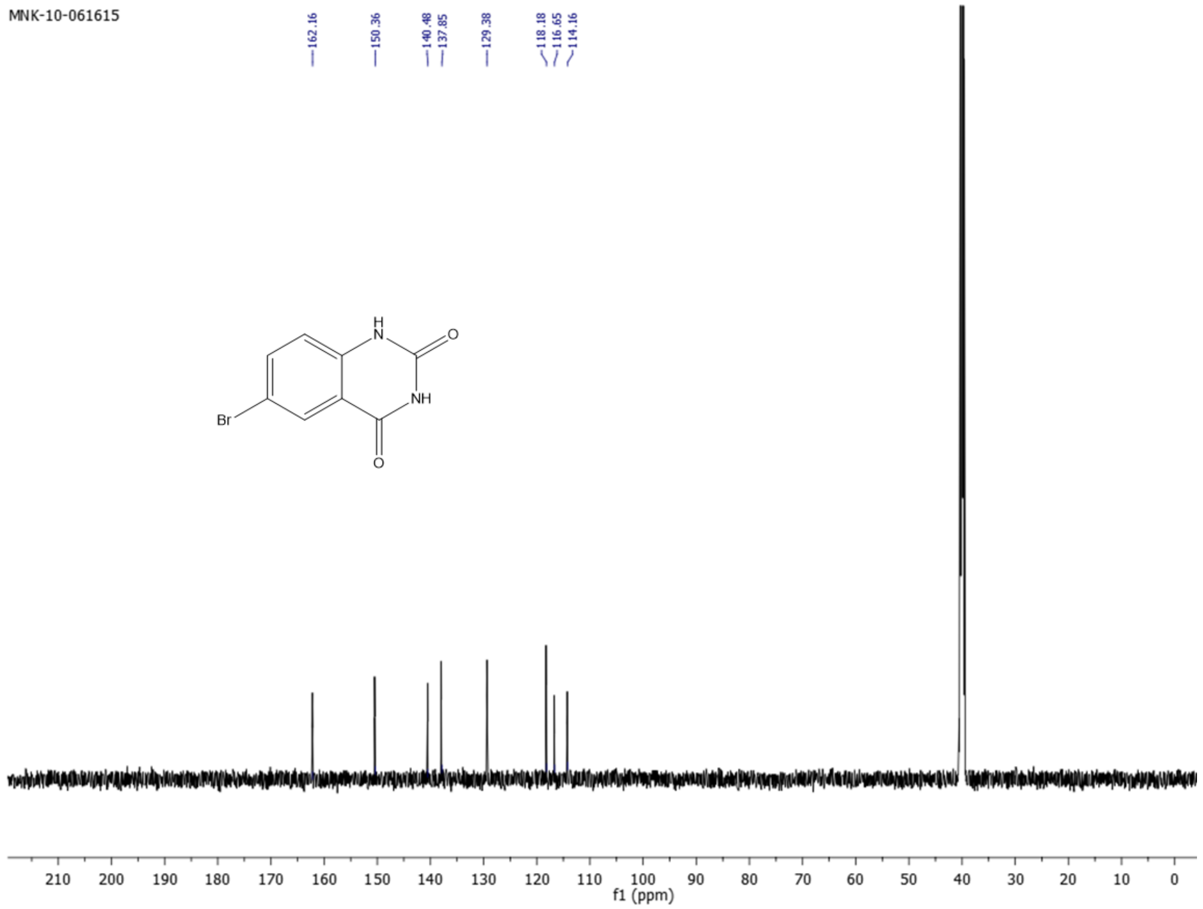

Supplementary Figure 54. ^{13}C NMR Spectra of 6-bromoquinazoline-2,4(1*H*,3*H*)-dione in DMSO-d_6.

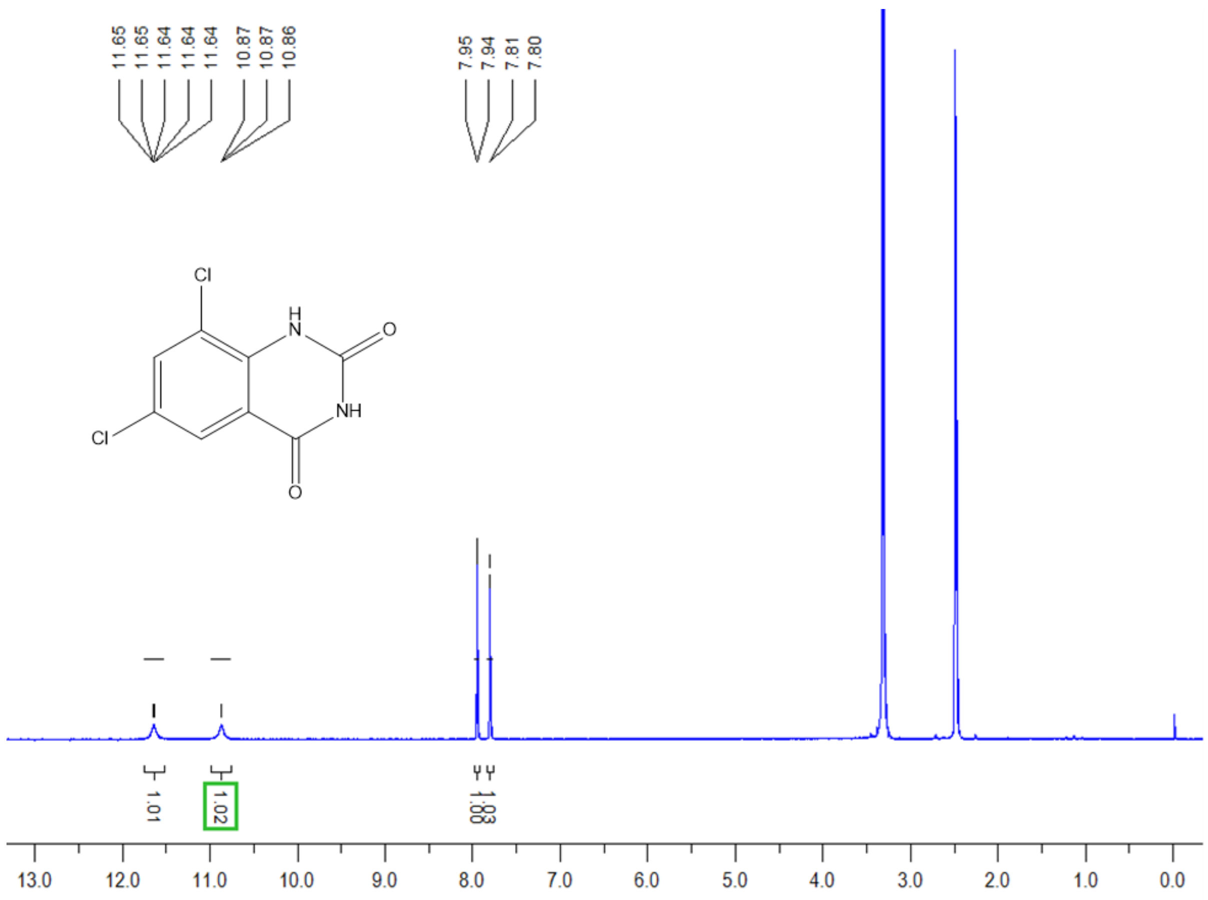

Supplementary Figure 55. ^{1}H NMR Spectra of 6,8-dichloroquinazoline-2,4(1*H*,3*H*)-dione in DMSO-d_6.

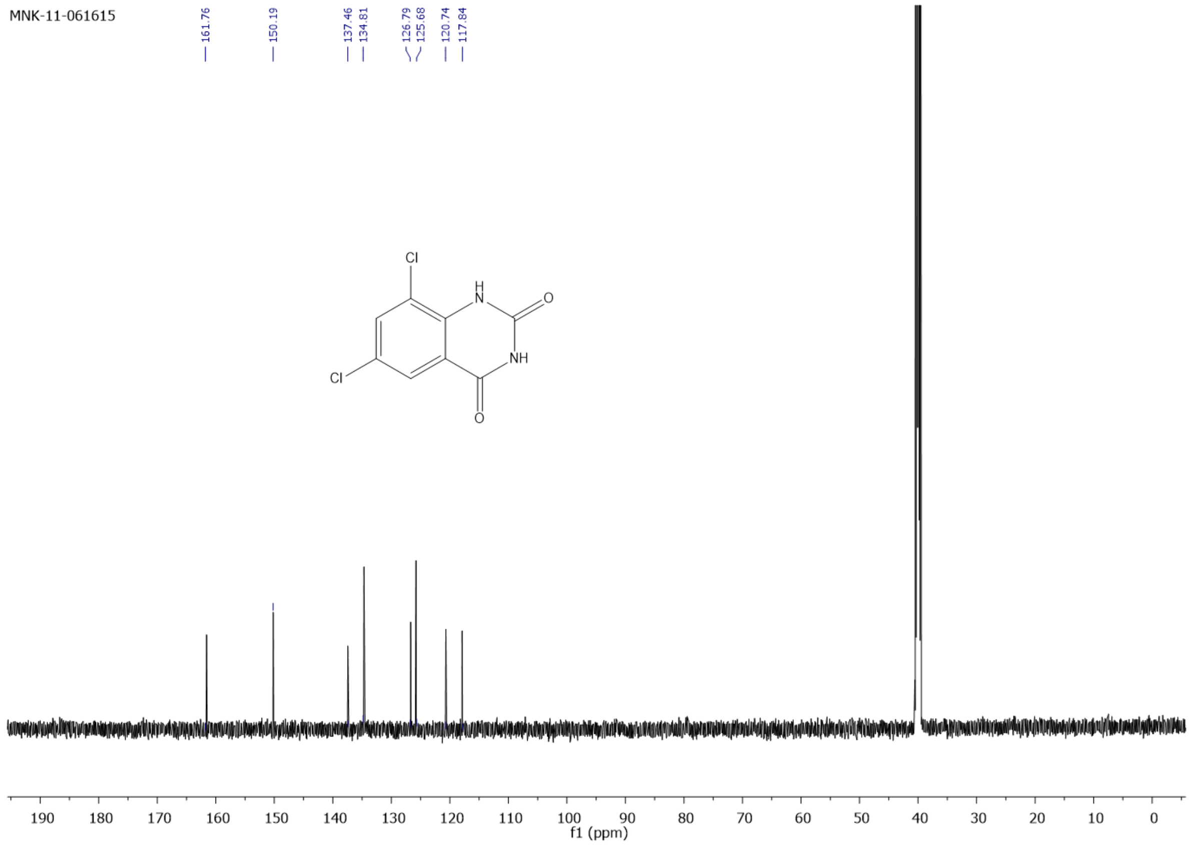

Supplementary Figure 56. ^{13}C NMR Spectra of 6,8-dichloroquinazoline-2,4(1*H*,3*H*)-dione in DMSO-d_6.

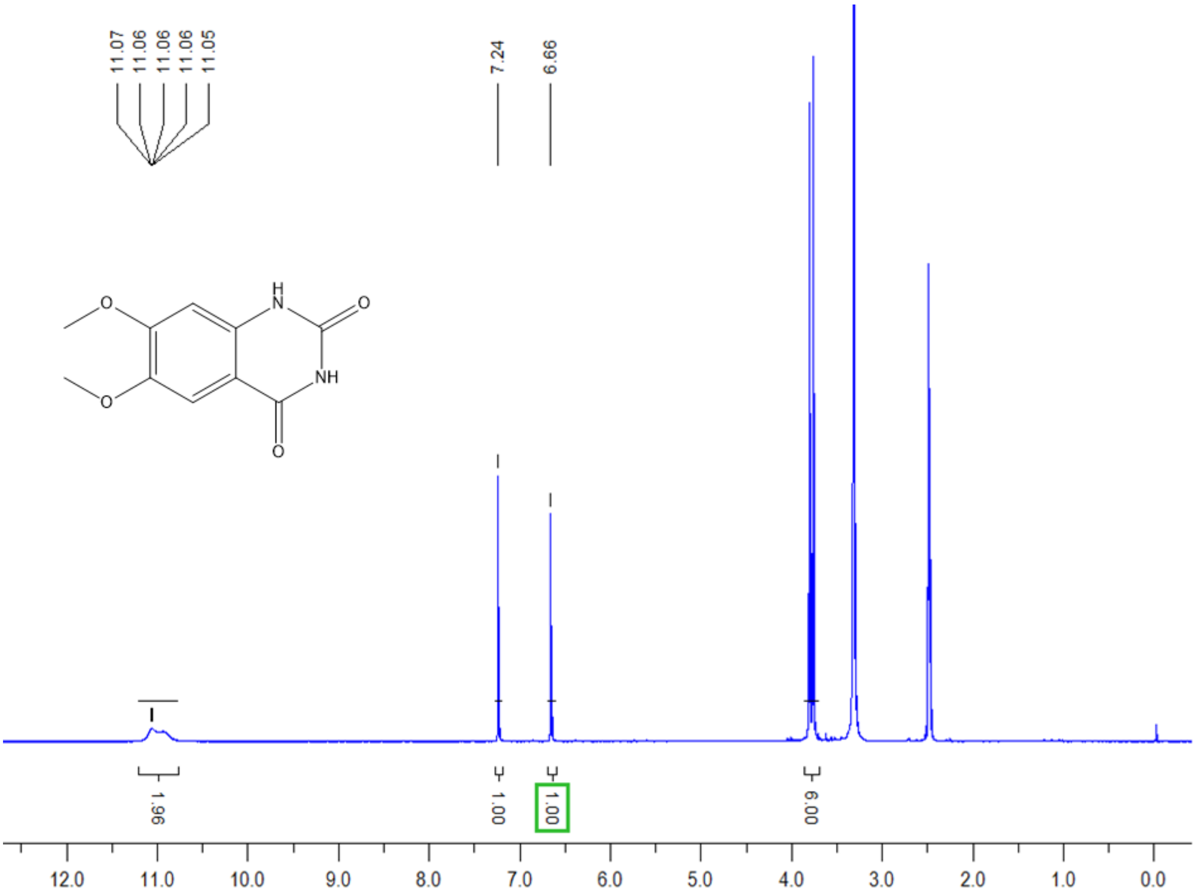

Supplementary Figure 57. ^{1}H NMR Spectra of 6,7-dimethoxyquinazoline-2,4(1*H*,3*H*)-dione in DMSO-d_6.

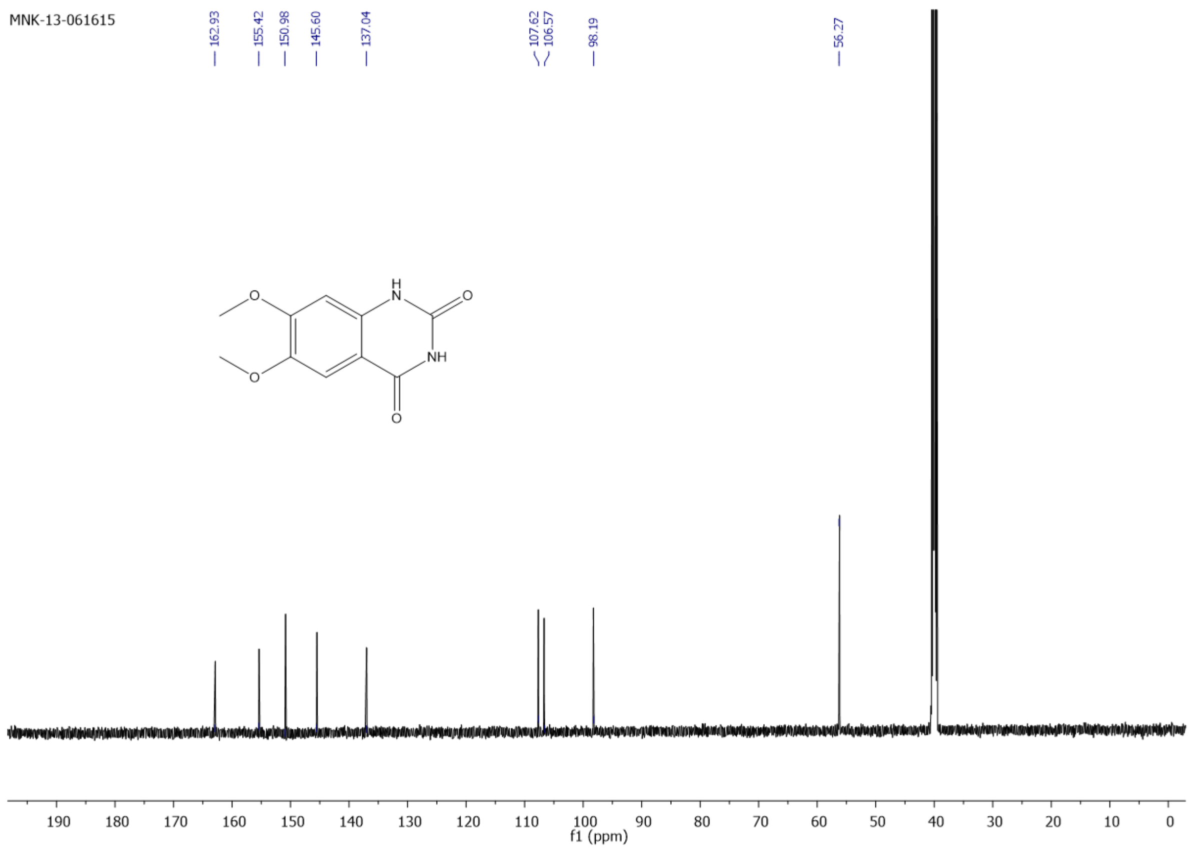

Supplementary Figure 58. ^{13}C NMR Spectra of 6,7-dimethoxyquinazoline-2,4(1*H*,3*H*)-dione in DMSO-d_6.

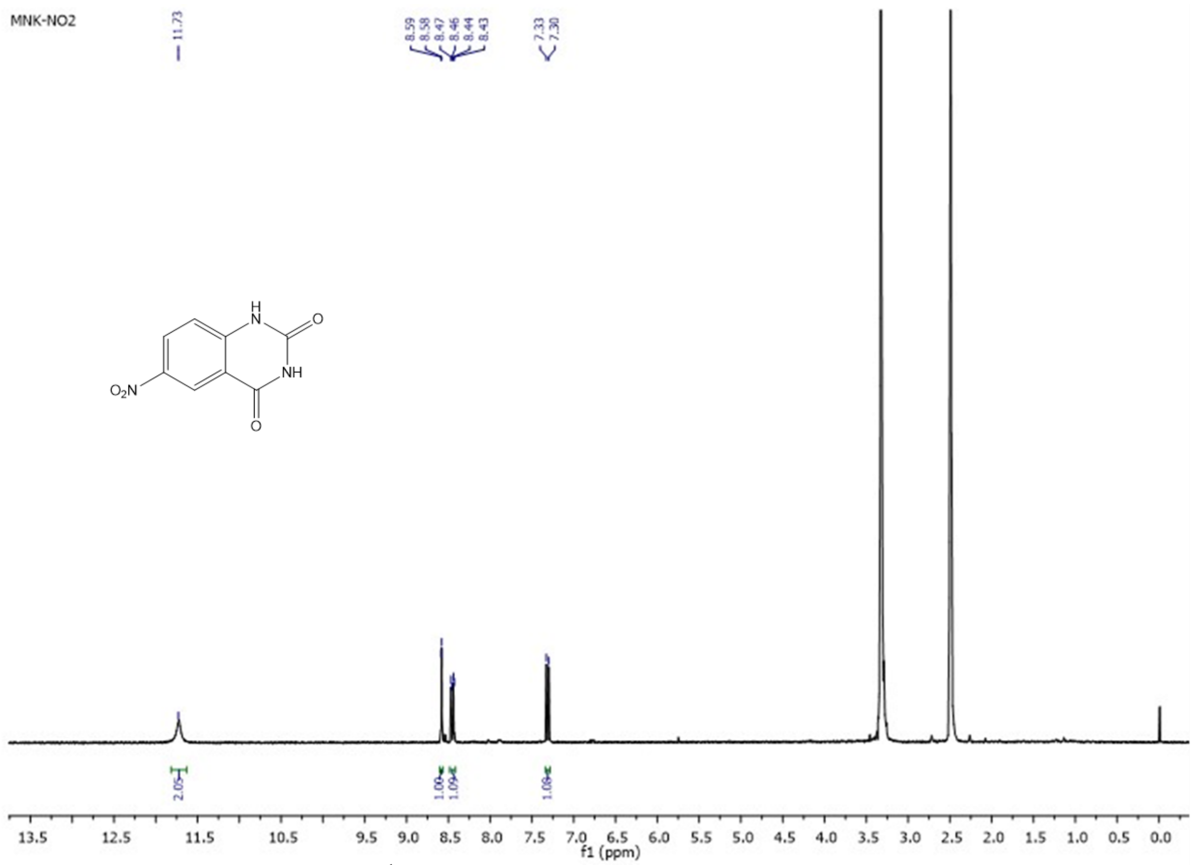

Supplementary Figure 59. ^{1}H NMR Spectra of 6-nitroquinazoline-2,4(1*H*,3*H*)-dione in DMSO-d_6.

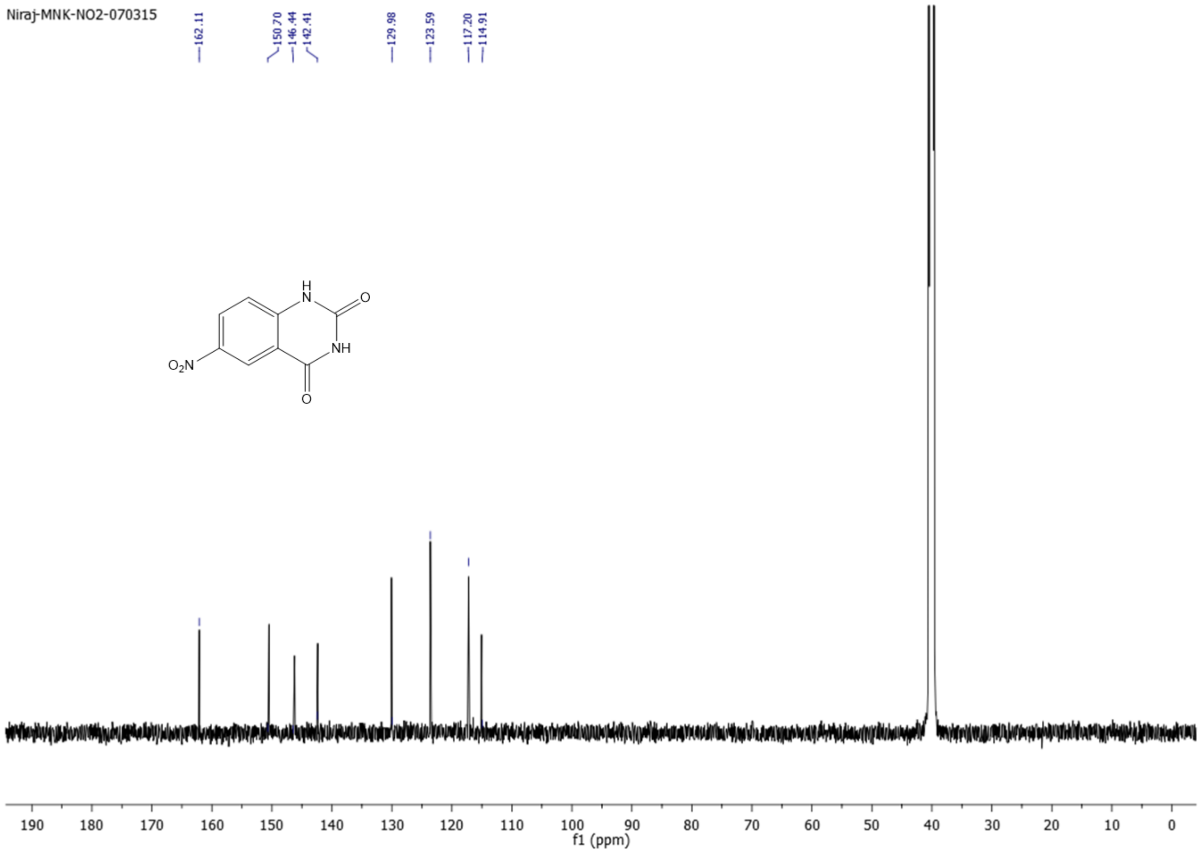

Supplementary Figure 60. ^{13}C NMR Spectra of 6-nitroquinazoline-2,4(1*H*,3*H*)-dione in DMSO-d_6.

Supplementary Tables

Supplementary Table 1. Solubility profile of various solvents for reactants, products and catalyst ILs.

Solvent	Surface Tension[a] (dynes cm^{-1})	Solubility				
		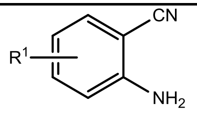	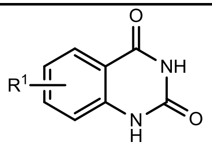	ILs $[HDBU^+][TFE^-]$ / $[HDBU^+][TFE^-]$	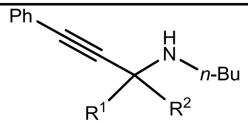	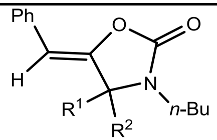
DCM	27.8	Soluble	Not Soluble	Soluble	Soluble	Soluble
Chloroform	26.7	Soluble	Not Soluble	Soluble	Soluble	Soluble
THF	26.7	Soluble	Soluble	Soluble	Soluble	Soluble
1,4-dioxane	32.9	Soluble	Soluble	Soluble	Soluble	Soluble
Ethanol	22.0	Soluble	Soluble*	Soluble	Soluble	Soluble
Acetone	23.0	Soluble	Soluble*	Soluble	Soluble	Soluble
Ethylacetate	23.2	Soluble	Soluble*	Soluble	Soluble	Soluble
Acetonitrile	28.7	Soluble	Not Soluble	Soluble	Soluble	Soluble
DMSO	42.9	Soluble	Soluble	Soluble	Soluble	Soluble

[a] At 25 ^oC; *At 50-60^oC

Supplementary Table 2. Optimization of product extraction parameters in a solvent switching work-up step.

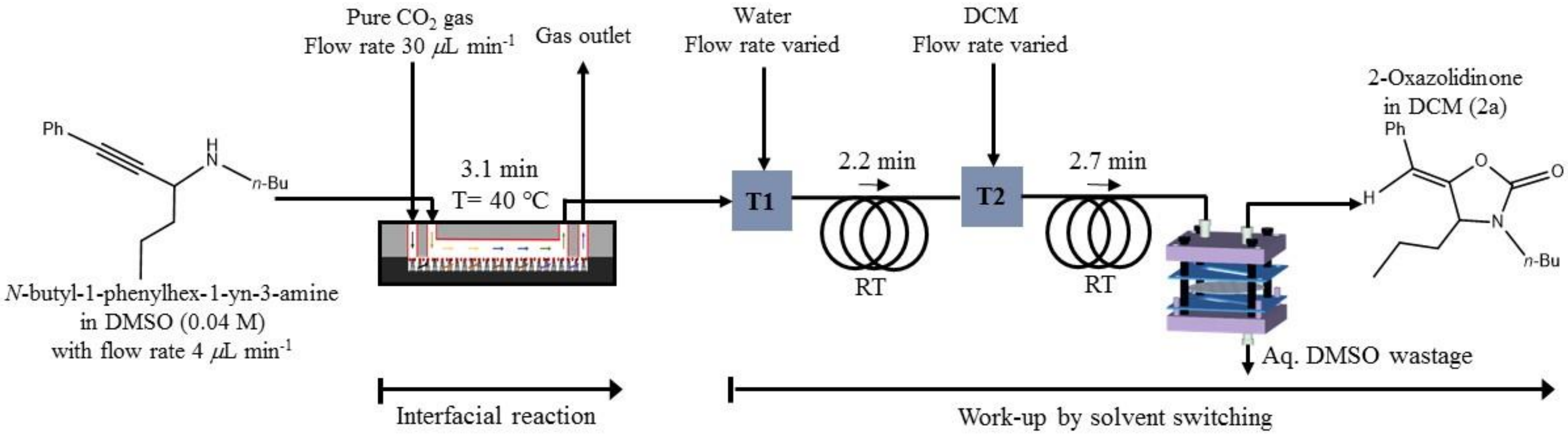

Entry	Water flow rate [μL min^{-1}]	DCM flow rate [μL min^{-1}]	Yield[a] [%]
1	4	4	12
2	8	4	22
3	16	4	50
4	24	4	74
5	32	4	90
6	36	4	92
7	40	4	93
8	40	50	74

[a] Yields are based on GC-MS using anisole as an internal standard. Reaction condition: [*N*-butyl-1-phenylhex-1-yn-3-amine] = 0.04 M, flow rate of 1a = 4 μL min^{-1}, flow rate of pure CO_2 = 30 μL min^{-1}.

Supplementary Methods

1. General Information:

Silicon wafer (100) (p-type, dopant: boron) was purchased from Semi-Materials Co., Ltd. AZ 1512 positive photoresist was purchased from AZ Electronic Materials. SU-8 negative photoresist was purchased from Micro Chem. Polydimethylsioxane (PDMS, Sylgard 184) and curing agent (Sylgard 184) were purchased from Dow Corning. Allylhydridopolycarbosilane (AHPCS, SMP-10) was purchased from Starfire System.

PTFE (id = 500 μm) tubing and T-junction were purchased from Upchurch Scientific. Co. HF solution ($\geq$ 48 wt%) was purchased from Sigma-Aldrich. H_2O_2 solution (30 wt%) was purchased from Samchun Pure Chemical Co. Ltd. Calorimetric natural gas reference standard was purchased from Sigma-Aldrich. All organic solvents and reactants from Sigma-Aldrich or Alfa Aesar or TCI chemicals were used as received without any additional purification. For experiments the deionized water with conductivity 18.2 mS was used. The chemical structures of synthesized products were fully characterized by GC/Mass-spectra, ^{1}H and ^{13}C NMR. GC/MS spectrum was recorded by Agilent 5975C GC/MSD System. ^{1}H NMR, ^{13}C NMR and HSQC NMR spectrum were recorded on a Bruker 600 or 300 MHz in either $CDCl_3$ or DMSO-d_6. High resolution SEM images were taken by Philip XL30 SEM, operating at 10^{-2} to 10^{-3} Pa with EHT 15 kV with 300 V collector bias. Platinum sputtering was implemented at pressure ranging between 1 and 0.1 Pa prior to SEM experiments. Contact angles (CAs) were measured using Smart Drop (FemtoFab). The etched fraction of SiNWs was analyzed by calculating the ratio of etched area to the whole area of SiNWs from the SEM image through Image J software.

2. Fabrication of DBU-IL catalyst embedded SiNws microreactor: The DBU-IL catalyst imbedded on thimble of SiNWs microreactor for interfacial organic synthesis was fabricated by following way:

2.1. Selective -SH functionalization on thimble of superamphiphobic SiNWs: Mercaptosilane was selectively modified the thimble of superamphiphobic SiNWs by reported method.[1] A simple flow diagram is shown in **Supplementary Fig. 1**.

Step 1. Patterning of protective AZ photoresist layer on Si wafer: Si nanowire chip were made by soft photolithographic patterning method as reported.[1,2] In this method, boron doped *p*-type silicon (100) wafer was cleaned by acetone, isopropyl alcohol followed by deionized water to remove oxidized layer and dried by N_2 flow. Then, the wafer was spin coated by AZ 1512 positive photoresist consecutively at 500 rpm and 2500 rpm for 5 sec and 30 sec, respectively, to get homogeneous thickness of AZ1512 photoresist. Subsequently, the photoresist coated silicon wafer was soft baked on a preheated hot plate at 125 ^{o}C for 10 min. Then the baked silicon wafer was covered by photo-mask (**Supplementary Fig. 1**(**b**), left) and irradiated under UV light (λ = 250-400 nm, 4.5 mW cm^{-2}) for 10 min, followed by post baking at 95 ^{o}C for 1 min. The pattern was developed by using AZ developer, and washed 5 min with water and dried by nitrogen flow (**Supplementary Fig. 1**, Patterned silicon wafer).

Step 2. Preparation of SiNWs pattern: The cone-shaped SiNWs were fabricated by silver-assisted selective etching of silicon wafer[1,2]. First, the oxide layer of patterned Si(100) wafer was removed by immersing in 1% HF solution for 1 min, washed with H_2O, and dried with nitrogen flow. The Si wafer was immediately placed into 30 mL of 10% HF solution mixed with 25.5 mg of $AgNO_3$ (5 mM) for 4.5 min to load the Ag catalyst. The Ag-coated Si wafer was washed with water, dried with nitrogen flow, and etched by being immersed in the mixture of 40 mL of 10% HF solution and 0.544 g of 30% H_2O_2 (0.4 M) for 6 h. The SiNWs pattern was washed with water and dried by nitrogen flow. The cross-sectional and top view images of SiNWs were observed by high resolution SEM as shown in **Supplementary Fig. 2**(**a**) and **2**(**b**).

Step 3. SiO_2 nanoparticles coating over SiNWs pattern: The cone shaped SiNWs wafer was immersed in a mixture of 30 mL of water, 0.24-1.2 mmol TEOS, and 0.1 mL of HCl solution for 5 h at 70 °C to decorate the SiNWs surface with silica nanoparticles (SiO_2 NP) by using 1.2 mmol of TEOS solution, but maintained the hydrophilic surface character.

Step 4. Wax coating selectively on thimble part: After decoration with SiO_2 NP, the thimble part of SiNWs was protected with wax prior to fluorination. The SiNWs sample was put on a preheated hot plate at 80 ^oC and pre-melted wax was applied on to it with help of dropper in such a way that the bottom part of SiNWs remained free from wax coating. The wax coated SiNWs clusters was removed from hot plate to solidify the wax. The SEM image in **Supplementary Fig. 2**(**c**) showed selectively wax coating on the thimble of SiNWs.

Step 5. Fabrication of superamphiphobic SiNWs pattern: Wax coated SiNWs pattern was placed on poly(styrene) petri dish with 2-3 drops of trichloro(1*H*,1*H*,2*H*,2*H*-perfluorooctyl)silane in a vial cap. The Petridis was kept on a preheated hot plate at 55 ^oC and the vaporized silane compound took place fluorination at exposed bottom part of the SiNWs with channel pattern. The Petridis was removed from the hotplate and left to come at room temperature. Wax was completely removed from the channel by washing with hexane at 40 ^oC. Washing was repeated several times to complete removal of wax.

In detail, the superamphiphobic property was tuned by varying four parameters; (1) silver-nanoparticles (Ag-NPs) loading time (t_{Ag}), (2) etching time (t_{etch}) of Si wafer, (3) SiO_2 decoration concentration of tetraethyl orthosilicate (C_{TEOS}) and (4) fluorination time (t_F) at 55 ^oC. Ag-NPs loading amount was varied by varying t_{Ag} from 2 min to 5 min. Longer loading times of Ag-NPs catalyst leaded to formation of more scattered SiNWs while $\leq$2 min of t_{Ag} resulted in highly dense SiNWs maintaining vertically aligned morphology. The t_{Ag} for cone-shaped morphology and high CA was found 4.5 min. Secondly, the length of SiNWs was controlled by t_{etch}, higher t_{etch} resulted longer nanowires height and cone-shaped SiNWs

morphology. When t_{etch} was 4-6 h, the cone-shaped SiNWs morphology gave the highest CA of DMSO and water (**Supplementary Fig. 3**). Thirdly, smooth surface of the SiNWs was gradually converted to a roughened morphology with re-entrant nanostructures by decoration with SiO_2 NP formed by sol-gel process of C_{TEOS} at 24 mM. The SiO_2 nanotexturing on the smooth surface formed a hierarchical structure that considerably enhanced the superamphiphobicity. Finally, fluorination time (t_F) of trichloroperfluorooctylsilane at 55 ^oC over the wax coated SiNWs channel. The CA of solvents (DMSO and water) gradually increased with t_F and became constant after 6 h. The highest static CA of DMSO and water were achieved 128° and 153°, respectively.

Step 6. Selective mercaptosilane immobilization only on thimble part: After selective fluorination, the cleanly protected thimble part of SiNWs with channel pattern was chemically modified to functionalize with -SH group by sol-gel reaction of (3-mercaptopropyl)trimethoxysilane that took place only at the hydrophilic part (non-fluorinated part) of SiNWs *viz*. thimble part that was protected by wax. In this step, the SiNWs pattern was immersed in a beaker containing mixture of 30 mL water, 0.5-1.2 mmol (3-mercaptopropyl)trimethoxysilane and 0.1 mL 36 % HCl at 70 ^oC. After 5 h, the SiNWs pattern was washed with water, and dried by nitrogen flow.

2.2. Synthesis of 6-allyl-1,8-diazabicyclo[5.4.0]undec-7-ene (A-DBU):

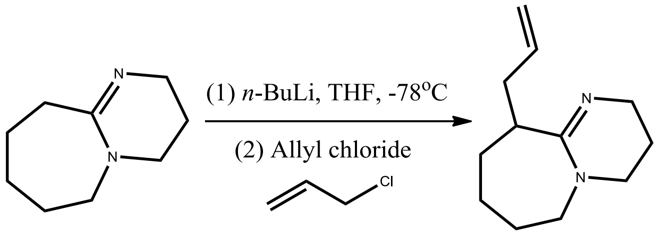

Synthesis of 6-allyl-1,8-diazabicyclo[5.4.0]undec-7-ene (A-DBU) was performed according to literature procedure.[3] A three neck 500 mL round bottom flask, equipped with magnetic

stir bar, connected with two dropping funnels, was dried and flushed with argon gas. In a typical reaction, 1,8-diazabicyclo[5.4.0]undec-7-ene (DBU) (1.02 g, 6.70 mmol) was dissolved in THF (200 mL) and maintained at -78 ^{o}C using a mixture of dry ice and acetone, then added *n*-butyl lithium (*n*-BuLi, 1.6 M in hexane) (4.4 mL, 7.04 mmol) drop-wise with the help of dropping funnel. After stirring the solution for 1 h, allyl chloride (0.56 g, 7.37 mmol) was added to the reaction mixture using another dropping funnel with continuous stirring over the period of 30 min. After addition, the solution was cooling down to room temperature and stirred for additional 3 h. Reaction was quenched by adding 10 mL methanol. The mixture was concentrated under reduced pressure and purified by column chromatography with methanol/hexane (10-50%). Yield 93%. Mass (EI) (**Supplementary Fig. 4**) m/z = 191.2 (M+); ^{1}H NMR ($CDCl_3$, 600 MHz) (**Supplementary Fig. 5**) δ: 5.82-5.91 (m, 1H), 5.11-5.18 (dd, 2H), 3.68-3.77 (m, 1H), 3.40-3.55 (m, 4H), 3.32-3.41 (m, 1H), 2.65 (dd, 1H), 2.48 (dd, 1H), 1.94-2.08 (m, 3H), 1.40-1.92 (m, 6H); ^{13}C NMR ($CDCl_3$, 150 MHz) (**Supplementary Fig. 6**) δ: 162.10, 129.98, 112.83, 48.72, 45.29, 37.44, 33.55, 28.96, 22.96, 22.46, 19.48, 19.28, 15.09; FTIR (cm^{-1}) (**Supplementary Fig. 7**) 3041 (=C-H str.), 1928, 2850 (C-H str.), 1604 (C=N str.). Carbon-Hydrogen Heteronuclear Single Quantum Coherence Nuclear Magnetic Resonance spectroscopy (HSQC NMR) spectra of monomer A-DBU in $CDCl_3$ is shown in **Supplementary Fig. 8**.

2.3. Fabrication of the modified PDMS channel and DBU-IL catalyst immobilization: Whole fabrication process was followed by reported method[1,4]. A simple flow diagram is shown in **Supplementary Fig. 9**.

2.3.1. Fabrication of SU-8 master pattern: Silicon(100) wafer was serially washed by acetone, isopropyl alcohol, deionized water and dried by nitrogen flow. The wafer was spin coated with SU-8-25, a negative photoresist, by a coating program (1) 500 rpm for 5 sec, (2)

1000 rpm for 5 sec, (3) 1000 rpm for 30 sec to get average thickness of 30 μm. The SU-8-25 coated silicon wafer was soft baked on a preheated hotplate at 75 ^{o}C for 3 min and successively at 105 ^{o}C for 7 min. The soft baked silicon wafer was then covered with photo mask (**Supplementary Fig. 1**(**b**), right) and irradiated with UV light. After 10 min exposure of UV light, the wafer was immediately post baked at 75 ^{o}C for 1 min and 105 ^{o}C for 3 min. The post baked silicon wafer was developed by SU-8 developer for 5 min to get a SU-8 pattern, and washed with isopropyl alcohol, followed by water and dried with nitrogen flow.

2.3.2. Fabrication of PDMS channel: SU-8 pattern was kept in a poly(styrene) petri dish and poured with a mixture of Sylgard 184 from Dow Corning and its curing agent (10:1, *w*/*w*). The trapped pores in the polymer were degassed in a vacuum desiccator and thermally cured in an oven at 80 ^{o}C for 2 h. The solidified PDMS was cut around the channel and peeled off, punctured by punching needle for tubing.

2.3.3. Fabrication of silicate glass coated PDMS channel: The row surface of PDMS channel was treated with O_2 plasma for 3 min prior to spin coating with allylhydridopolycarbosilane (AHPCS) solution (AHPCS, dicumyl peroxide and Irgacure 369 (100:1:1, *w*/*w*)) to strengthen the adhesion between PDMS and AHPCS. The O_2 plasma treated PDMS mold was spin coated with AHPCS solution (10 % in cyclohexanone) at 2000 rpm for 30 sec. Excess solution on the mold was wiped out gently using a glass slide, and irradiated with UV light (λ = 250-400 nm, 4.5 mW cm^{-2}) for 30 min and finally baked at 120 ^{o}C for 2 h. The optical microscope images of AHPCS coated PDMS channel is shown in **Supplementary Fig. 9**, indicating a thin AHPCS coating on channel surface as a dark layer. The Si-H groups of the cured AHPCS surface was then converted to Si-OH by hydrolysis with 0.5N NaOH solution for 2 h at 30 ^{o}C, and rinsed thoroughly with deionized water, as confirmed by a time dependent hydrolyzed FTIR spectra in **Supplementary Fig. 10**. The peak at 3500 cm^{-1}, correspond to -O-H stretching, becomes prominent as the hydrolysis time

increases. Note that the hydrolysis step in alkali condition converted the AHPCS polymer to the silicate glass layer with thin layer[5].

2.3.4. Modification of AHPCS coated PDMS surface by mercaptosilane: The Si-OH groups of AHPCS surface were transformed into -SH group by immersing the hydrolyzed AHPCS modified PDMS mold in a mixed aqueous solution of 30 mL water dissolved with 1.2 mmol (3-mercaptopropyl)trimethoxysilane and 0.1 mL HCl (36 %) for 5 h at 70 ^{o}C. Then, the PDMS mold was washed with water and dried by nitrogen flow. The appearance of peak at 2560 cm^{-1}, correspond to -SH stretching, in FTIR spectra confirmed the –SH immobilization on the AHPCS coated PDMS channel (**Supplementary Fig. 10**).

2.4. Bonding between SiNWs channel and PDMS channel: A superamphiphobic SiNWs channel embedded with –SH modified thimble part was bonded together with a modified PDMS channel immobilized with –SH by O_2 plasma treatment for 3 min, and pasted together with proper alignment, baked at 120 ^{o}C for 2 h. A schematic diagram has been shown in **Supplementary Fig. 11**. Tubes were inserted into punctured inlet and outlet and fixed with epoxy adhesive. Eventually, two types of SiNWs/PDMS microreactors were prepared either by immobilizing ILs catalyst only on the thimble of SiNWs or both thimble of SiNWs and the modified PDMS wall surface.

2.5. Two types of ILs catalyst immobilization over SiNWs microreactor:

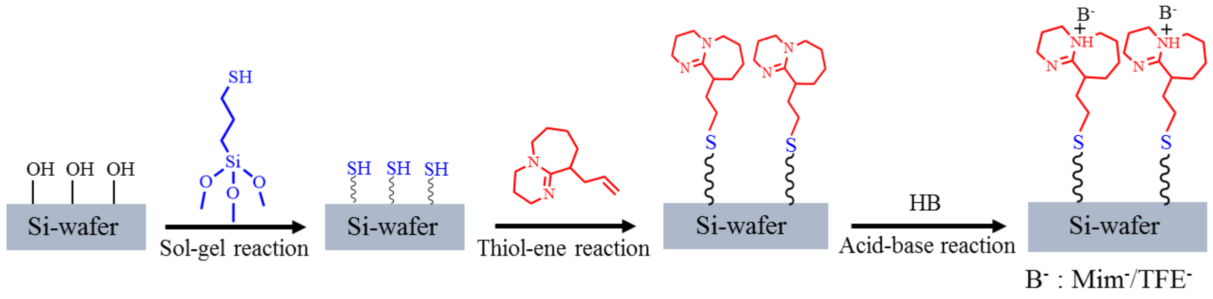

As seen at above of immobilization chemistry over SiNWs surface, thiol-ene chemistry has been exploited to immobilize the DBU by reaction of A-DBU with the -SH functionalized thimble part of SiNWs and the surface of AHPCS coated PDMS channel (**Supplementary Fig. 11**). Two types of DBU-ILs catalytic microreactors were made by immobilizing; (type 1) only on thimble of SiNWs **Supplementary Fig. 11**(**a**), (type 2) on thimble of SiNWs and AHPCS coated PDMS inner surface (**Supplementary Fig. 11**(**b**)). For type 1 of SiNWs/PDMS microreactor, the A-DBU solution in THF was filled into the channel through tubing using a syringe, then UV light (365 nm, 4.6 mW cm^{-2}) was exposed for 15 min and washed out the unreacted A-DBU by infusing THF continuously at a flow rate 50 μL min^{-1} using a syringe pump. Total amount the immobilized A-DBU was quantified by gravimetric comparison between initial and final amount of A-DBU upon repeated thrice infusions, ~0.86 mg cm^{-2} of A-DBU immobilized over the thimbles of SiNWs channel. For $[HDBU^+][MIm^-]$-ILs catalytic microreactor, the immobilized DBU was converted into its corresponding ionic liquids by infusing a solution of 2-methylimidazole (MIm) in THF at a flow rate 10 μL min^{-1} for 1 h. Similarly 2,2,2-trifluoroethanol (TFE) to get $[HDBU^+][TFE^-]$-ILs, and washed with fresh THF. ILs immobilization over the SiNWs was confirmed by SEM and EDX analysis (**Supplementary Fig. 2 & 12**). The EDX spectra of $[HDBU^+][TFE^-]$-ILs immobilized SiNWs showed a fluorine (F) elemental peak in TFE (**Supplementary Fig. 12**(**b**)) while sulphur peak was shown from mercaptosilane modified SiNWs for $[HDBU^+][MIm^-]$-IL immobilization (**Supplementary Fig. 12**(**a**)).

3. Integrated platform for serial CO_2 capture-fixation and subsequent work-up process:

3.1. Selection of a suitable solvent for DBU-ILS catalytic microreaction: A number of solvents were tested, and DMSO was found to facilitate complete solubility of the reactant

and product, and provide desired contact angle over SiNWs due to its high surface tension (**Supplementary Table 1**).

3.2. Synthesis of propargylic amine(s): Propargylic amines (1a-1f) were synthesized by typical method given below

Typical synthesis of butyl-(1-phenylethynyl-butyl)-amine (1a): Propargylic amines were synthesized by as reported method[6]. Typically, in a schlenk tube, equipped with teflon coated magnetic stir bar, CuI (571 mg, 3 mmol) was taken and flushed with argon gas. Ethanol (2 mL), butyraldehyde (1.44 g, 20 mmol) and *n*-butylamine (1.46 g, 20 mmol) were added and stirred at room temperature for 30 min. Then, phenylacetylene (1.02 g, 10 mmol) was added to the reaction mixture under argon atmosphere with gentle stirring. The schlenk tube was placed in preheated oil bath at 80 ^{o}C for overnight. Reaction mixture was then filtered through neutral alumina column to remove copper complex. Solvent was evaporated up to dryness and purified by silica gel column chromatography (ethyl acetate: hexane = 2:5).

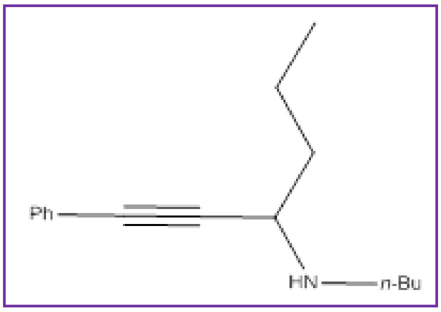

***N*-Butyl-(1-phenylethynyl-butyl)-amine (1a):** brown oil, ^{1}H NMR ($CDCl_3$, 300MHz) δ: 7.35 (dd, J = 2.25, 1.0 Hz, 2H), 7.33-7.28 (m, 2H), 3.54 (dd, J = 5.7, 2.4 Hz, 1H), 2.86 (ddd, J = 6.4, 3.7, 2.1 Hz, 1H), 2.62 (ddd, J = 6.4, 3.7, 2.1 Hz, 1H), 1.66-1.50 (m, 2H), 1.52-1.36 (m, 4H), 1.36-1.26 (m, 2H), 0.999 (t, J = 7.4 Hz, 3H), 0.964 (t, J = 7.4 Hz, 3H); ^{13}C NMR ($CDCl_3$, 150MHz) δ: 131.65, 128.21, 127.81, 123.52, 91.46, 83.45, 50.63, 47.31, 38.31, 32.33, 20.54, 19.43, 14.08. The observed characterization data (^{1}H and ^{13}C) were consistent with reported literature[7].

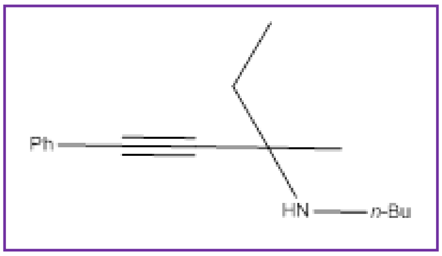

Butyl-(1-ethyl-1-methyl-3-phenylprop-2-ynyl)-amine (1b): yellow oil, ^{1}H NMR ($CDCl_3$, 300MHz) δ: 7.34 (dd, J = 7.5,

2.2 Hz, 2H), 7.28-7.34 (m, 3H), 2.63-2.77 (m, 2H), 1.55-1.67 (m, 2H), 1.46 (s, 3H), 1.31 (m, 4H), 1.18 (s, 1H), 0.98 (t, J = 7.4 Hz, 3H), 0.86 (t, J = 7.4 Hz, 3H); ^{13}C NMR ($CDCl_3$, 150MHz) δ: 131.62, 128.26, 127.72, 123.62, 93.85, 83.22, 54.36, 43.58, 34.70, 32.76, 29.90, 26.21, 20.53, 14.11.

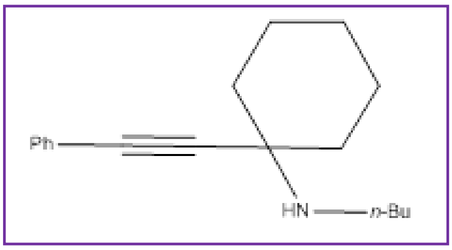

Butyl-(1-phenylethynyl-cyclohexyl)-amine (1c): brown oil, ^{1}H NMR ($CDCl_3$, 300MHz) δ: 7.43-7.47 (dd, J = 6.2, 2.0 Hz, 2H), 7.29-7.34 (m, 3H), 2.74 (t, J = 7.2 Hz, 1H), 1.88 (d, J = 12.3 Hz, 1H), 1.62 (t, J = 10.3 Hz, 5H), 1.29-1.49 (m, 7H), 1.10-1.23 (m, 1H), 0.88 (t, J = 7.2 Hz, 3H); ^{13}C NMR ($CDCl_3$, 150MHz) δ: 131.61, 128.25, 127.54, 123.87, 93,67, 84.49, 55.03, 42.87, 38.18, 32.94, 25.95, 23.09, 20.69, 14.03. The observed characterization data (^{1}H and ^{13}C) were consistent with reported literature[7].

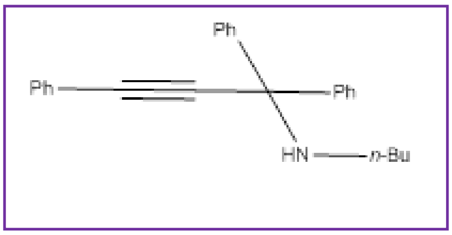

***N*-(1,1,3-triphenylprop-2-ynyl)butan-1-amine (1d):** yellow oil, ^{1}H NMR ($CDCl_3$, 300MHz) δ: 7.34-7.42 (m, 6H), 7.28-7.36 (m, 9H), 3.06 (t, J = 7.2 2H), 1.55 (m, 2H) 1.32-1.42 (m, 2H), 0.93 (t, J = 7.2 Hz, 3H); ^{13}C NMR ($CDCl_3$, 150MHz) δ: 137.98, 131.34, 129.72, 128.26, 127.84, 126.63, 96.45, 85.41, 65.19, 42.45, 29.60, 19.73, 13.93.

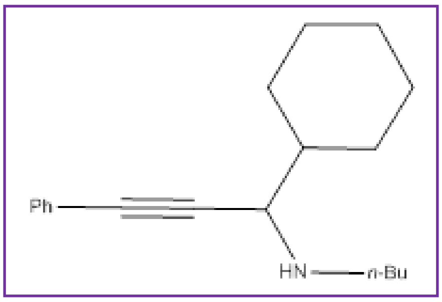

Butyl-(1-cyclohexyl-3-phenyl-prop-2-ynyl)-amine (1e): brown oil, ^{1}H NMR ($CDCl_3$, 300MHz) δ: 7.35 (dd, J = 7.5, 2.1 Hz, 2H), 7.30-7.34 (m, 3H), 3.42 (d, J = 5.6 Hz, 1H), 2.92-2.98 (m, 1H), 2.63-2.70 (m, 1H), 1.76-1.96 (m, 4H), 1.67-1.75 (d, J= 12.4 Hz, 1H), 1.35-1.67 (m, 7H), 1.25-1.35 (m, 4H), 0.91 (t, J = 7.38 Hz, 3H); ^{13}C NMR ($CDCl_3$, 150MHz) δ: 131.65, 128.34, 127.64, 123.66, 90.43, 84.31, 56.39, 42.65, 32.22, 30.46, 28.54, 26.53, 25.93, 20.51, 14.02.

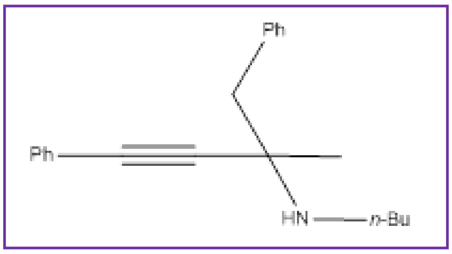

***N*-butyl-2-methyl-1,4-diphenylbut-3-yn-2-amine (1f):** yellow oil, ^{1}H NMR ($CDCl_3$, 300MHz) δ: 7.39 (dd, J = 7.5, 2.2 Hz, 2H), 7.28-7.35 (m, 5H), 2.97 (s, 2H), 2.79 (m, 3H), 1.51 (s, 3H), 1.36-1.45 (m, 5H), 0.92 (t, J = 7.2 Hz, 3H), 0.86 (t, J = 7.4 Hz, 3H); ^{13}C NMR ($CDCl_3$, 150MHz) δ: 131.35, 130.57, 128.04, 127.84, 126.62, 123.33, 94.16, 83.38, 44.09, 43.88, 32.74, 29.60, 27.15, 20.73, 13.95.

3.3. Chemical reactions in the DBU-ILs catalytic microreactors

3.3.1. Synthesis of 5-benzylidene-3-butyl-4-propyl-oxazolidin-2-one (2a): A stock solution of *N*-butyl-(1-phenylethynyl-butyl)-amine (1a) (0.04 M) was prepared by dissolving 0.916 g of 1a in 100 mL DMSO. The reaction was proceeded by infusing a solution of 1a (flow rate 4-8 μL min^{-1}) to the upper PDMS channel and pure CO_2 (flow rate 30-50 μL min^{-1}) to the lower SiNWs channel of microreactor using individual leak proof syringe (**Supplementary Fig. 26**). Residence time of reactant and flow rate of gas were varied to obtain the product with different yields determined by GC-MS. Best conversion (98%) was achieved at 4 μL min^{-1} flow rate (188 sec retention time) of 1a (**Table 1(a) Entry 3)**. Molar ratio of CO_2 to propargylic amine, 1a, was varied from 5.6 to 8.4 while in the case of natural gas molar ratio varied from 9.8-13.16. Remained gas was collected from the outlet of microreactor, and analyzed by GC-MS. The CO_2 contents in the natural gas was decreased ~0.34% (2.61% from 2.95%) by chemical adsorption by DMSO solvent (γ_1) and reactant/product (γ_2)[8] (0.15 mol%) and consumption for chemical conversion (0.19 mol%), which is consistent with the calculated amount from mass balance, *ca* 1.0 (CO_2/amine) (**Supplementary Fig. 28**). Details calculation is given below.

The molar ratio calculations of reagent and CO_2 gas:

(**Table 1(a), Entry 1**)

No. of moles per min of CO_2 at 40 μL min^{-1} flow = $(40 \times 10^{-6})/22.4 = 1.78 \times 10^{-6}$

No. of moles per min of 1a at 8 μL min^{-1} flow = 0.04 × (8 × 10^{-6}) = 0.32 × 10^{-6}

Mole ratio CO_2/amine 1a = (1.78 × 10^{-6}) /(0.32 × 10^{-6}) = 5.6

(**Table 1(a), Entry 3**)

No. of moles per min of CO_2 at 30 μL min^{-1} flow = (40 × 10^{-6})/22.4 = 1.34 × 10^{-6}

No. of moles per min of 1a at 4 μL min^{-1} flow = 0.04 × (4 × 10^{-6}) = 0.16 × 10^{-6}

Mole ratio CO_2 per amine 1a = (1.34 × 10^{-6}) /(0.16 × 10^{-6}) = 8.4

(**Table 1(a), Entry 5**)

No. of moles per min of CO_2 from natural gas (2.95 mol% CO_2) at 30 μL min^{-1} flow

= (30 × 10^{-6}/22.4) × (2.95/100) = 3.95 × 10^{-8}

No. of moles per min of 1a at 4 μL min^{-1} flow = 0.001 × (4 × 10^{-6}) = 0.4 × 10^{-8}

Mole ratio CO_2/amine(1a) = (3.95 × 10^{-8}) /(0.4 × 10^{-8}) = 9.87

(**Table 1(a), Entry 6**)

No. of moles/min of CO_2 from natural gas (2.95 mol% CO_2) at 30 μL min^{-1} flow

= (30 × 10^{-6}/22.4) × (2.95/100) = 3.95 × 10^{-8}

No. of moles per min of 1a at 3 μL min^{-1} flow = 0.001 × (3 × 10^{-6}) = 0.3 × 10^{-8}

Mole ratio CO_2/amine(1a) = (3.95 × 10^{-8})/(0.3 × 10^{-8}) = 13.16

The molar ratio calculations in reaction for mass balance check (Table 1(a), Entry 6):

CO_2 in natural gas at gas inlet = 2.95 mol% (GC analysis, Supplementary Fig. 28(a))---- (α)

CO_2 in natural gas at gas outlet = 2.61 mol% (GC analysis, Supplementary Fig. 28(c))--- (β)

CO_2 absorption in DMSO = 2.95 - 2.94 = 0.01 mol% (GC analysis, Supplementary Fig. 28(b))

------- (γ_1)

Gravimetrically analysis of absorbed CO_2 by liquid phase:

Some part of CO_2 could be absorbed by DMSO solvent (γ_1) and reactant/product (γ_2).[8] The qualitative analysis of absorbed CO_2 peak was confirmed by FTIR spectrum which one shown in **Supplementary Fig. 28(d)**.

Flow rate of reactant solution = 3μL min^{-1} = 180 μl/h

Weight of solution after 1 h without natural gas flow = 203.4131 $\times 10^{-3}$ g

Weight of solution after 1 h with natural gas flow = 203.4142 $\times 10^{-3}$ g

Absorption rate of CO_2 = 11 $\times 10^{-7}$ g h^{-1} = 1.83 $\times 10^{-8}$ g min^{-1}

Molar absorption rate of CO_2 = (1.83 $\times 10^{-8}$ g min^{-1}) / (44 g mol^{-1}) = 0.042 $\times 10^{-8}$ mol min^{-1}

30 μL natural gas = 3.95 $\times 10^{-8}$ moles = 13.16 mol%

Then 0.042 $\times 10^{-8}$ moles = 13.16 $\times$ 0.042 $\times 10^{-8}$/ 3.95 $\times 10^{-8}$

= 0.140 mol%

CO_2 absorption by reactant/product amines = 0.140 mol% (Gravimetrically analysis)[8] ----(γ_2)

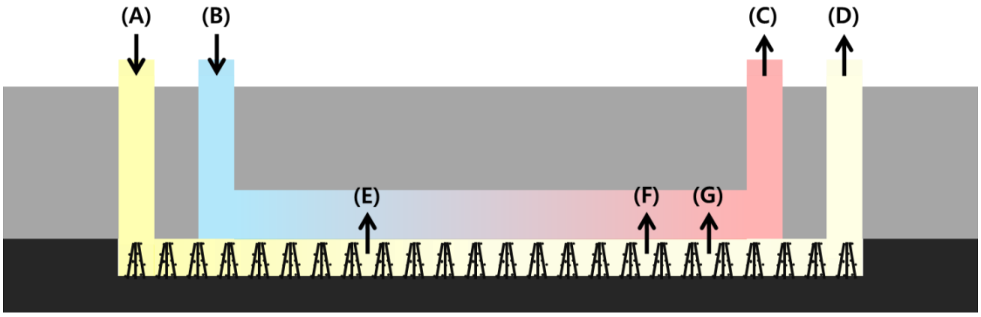

(A): CO_2 in natural gas at gas inlet

(B): Reactant solution at liquid inlet

(C): Remained reactant/product solution at liquid outlet

(D): Remained CO_2 in natural gas at gas outlet

(E): CO_2 consumed for chemical conversion

(F): CO_2 absorbed by DMSO

(G): CO_2 absorbed by reactant/product amines

Molar ratio of (A) = α

Molar ratio of (B) = α / 13.16

Molar ratio of (C) = (α / 13.16) + ($\chi + \gamma_1 + \gamma_2$)

Molar ratio of (D) = β

Molar ratio of (E) = $\chi = \alpha - (\beta + \gamma_1 + \gamma_2)$

Molar ratio of (F) = γ_1

Molar ratio of (G) = γ_2

CO_2 consumed for chemical conversion = α - (β + γ_1 + γ_2) = 0.188 mol% ---------------- (χ)

$$Conversion\ of\ amine\ to\ product = \frac{Moles\ of\ reacted\ amine}{Moles\ of\ total\ amine} = \frac{Moles\ of\ reacted\ CO_2}{Moles\ of\ total\ amine} = \frac{\chi}{\alpha/13.16}$$

$$= \frac{\alpha - (\beta + \gamma 1 + \gamma 2)}{\alpha/13.16} = \frac{2.95 - (2.61 + 0.01 + 0.140)}{2.95/13.16} = 0.85 = 85\%$$

Mass balance 1a for reaction

= calculated amine conversion from consumption CO_2 /experimental amine conversion

= 0.85/(0.87±0.02) = ~1

Visualize the liquid/gas interface in ILs catalyst loading SiNWs cluster

To visualize the interfacial position between gas and liquid in $[HDBU^+][MIm^-]$ immobilized SiNWs channel, the SiNWs cluster was dipped in BODIPY 505/515 (2 μg mL^{-1}; 4,4-difluoro-1,3,5,7-tetramethyl-4-bora-3a,4a-diaza-s-indacen) dye in 1% DMSO solution and agitated for 1 min and incubated in darkness for 10 min at room temperature. The SiNws cluster was cut into pieces (20mm × ~2mm) and a piece was fixed vertically on a glass side with adhesive tape. Images were acquired with a Nikon (Japan) Eclipse Ti-U Epi-fluorescence microscope under blue light excitation (488 nm), as seen in **Supplementary Fig. 2(f)**.

Supplementary Movie 1. The stable laminar flow between gas and liquid was demonstrated by injecting the green dyed DMSO into an upper PDMS channel (10 μL min^{-1}) and the nitrogen gas into a bottom SiNWs channel (75 μL min^{-1}), which was flowed throughout a 80 cm long DBU-IL catalytic microreactor with direct interface contact. Neither bubbles were observed in the green dyed DMSO flow nor dyed DMSO was seen in the gas outlet

(**Supplementary Fig. 27**). This phenomena confirmed the stable gas-liquid laminar flow in the SiNWs microreactor.

3.3.2. Stability test of [HDBU$^+$][MIm$^-$] immobilized microreactor: To test the stability of [HDBU$^+$][MIm$^-$]-ILs immobilized on the thimble of SiNWs, the microreactor was run continuously for four days at a flow rate 4 μL min^{-1} of reactant 1a (0.04 M) and 30 μL min^{-1} of CO_2, and the product was collected at daily base. In the beginning of 1st to 2nd days, yield of product fall gradually from 98 to 92% then after became constant at ~90%. The graph between the yields of product and time has been shown in **Supplementary Fig. 29**.

3.3.3. Typical procedure to solvent switching in an integrated continuous-flow manner: To switch the solvent containing the product from DMSO to DCM, the additional PTFE membrane embedded phase separator was connected to outlet of the SiNWs/PDMS microreactor as similarly as reported[9,10] (**Supplementary Fig. 31**). The phase separator was fabricated as following: polyethylene films (PE, 60 mm × 60 mm × 240 μm thickness) were manually punched to form a single groove with rectangular shape (8.2 mm × 35.5 mm) (**Supplementary Fig. 32**). The 4-corners of two PE film were holed (1 mm diameter) to align the film patterns. Polytetrafluoroethylene (PTFE) membrane (Whatman, 0.45 μm pore, 37 mm diameter) was sandwiched by two PE sheets with identical dimension of grooved channels, and aligned to each other by inserting metal pins through the holes at the film corners. Finally, the metal holder was tightly pressed by screws to seal the device with no leak. A serial process of droplet formation, extraction and separation for purification of 2a was conducted in droplet microfluidics equipped with the PTFE membrane phase microseparator, as explained in a step-wise manner at the below.

1. Solvent switching and extraction: water was introduced with flow rate 40 μL min^{-1} to the outlet of microreactor containing product in DMSO through T-junction (T1) (capillary length

500 mm, id = 500 μm). Excess of water turned organic phase (DMSO) into aqueous-like phase which also decrease the solubility of product, 2a, in the medium. After complete mixing (2.2 min) of DMSO with water, DCM was introduced through T-junction (T2) (capillary length 500 mm, id = 500 μm) to produce organic segments from the aqueous-like DMSO/water. Continuous flow of segments provided product extraction into the DCM phase from DMSO/water due to high solubility of product in DCM. This process was taken 2.7 min.

2. Phase separation: the DCM phase containing product, 2a, could wet thin PFPE membrane and permeated to the opposite channel of the phase separator, whereas the waste containing aqueous phase did not wet the hydrophobic membrane and maintained at the original stream. Note that low flow rate of water resulted in incomplete extraction of product into DCM. **Supplementary Table 2** shows the flow rate dependent efficiency of product extraction in DCM. The isolated product was concentrated under reduced pressure and purified using column chromatography on 200-300 mesh silica gel with hexanes/ethyl acetate as an eluent. The ^{1}H and ^{13}C NMR spectra of all 2-oxazolidinones are shown in **Supplementary 34-45**.

Supplementary Movie 2. In order to demonstrate the solvent switching performance from DMSO to DCM, we have used green color dye in water to mix with DMSO for making visible color solution (green droplet in video). The colorless DCM was infused to form continuously organic and water droplets for extracting the product from DMSO/water phase into DCM phase. Then the both droplets were continuously separated by selective wetting behavior in a PTFE imbedded phase separator.

Supplementary Movie 3. In order to show the extraction performance of heterocyclic product (yellowish color) dissolved in DMSO, DMSO/water mixture was segmented by infusion of DCM and the product extraction from DMSO/water into DCM was completed

during continuous flow of droplets as per our experimental scheme. Eventually, the extracted yellowish product in DCM solution penetrated through the PTFE membrane of phase separator and collected in vial to isolate the product, while hydrophilic DMSO/water droplet could not penetrate the PTFE membrane and was gone to waste.

NMR Data

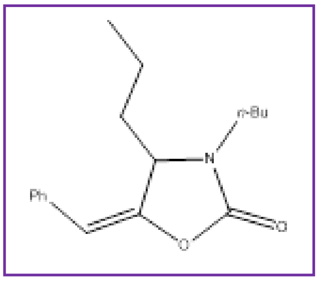

5-benzylidene-3-butyl-4-propyl-oxazolidin-2-one (2a): yellow oil, ^{1}H NMR ($CDCl_3$, 300MHz) δ: 7.60 (d, J = 14.6 Hz, 2H), 7.35 (t, J = 14.6 Hz, 2H), 7.23 (t, J = 14.6, 1H), 5.47 (s, 1H), 4.53 (s, 1H), 3.54-3.67 (m, 1H), 2.96-3.08 (m, 1H), 1.77-1.94 (m, 1H), 1.48-1.76 (m, 3H), 1.29-1.46 (m, 4H), 0.98 (t, J = 14.7, 6H); ^{13}C NMR ($CDCl_3$, 150MHz) δ: 155.18, 147.10, 133.63, 128.76, 128.15, 126.75, 102.22, 58.42, 41.04, 34.35, 29.35, 19.98, 15.85, 13.83, 13.67. The observed characterization data (^{1}H and ^{13}C) were consistent with reported literature[7].

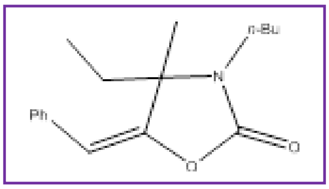

5-benzylidene-3-butyl-4-methyl-4-ethyl-oxazolidin-2-one (2b): pale yellow oil, ^{1}H NMR ($CDCl_3$, 300MHz) δ: 7.61 (dd, J = 16.6, 2.6 Hz, 2H), 7.33 (t, J = 14.6 Hz, 2H), 7.21 (tt, J = 14.6, 3.8 Hz, 1H), 5.40 (s, 1H), 3.23-3.38 (ddd, J = 28.4, 20.2, 11.5 Hz, 1H), 2.93-3.07 (ddd, J = 28.4, 20.3, 11.4 Hz, 1H), 1.78-1.93 (m, 1H), 1.54-1.75 (m, 3H), 1.49 (s, 3H), 1.29-1.46 (m, 2H), 0.92-1.00 (t, J = 14.4 Hz, 3H), 0.78-0.87 (t, J = 14.6 Hz, 3H); ^{13}C NMR ($CDCl_3$, 150MHz) δ: 154.77, 152.80, 133.73, 128.46, 128.29, 126.69, 100.74, 66.25, 40.51, 32.87, 31.34, 27.13, 20.34, 13.73, 8.05.

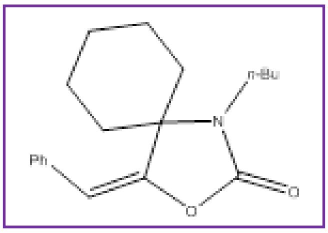

4-benzylidene-1-butyl-3-oxa-1-aza-spiro[4.5]decan-2-one (2c): light yellow solid, ^{1}H NMR ($CDCl_3$, 300MHz) δ: 7.63 (dd, J = 16.4, 2.7 Hz, 2H), 7.35 (t, J = 14.6 Hz, 2H), 7.23 (tt, J = 14.7, 2.5 Hz, 1H), 5.81 (s, 1H), 3.21 (t, J = 15.7 Hz, 2H), 1.74-1.90 (m, 7H), 1.59-1.74 (m, 4H),

1.18-1.45 (m, 3H), 0.98 (t, J = 14.5 Hz, 3H); ^{13}C NMR ($CDCl_3$, 150MHz) δ: 154.50, 152.42, 133.79, 128.74,128.42, 126.75, 103.70, 63.89, 40.23, 34.24, 31.75, 30.91, 24.19, 21.61, 20.32, 13.54. The observed characterization data (^1H and ^{13}C) were consistent with reported literature[7].

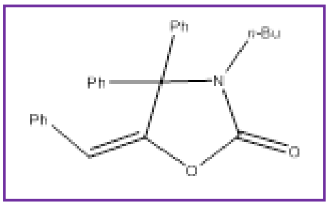

4-benzylidene-1-butyl-3-oxa-1-aza-spiro[4.5]decan-2-one (2d): yellow oil, ^1H NMR ($CDCl_3$, 300MHz) δ: 7.47 (d, J = 16.4 Hz, 2H), 7.28-7.37 (m, 9H), 7.19 (dd, J = 14.7, 2.5 Hz, 4H), 5.19 (s, 1H), 2.99 (t, J = 15.7 Hz, 2H), 1.50-1.60 (m, 2H), 1.29-1.36 (m, 2H), 0.93 (t, J = 14.5 Hz, 3H); ^{13}C NMR ($CDCl_3$, 150MHz) δ: 154.91, 146.08, 135.04, 133.39, 129.70, 128.47, 127.40, 126.61, 103.68, 59.62, 41.57, 28.80, 20.06, 13.50.

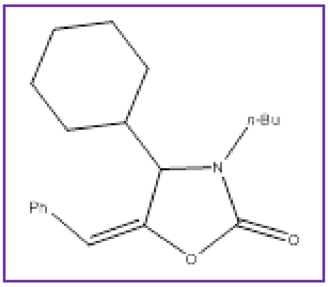

5-benzylidene-3-butyl-4-cyclohexyl-oxazolidin-2-one (2e): pale yellow oil, ^1H NMR ($CDCl_3$, 300MHz) δ: 7.61 (d, J = 14.7 Hz, 2H), 7.36 (t, J = 14.7 Hz, 2H), 7.21 (t, J = 14.8, 1H), 5.48 (s, 1H), 4.25 (s, 1H), 3.59-3.72 (dt, J = 27.5, 16.6 Hz, 1H), 3.01-3.12 (ddd, J = 28.2, 16.8, 10.2Hz, 1H), 1.60-1.93 (m, 7H), 1.29-1.45 (m, 4H), 1.06-1.24 (m, 4H), 0.92-1.01 (t, J = 14.5 Hz, 3H); ^{13}C NMR ($CDCl_3$, 150MHz) δ: 155.49, 145.59, 133.60, 128.47, 126.89, 104.28, 63.34, 41.41, 40.37, 29.17, 27.99, 26.43, 25.99, 19.96,13.62.

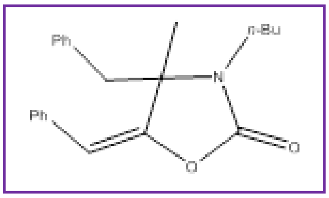

4-benzylidene-1-butyl-3-oxa-1-aza-spiro[4.5]decan-2-one (2f): yellow oil, ^1H NMR ($CDCl_3$, 300MHz) δ: 7.51 (d, J = 16.4 Hz, 2H), 7.20-7.36 (m, 6H), 7.10 (dd, J = 14.7, 2.5 Hz, 2H), 5.40 (s, 1H), 3.40-3.54 (m, 1H), 2.86-2.20 (m, 3H), 1.70-1.85 (m, 1H), 1.60 (s, 3H), 1.32-1.45 (m, 3H), 0.98 (t, J = 14.5 Hz, 3H); ^{13}C NMR ($CDCl_3$, 150MHz) δ: 154.50, 152.42, 133.79, 128.74,128.42, 126.75, 103.70, 63.89, 40.23, 34.24, 31.75, 30.91, 24.19, 21.61, 20.32, 13.54.

3.3.4. Typical synthesis of quinazoline-2,4(1*H*,3*H*)-dione (3a):

A stock solution of 2-aminobenzonitrile (0.1 M) in DMSO was prepared. The reaction was proceeded by infusing a solution of 2-aminobenzonitrile (flow rate 3-5 μL min^{-1}) to the upper PDMS channel and pure CO_2 gas with flow rate 30-50 μL min^{-1} to the lower SiNWs channel

of microreactor using individual leak proof syringe. Residence time of reactant and flow rate of gas were varied to obtain the product with different yields determined by GC-MS. The highest yield (97%) was achieved at 3 μL min^{-1} flow rate at 251 sec retention time of 2-aminobenzonitrile (**Table 1**(**b**), **Entry 3**). After completion of reaction, DCM was infused to the outlet of the microreactor. The product (quinazoline-2,4(1*H*,3*H*)-dione) was precipitated in DCM to obtain by filtering, while reactant (2-aminobenzonitrile) was dissolved to be drained out. Molar ratio of CO_2 to 2-aminobenzonitrile was varied from 3.1 to 5.2 as calculations are given below.

(**Table 1(b), Entry 1**)

No. of moles per min of CO_2 at 35 μL min^{-1} flow = $(35 \times 10^{-6})/22.4 = 1.56 \times 10^{-6}$

No. of moles per min of 2-aminobenzonitrile at 5 μL min^{-1} flow = $0.1 \times (5 \times 10^{-6}) = 0.5 \times 10^{-6}$

Mole ratio CO_2/2-aminobenzonitrile = $(1.56 \times 10^{-6}) / (0.5 \times 10^{-6}) = 3.1$

(**Table 1(b), Entry 3**)

No. of moles per min of CO_2 at 35 μL min^{-1} flow = $(35 \times 10^{-6})/22.4 = 1.56 \times 10^{-6}$

No. of moles per min of 2-aminobenzonitrile at 3 μL min^{-1} flow = $0.1 \times (3 \times 10^{-6}) = 0.3 \times 10^{-6}$

Mole ratio CO_2/2-aminobenzonitrile = $(1.56 \times 10^{-6}) / (0.3 \times 10^{-6}) = 5.2$

3.3.5. Stability test of [HDBU$^+$][TFE$^-$] immobilized microreactor: To test the stability of [HDBU$^+$][TFE$^-$]-ILs immobilized selectively on the thimble of SiNWs, the microreactor was run continuously for four days with flow rate 3 μL min^{-1} of reactant 2-aminobenzonitrile (0.1 M) and 35 μL min^{-1} of pure CO_2, and the product was collected at daily base. In the beginning of the 1st to 2nd days, yields of product fall gradually from 97 to 90% then became constant at ~88%, as shown in **Supplementary Fig. 30**.

3.3.6. Product precipitation in an integrated continuous-flow manner: After the completion of reaction, quinazoline-2,4-(1*H*,3*H*)-dione was precipitated continuously by

adding DCM (flow rate = 20 μL min^{-1}) through T-mixer (T1) and passed through a PTFE tubing (id = 500 μm, length = 65 cm) to complete precipitation process during 2.7 min residence time with no clogging problem. Finally, the quinazoline-2,4-(1*H*,3*H*)-dione was filtered through Whatman (pore size 11 μm) filter paper and product was characterized by GC-MS and NMR analysis after drying. The ^{1}H and ^{13}C NMR spectra of all quinazoline-2,4-(1*H*,3*H*)-diones are shown in **Supplementary Fig. 46-59**.

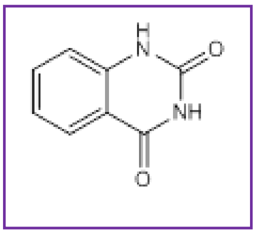

quinazoline-2,4(1*H*,3*H*)-dione (3a): pale yellow solid, ^{1}H NMR (DMSO, 300MHz) δ: 11.16 (b, 2H), 7.83 (d, 1H), 7.59 (dd, 1H), 7.13 (d, 2H); ^{13}C NMR (DMSO, 150MHz) δ: 163.28, 150.75, 141.34, 135.41, 127.41, 122.77, 115.78, 114.80. The observed characterization data (^{1}H and ^{13}C) were consistent with reported literature[11].

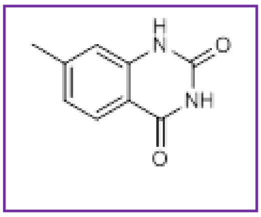

7-methylquinazoline-2,4(1*H*,3*H*)-dione (3b): pale yellow solid, ^{1}H NMR (DMSO, 300MHz) δ: 11.11 (b, 2H), 7.76 (d, 1H), 6.99 (d, 1H), 6.92 (s, 1H), 2.34 (s, 3H); ^{13}C NMR (DMSO, 150MHz) δ: 163.16, 150.92, 146.05, 141.43, 127.38, 124.11, 115.53, 112.52, 21.90. The observed characterization data (^{1}H and ^{13}C) were consistent with reported literature[11].

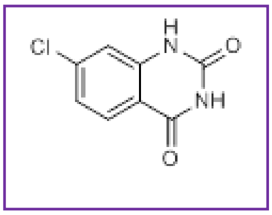

7-chloroquinazoline-2,4(1*H*,3*H*)-dione (3c): white solid, ^{1}H NMR (DMSO, 300MHz) δ: 11.29 (b, 2H), 7.86 (d, 1H), 7.20 (d, 1H), 7.15 (s, 1H); ^{13}C NMR (DMSO, 150MHz) δ: 162.39, 150.74, 142.32, 139.62, 129.35, 123.14, 115.34, 113.68. The observed characterization data (^{1}H and ^{13}C) were consistent with reported literature[11].

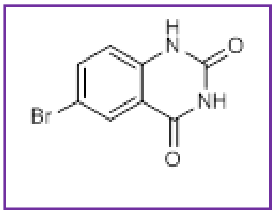

6-bromoquinazoline-2,4(1*H*,3*H*)-dione (3d): pale yellow solid, ^{1}H NMR (DMSO, 300MHz) δ: 11.36 (b, 2H), 7.94 (s, 1H), 7.79 (d, 1H),

7.14 (d, 1H); ^{13}C NMR (DMSO, 150MHz) δ: 162.16, 150.36, 140.48, 137.85, 129.38, 118.18, 116.65, 114.16. The observed characterization data (^{1}H and ^{13}C) were consistent with reported literature[11].

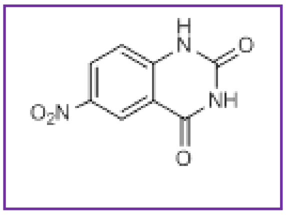

6-nitroquinazoline-2,4(1*H*,3*H*)-dione (3e): yellow solid, ^{1}H NMR (DMSO, 300MHz) δ: 11.73 (b, 2H), 8.59 (s, 1H), 8.46 (d, 1H), 7.30 (d, 1H); ^{13}C NMR (DMSO, 150MHz) δ: 162.11, 150.70, 146.44, 142.41, 129.98, 123.59, 117.20, 114.91.

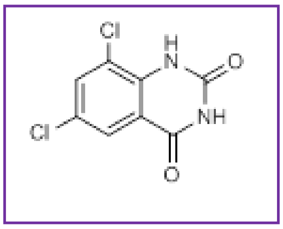

6,8-dichloroquinazoline-2,4(1*H*,3*H*)-dione (3f): pale yellow solid, ^{1}H NMR (DMSO, 300MHz) δ: 11.64 (b, 1H), 10.87 (b, 1H), 7.95 (s, 1H), 7.81 (s, 1H); ^{13}C NMR (DMSO, 150MHz) δ: 161.76, 150.19, 137.46, 134.81, 126.79, 125.68, 120.74, 117.84. The observed characterization data (^{1}H and ^{13}C) were consistent with reported literature[11].

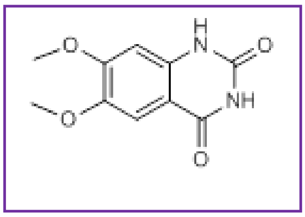

6,7-dimethoxyquinazoline-2,4(1*H*,3*H*)-dione (3g): yellow solid, ^{1}H NMR (DMSO, 300MHz) δ: 10.80-11.15 (b, 2H), 7.24 (s, 1H), 6.66 (s, 1H), 3.75 (d, 6H); ^{13}C NMR (DMSO, 150MHz) δ: 162.93, 155.42, 150.98, 145.60, 137.04, 107.62, 106.57, 98.19, 56.27. The observed characterization data (^{1}H and ^{13}C) were consistent with reported literature[11].

4. Supplementary References

1 Ko, D.-H. *et al.* Superamphiphobic Silicon-Nanowire-Embedded Microsystem and In-Contact Flow Performance of Gas and Liquid Streams. *ACS Nano* **10**, 1156-1162, (2016).

2 Singh, A. K. *et al.* Micro-total envelope system with silicon nanowire separator for safe carcinogenic chemistry. *Nat. Commun.* **7**, (2016).

3 Beckman, E. J. & Munshi, P. Ambient carboxylation on a supported reversible CO_2 carrier: ketone to [small beta]-keto ester. *Green Chem.* **13**, 376-383, (2011).

4 Basavaraju, K. C., Sharma, S., Singh, A. K., Im, D. J. & Kim, D.-P. Chitosan-Microreactor: A Versatile Approach for Heterogeneous Organic Synthesis in Microfluidics. *ChemSusChem* **7**, 1864-1869, (2014).

5 Li, M., Kim, D.-P., Jeong, G.-Y., Seo, D.-K. & Park, C.-P. Reductive surface synthesis of gold nanoparticles on silicate glass and their biochemical sensor applicationsa). *Biomicrofluidics* **6**, 044111, (2012).

6 Yoo, W.-J. & Li, C.-J. Copper-Catalyzed Four-Component Coupling between Aldehydes, Amines, Alkynes, and Carbon Dioxide. *Adv. Synth. Catal.* **350**, 1503-1506, (2008).

7 Hu, J. *et al.* Transformation of Atmospheric CO_2 Catalyzed by Protic Ionic Liquids: Efficient Synthesis of 2-Oxazolidinones. *Angew. Chem. Int. Ed.* **54**, 5399-5403, (2015).

8 Cheng-Hsiu Yu, C.-H. H., Chung-Sung Tan. A Review of CO_2 Capture by Absorption and Adsorption. *Aerosol Air Qual. Res.* **12**, 745-769, (2012).

9 Sharma, S., Maurya, R. A., Min, K.-I., Jeong, G.-Y. & Kim, D.-P. Odorless Isocyanide Chemistry: An Integrated Microfluidic System for a Multistep Reaction Sequence. *Angew. Chem. Int. Ed.* **52**, 7564-7568, (2013).

10 Maurya, R. A., Min, K.-I. & Kim, D.-P. Continuous flow synthesis of toxic ethyl diazoacetate for utilization in an integrated microfluidic system. *Green Chem.* **16**, 116-120, (2014).

11 Zhao, Y. *et al.* A Protic Ionic Liquid Catalyzes CO_2 Conversion at Atmospheric Pressure and Room Temperature: Synthesis of Quinazoline-2,4(1H,3H)-diones. *Angew. Chem. Int. Ed.* **53**, 5922-5925, (2014).
